# Supplementary material for: Dispositional mindfulness and mental health in Chinese emerging adults: A multilevel model with emotion dysregulation as a mediator
Source: PLoS One. 2020 Nov 19;15(11):e0239575. doi: 10.1371/journal.pone.0239575 (PMC7676716; doi:10.1371/journal.pone.0239575)
Supplement: Attachment — Submitted filename: PONE-D-20-06030_reviewer.pdf [file pone.0239575.s002.pdf]

PLOS ONE

Mindful Awareness and Mental Health: A Multilevel Model with Emotion Regulation as a Mediator

--Manuscript Draft--

**Manuscript Number:**	PONE-D-20-06030
**Article Type:**	Research Article
**Full Title:**	Mindful Awareness and Mental Health: A Multilevel Model with Emotion Regulation as a Mediator
**Short Title:**	Mindful Awareness and Mental Health
**Corresponding Author:**	Rebecca Y. M. Cheung<br>The Education University of Hong Kong<br>Hong Kong, HONG KONG
**Keywords:**	emotion regulation, mental health mindful awareness, multilevel modeling, subjective well-being
**Abstract:**	Using a multilevel model, this study examined emotion regulation as a mediator between mindful awareness and mental health among Chinese emerging adults. Participants were 191 Chinese emerging adults (female = 172) between 18 and 27 years old, who completed a questionnaire for three times over 12 months, with a three-month lag between each time point. Within-person analysis revealed that emotion regulation mediated between mindful awareness and mental health outcomes, including subjective well-being and symptoms of depression and anxiety. Time was negatively associated with emotion regulation and positively associated with symptoms of depression and anxiety. Between-person analysis revealed that emotion regulation negatively mediated between mindful awareness and symptoms of depression and anxiety, but not subjective well-being. These findings call attention to emotion regulation as a mediator between mindful awareness and psychological outcomes, particularly of symptoms of depression and anxiety. Notably, the multilevel analysis offered a more nuanced approach to distinguish between within-person vs. between-person processes. Attesting to the relations established in western societies, these findings are applicable to emerging adults in the Chinese context. Evidence was advanced to inform translational research efforts that promote mindful awareness as an asset of mental health.
**Order of Authors:**	Rebecca Y. M. Cheung
	Zijun Ke
	Melody C. Y. Ng
**Additional Information:**	
**Question**	**Response**
**Financial Disclosure**<br><br>Enter a financial disclosure statement that describes the sources of funding for the work included in this submission. Review the submission guidelines for detailed requirements. View published research articles from *PLOS ONE* for specific examples.<br><br>This statement is required for submission and **will appear in the published article** if the submission is accepted. Please make sure it is accurate.	This study was funded by two Internal Grants from The Education University of Hong Kong awarded to RYMC (RG 60/2014-2015R and 04190).

Powered by Editorial Manager® and ProduXion Manager® from Aries Systems Corporation

**Unfunded studies**<br>Enter: *The author(s) received no specific funding for this work.*<br><br>**Funded studies**<br>Enter a statement with the following details:<br>• Initials of the authors who received each award<br>• Grant numbers awarded to each author<br>• The full name of each funder<br>• URL of each funder website<br>• Did the sponsors or funders play any role in the study design, data collection and analysis, decision to publish, or preparation of the manuscript?<br>• **NO** - Include this sentence at the end of your statement: *The funders had no role in study design, data collection and analysis, decision to publish, or preparation of the manuscript.*<br>• **YES** - Specify the role(s) played.<br><br>* typeset	
**Competing Interests**<br><br>Use the instructions below to enter a competing interest statement for this submission. On behalf of all authors, disclose any competing interests that could be perceived to bias this work—acknowledging all financial support and any other relevant financial or non-financial competing interests.<br><br>This statement **will appear in the published article** if the submission is accepted. Please make sure it is accurate. View published research articles from *PLOS ONE* for specific examples.	The authors have declared that no competing interests exist.

Powered by Editorial Manager® and ProduXion Manager® from Aries Systems Corporation

**NO authors have competing interests**<br><br>Enter: *The authors have declared that no competing interests exist.*<br><br>**Authors with competing interests**<br><br>Enter competing interest details beginning with this statement:<br><br>*I have read the journal's policy and the authors of this manuscript have the following competing interests: [insert competing interests here]*<br><br>* typeset	
**Ethics Statement**<br><br>Enter an ethics statement for this submission. This statement is required if the study involved:<br><br>• Human participants<br>• Human specimens or tissue<br>• Vertebrate animals or cephalopods<br>• Vertebrate embryos or tissues<br>• Field research<br><br>Write "N/A" if the submission does not require an ethics statement.<br><br>General guidance is provided below. Consult the submission guidelines for detailed instructions. **Make sure that all information entered here is included in the Methods section of the manuscript.**	Institutional ethics committee that approved the study: Human Research Ethics Committee, The Education University of Hong Kong (Approval #: 2015-2016-0352). A written consent had been obtained from each participant prior to the beginning of the study.

Powered by Editorial Manager® and ProduXion Manager® from Aries Systems Corporation

**Format for specific study types**<br><br>**Human Subject Research (involving human participants and/or tissue)**<br>• Give the name of the institutional review board or ethics committee that approved the study<br>• Include the approval number and/or a statement indicating approval of this research<br>• Indicate the form of consent obtained (written/oral) or the reason that consent was not obtained (e.g. the data were analyzed anonymously)<br><br>**Animal Research (involving vertebrate animals, embryos or tissues)**<br>• Provide the name of the Institutional Animal Care and Use Committee (IACUC) or other relevant ethics board that reviewed the study protocol, and indicate whether they approved this research or granted a formal waiver of ethical approval<br>• Include an approval number if one was obtained<br>• If the study involved *non-human primates*, add *additional details* about animal welfare and steps taken to ameliorate suffering<br>• If anesthesia, euthanasia, or any kind of animal sacrifice is part of the study, include briefly which substances and/or methods were applied<br><br>**Field Research**<br><br>Include the following details if this study involves the collection of plant, animal, or other materials from a natural setting:<br>• Field permit number<br>• Name of the institution or relevant body that granted permission	
**Data Availability**<br><br>Authors are required to make all data underlying the findings described fully available, without restriction, and from the time of publication. PLOS allows rare exceptions to address legal and ethical concerns. See the PLOS Data Policy and FAQ for detailed information.	Yes - all data are fully available without restriction

A Data Availability Statement describing where the data can be found is required at submission. Your answers to this question constitute the Data Availability Statement and **will be published in the article**, if accepted.<br><br>**Important:** Stating 'data available on request from the author' is not sufficient. If your data are only available upon request, select 'No' for the first question and explain your exceptional situation in the text box.<br><br>Do the authors confirm that all data underlying the findings described in their manuscript are fully available without restriction?	
**Describe where the data may be found in full sentences. If you are copying our sample text, replace any instances of XXX with the appropriate details.**<br><br>• If the data are **held or will be held in a public repository**, include URLs, accession numbers or DOIs. If this information will only be available after acceptance, indicate this by ticking the box below. For example: *All XXX files are available from the XXX database (accession number(s) XXX, XXX.)*.<br>• If the data are all contained **within the manuscript and/or Supporting Information files**, enter the following: *All relevant data are within the manuscript and its Supporting Information files.*<br>• If neither of these applies but you are able to provide **details of access elsewhere**, with or without limitations, please do so. For example:<br><br>*Data cannot be shared publicly because of [XXX]. Data are available from the XXX Institutional Data Access / Ethics Committee (contact via XXX) for researchers who meet the criteria for access to confidential data.*<br><br>*The data underlying the results presented in the study are available from (include the name of the third party*	All relevant data are within the manuscript and its Supporting Information files.

and contact information or URL).<br>• This text is appropriate if the data are owned by a third party and authors do not have permission to share the data.<br><br>* typeset	
Additional data availability information:	

Manuscript

Click here to download Manuscript Manuscript 1-3-2020 [Table & Fig Title Included].docx

Dispositional Mindful Awareness and Mental Health in a sample of ....: A Multilevel Model with Emotion Dysregulation as a Mediator.

Title arose some expectations that were not accomplished later

Mindful Awareness and Mental Health: A Multilevel Model with Emotion Regulation as a Mediator

DERS: Difficulties in ER Scale measures emotional dysregulation rather than emotion regulation.

Dysregulation: A failure to regulate properly.

Rebecca Y. M. Cheung[1*], Zijun Ke[2*], Melody C. Y. Ng[3]

Many authors have pointed out that these mindful scales are not measuring Mindful activities, they assess dispositional mindful activities

[1] Department of Early Childhood Education, Centre for Child and Family Studies, and Centre for Psychosocial Health, The Education University of Hong Kong, Hong Kong SAR

[2] Department of Psychology, Sun Yat-Sen University, Guangdong, China

[3] Department of Psychology, The Chinese University of Hong Kong, Hong Kong SAR

[*] Corresponding author

Email (Rebecca Y. M. Cheung): rymcheung@eduhk.hk

Email (Zijun Ke): keziyun@mail.sysu.edu.cn

Provide here M and SD

Abstract

Using a multilevel model, this study examined emotion regulation as a mediator between mindful awareness and mental health among Chinese emerging adults. Participants were 191 Chinese emerging adults (female = 172) between 18 and 27 years old, who completed a questionnaire for three times over 12 months, with a three-month lag between each time point. Within-person analysis revealed that emotion regulation mediated between mindful awareness and mental health outcomes, including subjective well-being and symptoms of depression and anxiety. Time was negatively associated with emotion regulation and positively associated with symptoms of depression and anxiety. Between-person analysis revealed that emotion regulation negatively mediated between mindful awareness and symptoms of depression and anxiety, but not subjective well-being. These findings call attention to emotion regulation as a mediator between mindful awareness and psychological outcomes, particularly of symptoms of depression and anxiety. Notably, the multilevel analysis offered a more nuanced approach to distinguish between within-person vs. between-person processes. Attesting to the relations established in western societies, these findings are applicable to emerging adults in the Chinese context. Evidence was advanced to inform translational research efforts that promote mindful awareness as an asset of mental health.

A questionnaire about what?

Keywords: emotion regulation, mental health mindful awareness, multilevel modeling, subjective well-being

Introduction

Mindfulness is a mental state whereby individuals attend to their cognitive, physical, and emotional experience in the present moment nonjudgmentally [1-2]. Previous research suggested that mindfulness is associated with a number of adjustment outcomes, including better physiological health and well-being, and fewer depressive and anxiety symptoms [3-5]. Moving beyond the simple association between mindfulness and mental health, recent studies have identified several mediating psychological mechanisms, including greater self-esteem [6], more positive affect, hope, and optimism [7], lower rumination [8], and more adaptive emotion regulation [9-10], thereby suggesting possible chains of processes between mindfulness and mental health. Of note, emotion regulation has received much theoretical and empirical attention as a mediating mechanism [11-12].

Aoid using regulating emotions in the definition of emotion regulation

Emotion regulation is defined as the process in regulating emotions and emotional responses [13-14]. According to Gratz and Roemer [15], adaptive emotion regulation involves such aspects as acceptance of emotional responses, engagement in goal directed behavior, maintenance of emotional clarity and awareness, and access to adaptive emotion regulation strategies, such as cognitive reappraisal and savoring ability. On the contrary, maladaptive emotion regulation involves difficulties in acquiring regulatory skills or a frequent use of maladaptive strategies, such as rumination, avoidance, and expressive suppression [16-17]. Previous research suggested that our capability to adaptively regulate emotions is central to subjective well-being [17]. Emotion dysregulation not only undermines well-being, but also gives rise to clinical depression, anxiety, psychological distress, and elevated depressive and anxiety symptoms [9, 16, 18-20].

As a proximal correlate of mental well-being, people's capability to regulate emotions is

associated with mindfulness [5, 21-22]. More specifically, Teper et al. [12] theorized that mindfulness is linked to emotion regulation through heightened metacognitive awareness, non-judgmental acceptance, and executive control, all of which are crucial to well-being. Likewise, in mindfulness-to-meaning theory, Garland et al. [11] postulated that mindfulness promotes purpose in life by virtue of greater metacognitive awareness, broadened attention to context, adaptive emotion regulation, ability to savor hedonic experience, and prosocial actions. Both theoretical frameworks suggest close connections between mindfulness, emotion regulation, and psychological stwell-being.

You see? Dispositional Mindfulness is more accurate

Supporting the frameworks, cross-sectional studies revealed that emotion regulation mediated between dispositional mindfulness and mental health, such as lower levels of depression and anxiety [10, 22-24], and better life satisfaction [25]. Findings based on a clinically depressed and anxious sample suggested that emotion regulation strategies, including cognitive reappraisal and rumination, mediated between dispositional mindfulness and symptoms of depression and anxiety [24]. These findings resonated with research conducted with non-clinical emerging adult samples, in that emotion regulation mediated the inverse relation between dispositional mindfulness and depressive symptoms, such that dispositional mindfulness was related to better emotion regulation and fewer depressive symptoms [22]. In a similar vein, Coffey and Hartman [10] and Coffey et al. [23] found that emerging adults' regulation of negative emotions, impulse control, and goal engagement mediated the link between dispositional mindfulness and well-being. Altogether, evidence to-date converged to indicate emotion regulation as a vital process between dispositional mindfulness and well-being.

Processes of Mental Health in the Chinese Context

Among the handful of studies conducted in the Chinese context, mindfulness predicted

better impulse control, better emotion regulation, less procrastination, and fewer psychological distress [26]. Additionally, mindfulness training increased Chinese adolescents' and adults' well-being and psychological adjustment in Hong Kong [27-28]. In a randomized controlled trial involving a Chinese sample with clinical anxiety, Wong et al. [29] revealed that participants who took an 8-week mindfulness-based cognitive therapy had an increased dispositional mindfulness and a reduced anxiety more significantly than did participants who received psycho-education based on cognitive behavioral therapy principles. Similar findings were suggested in another randomized controlled trial involving a Chinese sample with recurrent symptoms of depression and anxiety, in which participants who received an 8-week compassion–mindfulness therapy showed more mental health improvements than did participants from the waitlist control group [30]. Similar to mindfulness, emotion regulation was also demonstrated as a viable predictor of mental health among Chinese individuals [31-32]. These findings highlighted that the mechanisms associated with mindfulness are integral to mental health in the Chinese context.

Within- vs. Between-Subject Effects

Agree but this section above is a little bit tangential to your research. You did not implement a RCT MBI

A majority of research to-date focuses on between-person associations of mindfulness and mental health. Only a handful of studies partitioned within-person from between-person processes. Between- and within-person effects are conceptually and statistically independent. Within-person effect aids the understanding of *intraindividual* processes underlying well-being. For instance, when mindful awareness of person i increases, then his or her own mental health is expected to also increase over time. Between-person effect accounts for *interindividual* processes underlying well-being. When person i has a higher score in mindful awareness than person j, then person i is expected to have a higher score in mental health than is person j. Partitioning between within- and between-person associations is important, because otherwise the results

could be biased [33] or uninterpretable [34].

In his within-person analyses, Galla [35] indicated that mindfulness meditation training was associated with significant reductions in rumination and depressive symptoms, and increased life satisfaction in healthy adolescents following a five-day mindfulness training. In addition, many of the improvements were maintained at the three-month follow-up assessment. In another study, an 8-week mindfulness training gave rise to an inverse within-person association between mindfulness and relationship stress [36]. Finally, apart from emotion regulation and emotional well-being, within-person associations were found between dispositional mindfulness and relationship satisfaction [37]. By teasing apart within-person from between-person effects, these studies methodologically precluded confounding between-person factors from the within-person associations between mindfulness and other psychological processes.

This Study

Dysregulation

This study aims to investigate longitudinal within- vs between-person mediating effect between mindful awareness and mental health outcomes via emotion regulation. Through this novel analytic technique, the intraindividual processes and the interindividual processes could be identified. Based on the literature [22-24], we hypothesized that the relation between mindful awareness and mental health would be mediated by greater emotion regulation at both within- and between-person levels. However, the strength of associations may vary as a function of the level. At each level, mindful awareness, emotion regulation, and mental health may be linked to covariates including time and gender.

Method

Participants were 191 Chinese emerging adults (female = 172) recruited online through mass emailing at a major public university in Hong Kong. Participants were at 18-27 years old,

with a mean age of 21.06 years at Time 1 (*SD* = 2.01 years). As for the retention rate, 93.72% (*n* = 179) of the participants from Time 1 (T1) were retained at Time 2 (T2); 94.41% (*n* = 169) of the participants from T2 were retained at Time 3 (T3).

Does this Committee a code?

Procedure

The study was approved by the Human Research Ethics Committee prior to its implementation. Informed consent was obtained prior to the administration of the questionnaire. All procedures performed were in accordance with the ethical standards of the institutional research committee and the 1964 Helsinki declaration and its later amendments. Upon informed consent, participants completed baseline measures. They were invited to complete follow-up questionnaires twice, with a three-month lag between time points, over the course of a year. Participants received a supermarket coupon at each time point (totaling HK$200, i.e., ~US$25.71).

This tool measures dispositional mindful awareness

Measures

Mindful Awareness. The 15-item Mindful Attention Awareness Scale (MAAS) [38] was used to assess mindful awareness on a 6-point scale from 1 (*almost always*) to 5 (*almost never*). A sample item included, “I find it difficult to stay focused on what’s happening in the present.” The item scores were averaged, such that higher scores indicated a greater level of mindful awareness. In this study, Cronbach’s alpha = .91-.94 at T1-T3.

Emotion Regulation. The 36-item Difficulties in Emotional Regulation Scale (DERS) [15] was used to assess emotional regulation on a 5-point scale from 1 (*almost never*) to 5 (*almost always*). The measure included 6 subscales including non-acceptance of emotions, difficulties in engaging in goal-directed behavior, impulse control difficulties, lack of emotional

awareness, limited access to emotion regulation strategies, and lack of emotional clarity. Scores of the negative items were reversed, such that final scores reflected a greater ability in emotion regulation. Sample items included, "I am clear about my feelings" and "When I'm upset, I believe that my feelings are valid and important." The final scores were then averaged, such that higher scores indicated a greater ability in emotion regulation. Cronbach's alpha = .83-.87 at T1-T3.

Cronbach's alpha os subscles should be also reported. You can use intercorrelations at T1, T2 and T3 as well.

Depressive Symptoms. The 20-item Center for Epidemiologic Studies Depression Scale (CES-D) [39] was used to measure depressive symptoms in the past week on a 4-point scale from 1 (*rarely or none of the time, less than 1 day*) to 4 (*most or all of the time, 5-7 days*). A sample item included, "I felt depressed." The item scores were summed to form a composite score of depressive symptoms, with higher scores indicating more symptoms. In this study, Cronbach's alpha = .87-.91 at T1-T3.

Is there a cut-off in the CES-D scoring to indicate depression?

Anxiety Symptoms. The 7-item Generalized Anxiety Disorder-7 measure (GAD-7) [40] was used to measure depressive symptoms in the past two weeks on a 4-point scale from 0 (*not at all*) to 3 (*nearly every day*). A sample items included, "Feeling nervous, anxious or on edge." The item scores were averaged to form a composite score of anxiety symptoms, with higher scores indicating more symptoms. In this study, Cronbach's alpha = 93-.95 at T1-T3.

Subjective Well-being. The 14-item Mental Health Continuum Short Form (MHC-SF) [41] was used as a measure for well-being over the last month. A 6-point scale ranging from 0 (*never*) to 5 (*every day*) was used, with sample items including, "how often did you feel satisfied with life" (*emotional well-being*), "how often did you feel that that you had something important to contribute to society (*social well-being*) and "how often did you feel that your life has a sense of direction or meaning to it" (*psychological well-being*). In this study, Cronbach's

alpha = .79-.94 at T1-T3.

Data Analysis

Multilevel mediation analyses were conducted using Mplus 8.0 [42] to study the mediating effect of emotional regulation on the relationship between mindful awareness and mental health outcomes, including depressive symptoms, anxiety symptoms, and subjective well-being, at both the within- and between-person levels. Detailed descriptions about the multilevel mediation model under consideration are available upon request. Multilevel modeling has been used in previous research for panel data (e.g., [43]), with a minimum of three waves of observations to partition between within- and between-person associations to reduce biases [33] and ensure that the findings were interpretable [34]. A typical application of multilevel modeling for panel data is growth curve modeling (GCM) [44]. With time-varying covariates (covariates at level-1), GCM allows researchers to study the relationships between variables in addition to the effects of time [43,45].

In this study, covariates at both levels were considered. At the within-person level, a time variable was included to control for the potential change over time [46]. At the between-person level, gender was included to control for potential gender differences concerning symptoms of anxiety and depression [47-49]. With only three measurement occasions at level 1, the model allowing all mediation paths (i.e., “a” path from mindful awareness to emotion regulation, “b” paths from mindful awareness to mental health, and “c” paths from emotion regulation to mental health) and the slope of time to be random was not identified. Therefore, we tested possible random mediation paths sequentially. Results showed that none of the “a”, “b”, and “c′” paths showed substantial individual differences, except for the “c′” path for depression. Additionally, we tested possible random slopes of time (change between two consecutive measurement

this in not a typo

occasions) and found no significant individual differences. Consequently, all mediation paths and slopes of time were fixed to be invariant across participants except for the "c′" path for depression in the subsequent analyses.

Following the traditional steps of mediation analysis [50], we fitted a multilevel mediation model without the mediator to study the overall effect of mindful awareness on the outcomes. The model was estimated under missing data theory using all available data [42, ch.9]. Given the limitations listed by Hayes [51], the ratio of the indirect effect to the total effect was not calculated in determining the strength of the indirect effect. That is, this ratio could be out of the expected range between 0 and 1, and the estimate of the ratio might be highly unstable from sample to sample. Unless the sample size was fairly large, Hayes [50, p.189] recommended not "having much faith" in this measure.

Results

Table 1 summarizes the means, *SD*s, and correlations among the study variables. The variables showed substantial within-person correlations, which were captured by the random intercepts in the multilevel model. Changes in means over time were observed, suggesting the necessity of including time as a covariate.

Table 1. Zero-Order correlations, means, and standard deviations of the study variables (N = 191)

Variable	(1)	(2)	(3)	(4)	(5)	(6)	(7)	(8)	(9)	(10)	(11)	(12)	(13)	(14)	(15)	(16)
(1) Gender	-															
Time 1																
(2) Mindful awareness	[illegible]															
(3) Emotion regulation	.09	-.55***	-													
(4) Depressive symptoms	-.02	-.48***	-.69***	-												
(5) Anxiety symptoms	-.11	-.42***	-.64***	.77***	-											
(6) Subjective well-being	.10	.29***	.30***	-.36***	-.26***	-										
Time 2																
(7) Mindful awareness	.19*	.58***	.54***	-.41***	-.37***	.28***	-									
(8) Emotion regulation	.03	.42***	.76***	-.63***	-.59***	.26***	.62***	-								
(9) Depressive symptoms	-.05	-.23**	-.49***	.67***	.57***	-.17*	-.47***	-.63***	-							
(10) Anxiety symptoms	-.09	-.21**	-.39***	.51***	.52***	-.31***	-.47***	-.52***	.73***	-						
(11) Subjective well-being	.21**	.35***	.34***	-.37***	-.28***	.65***	.50***	.37***	-.32***	-.39***	-					
Time 3																
(12) Mindful awareness	.17*	.53***	.47***	-.42***	-.38***	.29***	.58***	.54***	-.45***	-.38***	.39***	-				
(13) Emotion regulation	.12	.42***	.70***	-.55***	-.48***	.25**	.49**[illegible]	.75***	-.52***	-.38***	.35***	.65***	-			
(14) Depressive symptoms	-.16*	-.45***	-.53***	.62***	.57***	-.34***	-.45***	-.56***	.67***	.54***	-.37***	-.61***	-.68***	-		
(15) Anxiety symptoms	-.16*	-.39***	-.44***	.53***	.54***	-.24**	-.36***	-.48***	.56***	.56***	-.30***	-.49***	-.59***	.80***	-	
(16) Subjective well-being	.23**	.31***	.30***	-.33***	-.22**	.72***	.45***	.33***	-.35***	-.37***	.77***	.47***	.37***	-.48***	-.36***	-
M	.90	2.85	2.97	.61	.61	4.28	2.80	2.88	.68	.65	4.17	2.78	2.88	.74	.81	4.25
SD	.30	.43	.49	.50	.58	1.34	.48	.51	.56	.64	1.30	.52	.54	.61	.68	1.43

Note. $^{*}p < .05$, $^{**}p < .01$, $^{***}p < .001$.

negative then dysregulation is more appropriate

Gender: 0 male and 1 female?
Why correlations of MAAS were moderate low at T1, T2, T3

According to this correlation Gender could be a moderator

M and SD seem to be averaged, this should be notified in the table and put in the first column the range of every single instrument

Multilevel structural equation modeling

ICC is more appropriate to assess the level of agreement among raters. Where is reported the ICC, Table 1 is not a ICC

The intraclass correlations (ICC) of the studied variables, which quantified the proportion of variance in the variables attributable to individual differences, ranged from .52 (anxiety) to .73 (emotion regulation). This indicated substantial between-person variances, which should be considered using multilevel modeling. The design effect, defined as 1-(average cluster size-1)×ICC, was larger than two for any of the studied variables, suggesting that the hierarchical structure of the data should be considered [52]. Based on the R-squared measures for multilevel models proposed by Rights and Sterba [53], we computed the proportion of within-cluster outcome variance explained by level-1 predictors via fixed slopes and random slope variation (i.e., $R_w^{2(f_{1v})}$ in the paper) and the proportion of between-cluster outcome variance explained by level-2 predictors via fixed slopes (i.e., $R_b^{2(f_2)}$ in the paper) for each of the three outcome variables. Specifically, mindful awareness, emotional regulation, and time explained 23% of the within-person variances via fixed slopes and random slope variation for depression, and 18% and 30% of the within-person variances via fixed slopes for anxiety and well-being, respectively. At the between-person level, mindful awareness, emotional regulation and gender explained 31% and 42% of the between-person variances for the first two outcome variables, respectively. Mindful awareness and emotional regulation explained 67% of the between-person variances for well-being (see Figure 1).

Figure 1. Multilevel mediation model of mindful attention awareness, emotion regulation, and mental health outcomes. *p =/< .05, **p =/< .01, ***p =/< .001. Unstandardized parameter estimates and standard errors in parentheses are presented.

Within-person indirect effect of mindful awareness. The effect of mindful

awareness on depressive symptoms was negatively mediated by emotion regulation, $\widehat{ab} = -.12$, $p < .001$, 95% CI = [-.18, -.06], with a significant mean direct effect, $\hat{c}_0' = -.17$, $p < .01$, 95% CI = [-.30, -.05]. Specifically, enhanced mindful awareness was associated with enhanced emotion regulation, $\hat{a} = .31$, $p < .001$, 95% CI = [.21, .41], which in turn predicted reduced depressive symptoms, $\hat{b} = -.40$, $p < .001$, 95% CI = [-.54, -.26]. The results regarding anxiety symptoms showed similar patterns (see Table 2). Specifically, emotion regulation predicted by greater mindful awareness was associated with reduced anxiety symptoms, $\widehat{ab} = -.15$, $p < .001$, 95% CI = [-.23, -.07], suggesting a partial mediation effect, $\hat{c}' = -.19$, $p = .01$, 95% CI = [-.34, -.04].

Table 2. Unstandardized parameter estimates and standard errors of the multilevel model

Parameter	*Unstandardized B (SE)*
Within-Person Effect	
Time	
→ Mindful awareness	-.03 (.02)
→ Emotion regulation	-.04 (.01)**
→ Depressive symptoms	.04 (.02)*
→ Anxiety symptoms	.07 (.02)**
→ Subjective Well-being	-.12 (.03)***
Mindful awareness	
→ Emotion regulation	.31 (.05)***
→ Depressive symptoms (mean, c_{w10}')	-.17 (.07)**
→ Anxiety symptoms	-.19 (.08)*
→ Subjective Well-being	.60 (.10)***
Emotion regulation	
→ Depressive symptoms	-.40 (.07)***
→ Anxiety symptoms	-.49 (.10)***
→ Subjective well-being	.43 (.12)***
Depressive symptoms ←→ Anxiety symptoms	.06 (.01)***
Depressive symptoms ←→ Subjective Well-being	-.04 (.01)***
Anxiety symptoms ←→ Subjective Well-being	-.04 (.01)**
Between-Person Effect	
Gender	
→ Depressive symptoms	.03 (.09)
→ Anxiety symptoms	-.07 (.11)
Mindful awareness	
→ Emotion regulation	.91 (.09)***
→ Depressive symptoms	.29 (.55)
→ Anxiety symptoms	-.23 (.16)
→ Subjective Well-being	1.44 (.19)***
Emotion regulation	
→ Depressive symptoms	-1.31 (.48)**
→ Anxiety symptoms	-.62 (.14)***

→ Subjective Well-being	.24 (.14)
Depressive symptoms ←→ Anxiety symptoms	.11 (.05)*
Depressive symptoms ←→ Subjective well-being	.01 (.05)
Anxiety symptoms ←→ Subjective well-being	-.05 (.02)**
Depressive symptoms ←→ c'_{w1i}	-.14 (.06)*
Anxiety symptoms ←→ c'_{w1i}	-.02 (.02)
Subjective well-being ←→ c'_{w1i}	-.01 (.02)
Mindfulness ←→ c'_{w1i}	.01 (.01)
Emotion regulation ←→ c'_{w1i}	.02 (.02)
c'_{w1i} ←→ c'_{w1i}	.05 (.02)*

Note. *$p < .05$, **$p < .01$, ***$p < .001$.

Regarding the indirect effect of mindful awareness on well-being through emotion regulation, we found a partial positive mediation effect, $\widehat{ab} = .13, p = .002$, 95% CI = [.05, .22]; $\hat{c}' = .60, p < .001$, 95% CI = [.41, .79]. Specifically, mindful awareness was positively associated with emotion regulation, which in turn predicted better mental health, $\hat{b} = .43, p < .001$, 95% CI = [0.20, .66] (see Table 3).

Table 3. Results of multilevel mediation analyses on the mediating effect of emotion regulation between mindful awareness and mental health outcomes

Outcome	a	b	c'	ab	c
			Within-Person		
Depression		**-.40[-.54,-.26]**	**-.17[-.30,-.05]**	**-.12[-.18,-.06]**	**-.29[-.43,-.16]**
Anxiety	**.31[.21,.41]**	**-.49[-.69,-.29]**	**-.19[-.34,-.04]**	**-.15[-.23,-.07]**	**-.34[-.49,-.19]**
Subjective well-being		**.43[.20,.66]**	**.60[.41,.79]**	**.13[.05,.22]**	**.73[.52,.94]**
			Between-Person		
Depression		**-1.31[-2.26,-.36]**	.29[-.78,1.35]	**-1.19[-2.01,-.38]**	**-.91[-1.53,-.29]**
Anxiety	**.91[.74,1.09]**	**-.62[-.89,-.35]**	-.23[-.54,.08]	**-.57[-.85,-.29]**	**-.80[-1.01,-.59]**
Subjective well-being		.24[-.03,.50]	**1.44[1.07,1.82]**	.22[-.02,.45]	**1.66[1.40,1.93]**

Note. The boldfaced numbers indicate significant results. Numbers within brackets are the lower and upper limits of confidence intervals. The coefficients a, b, c, and c' represent the paths from Mindful awareness to emotion regulation, from emotion regulation to mental health outcomes, and from Mindful awareness to mental health outcomes before and after controlling for the effect of emotion regulation. The coefficient ab represents the indirect effect of emotion regulation. Note that the within-person c' and c paths for depression was the average c' and c paths across participants. The upper panel reports the results regarding the within-person level whereas the lower displays those for the between-person level.

Between-person indirect effect of mindful awareness. The effects of mindful awareness on depression and anxiety symptoms were found to be negatively mediated by emotion regulation (see Table 2). Notably, full mediation effects were found at the between-person level, as indicated by the nonsignificant direct effects of mindful awareness on symptoms of depression, $\hat{c}' = .29, p = .60$, 95% CI = [-.78, 1.35] and on anxiety, $\hat{c}' = -.23, p = .15$, 95% CI = [-.54,.08]. Greater mindful awareness was associated with better emotion regulation, which in turn was associated with fewer symptoms of depression and anxiety.

The between-person indirect effect of mindful awareness on well-being was not significant, $\widehat{ab} = .22, p = .07$, 95% CI = [-.02, .45]. Although the "a" path between mindful awareness and emotion regulation was significant, the "b" path between emotion regulation and well-being was not, $\hat{b} = .24, p = .08$, 95% CI = [-.03, .50] (see Table 3).

Discussion

You did not implement an intervention and the clinical measures should have had a cut-off. The discussion needs to follow a tentative argument

Building on existing theories on mindfulness, emotion regulation, and well-being [11-12], this study generally supported emotion regulation as a mediator between mindful awareness and mental health in Chinese emerging adults. Findings based on multi-level modeling further suggested within- and between-person nuances for the associations. At the within-person level, emotion regulation mediated between mindful awareness and all mental health outcomes. Surprisingly, time was associated with worse emotion regulation and well-being, suggesting that psychological functioning worsened as a function of time. At the between-person level, mindful awareness positively predicted emotion regulation and subjective well-being, whereas emotion regulation negatively predicted symptoms of depression and anxiety, but not subjective well-being. Gender was not related to symptoms of depression and anxiety. These findings advanced the field by establishing within- and between-person relations between mindful awareness and

psychological functioning using a process-oriented approach.

This study demonstrated the utility of multilevel modeling by partitioning between-person relations from within-person relations. Importantly, the variables were related within-person in the hypothesized directions, suggesting emotion regulation as a central intraindividual process linking between mindful awareness and mental health. When mindful awareness of person *i* increased, his/her capability to regulate emotions and subsequent mental health also increased. By orienting to the present moment and paying attention on purpose and non-judgmentally [54], mindfully attentive individuals were more capable of decentering themselves from subjective emotional experience [55], less preoccupied with invalidating, avoiding, or rejecting emotional experiences [56], and more likely to disengage themselves from an "autopilot" status [57]. These characteristics are crucial for emotion regulation and mental health [55].

Time was inversely related to emotion regulation and well-being. Contrary to previous research suggesting that individuals were more capable of emotion regulation as they mature [58], our findings suggested the opposite. It might be that our short-term longitudinal data were insufficient to capture a positive development in emotion regulation. Hence, a long-term longitudinal approach spanning over several years is necessary to verify whether this is the case. Alternatively, given the participants were emerging adults enrolled in a university, they may be facing increasing stress as they approached graduation. Future work is needed to rule out confounding variables, such as academic stress and sample characteristics, associated with time or mental health [59].

Turning to the between-person findings, mindful awareness was directly associated with emotion regulation and subjective well-being, but not with symptoms of depression and anxiety.

These findings signified that when person *i* had a higher score in mindful awareness than person *j*, then person *i* also was more likely to have higher scores in emotion regulation and well-being than person *j*. Despite the lack of a direct relation between mindful awareness and psychological symptoms, as an explanatory variable emotion regulation accounted for the between-person relation. Surprisingly, emotion regulation did not hold up as a between-person mediator between mindful awareness and subjective well-being. Emotion regulation was not related to subjective well-being at all. Future research should examine whether stage-salient variables explain the relation between mindful awareness and well-being. Also, rather than general regulation of emotions, perhaps nuances such as strategies (e.g., expressive suppression) [60], regulatory flexibility [61], and affective states, frequency, and intensity [62] are other factors of mental health. Finally, gender was not related to psychological symptoms in the model. We speculated that our small sample of men might have precluded us from drawing meaningful conclusions about gender as a correlate of psychological distress.

Limitations and Future Directions

The study has several limitations. First, we utilized self-report measures. Future researchers may adopt a multi-method approach by incorporating physiological and neural measures. Next, given this sample comprised mainly of emerging adults at a university, it is uncertain whether the findings can be generalized to other contexts. Future studies should examine the relation in a more representative sample from the community. Finally, our participants were mainly female. Future studies with gender-balanced samples are necessary to draw meaningful conclusions for the effects of gender.

Conclusion

This study calls attention to the relation between mindful awareness and mental health

through emotion regulation. Taking into account the effect of time, findings based on multilevel modeling demonstrated differential effects between the within- and the between-person levels, thereby suggesting a need to partition these levels in future research. The general findings converged to underscore the association between mindful awareness and different aspects of mental health in emerging adulthood. Psychological intervention programs and public health campaigns geared toward enhancing mindful awareness and emotion regulation merit future research investigations.

References

1. Farb NA, Anderson AK, Segal ZV. The mindful brain and emotion regulation in mood disorders. The Canadian Journal of Psychiatry. 2012 Feb;57(2):70-7. doi: 10.1177/070674371205700203
2. Kabat□Zinn J. Mindfulness□based interventions in context: past, present, and future. Clinical psychology: Science and practice. 2003 Jun;10(2):144-56. doi: 10.1093/clipsy.bpg016
3. Baer RA, Lykins EL, Peters JR. Mindfulness and self-compassion as predictors of psychological wellbeing in long-term meditators and matched nonmeditators. The Journal of Positive Psychology. 2012 May 1;7(3):230-8. doi: 10.1080/17439760.2012.674548
4. Bowlin SL, Baer RA. Relationships between mindfulness, self-control, and psychological functioning. Personality and Individual Differences. 2012 Feb 1;52(3):411-5. doi: 10.1016/j.paid.2011.10.050
5. Lyvers M, Makin C, Toms E, Thorberg FA, Samios C. Trait mindfulness in relation to emotional self-regulation and executive function. Mindfulness. 2014 Dec 1;5(6):619-25. doi: 10.1007/s12671-013-0213-y
6. Rasmussen MK, Pidgeon AM. The direct and indirect benefits of dispositional mindfulness on self-esteem and social anxiety. Anxiety, Stress, & Coping. 2011 Mar 1;24(2):227-33. doi: 10.1080/10615806.2010.515681
7. Malinowski P, Lim HJ. Mindfulness at work: Positive affect, hope, and optimism mediate the relationship between dispositional mindfulness, work engagement, and well-being. Mindfulness. 2015 Dec 1;6(6):1250-62. doi: 10.1007/s12671-015-0388-5
8. Petrocchi N, Ottaviani C. Mindfulness facets distinctively predict depressive symptoms

after two years: The mediating role of rumination. Personality and Individual Differences. 2016 Apr 1;93:92-6. doi: 10.1016/j.paid.2015.08.017

9. Cheung RYM, Ng MCY. Mindfulness and symptoms of depression and anxiety: the underlying roles of awareness, acceptance, impulse control, and emotion regulation. Mindfulness. 2019 Jun 15;10(6):1124-35. doi: 10.1007/s12671-018-1069-y
10. Coffey KA, Hartman M. Mechanisms of action in the inverse relationship between mindfulness and psychological distress. Complementary health practice review. 2008 Apr;13(2):79-91. doi: 10.1177/1533210108316307
11. Garland EL, Farb NA, R. Goldin P, Fredrickson BL. Mindfulness broadens awareness and builds eudaimonic meaning: A process model of mindful positive emotion regulation. Psychological Inquiry. 2015 Oct 2;26(4):293-314. doi: 10.1080/1047840X.2015.1064294
12. Teper R, Segal ZV, Inzlicht M. Inside the mindful mind: How mindfulness enhances emotion regulation through improvements in executive control. Current Directions in Psychological Science. 2013 Dec;22(6):449-54. doi: 10.1177/0963721413495869
13. Chiesa A, Serretti A, Jakobsen JC. Mindfulness: Top–down or bottom–up emotion regulation strategy?. Clinical psychology review. 2013 Feb 1;33(1):82-96. doi: 10.1016/j.cpr.2012.10.006
14. Gross JJ. The emerging field of emotion regulation: An integrative review. Review of general psychology. 1998 Sep;2(3):271-99. doi: 10.1037/1089-2680.2.3.271
15. Gratz KL, Roemer L. Multidimensional assessment of emotion regulation and dysregulation: Development, factor structure, and initial validation of the difficulties in emotion regulation scale. Journal of psychopathology and behavioral assessment. 2004 Mar 1;26(1):41-54. doi: 10.1023/B:JOBA.0000007455.08539.94

16. Aldao A, Nolen-Hoeksema S, Schweizer S. Emotion-regulation strategies across psychopathology: A meta-analytic review. Clinical psychology review. 2010 Mar 1;30(2):217-37. doi: 10.1016/j.cpr.2009.11.004
17. Gross JJ, John OP. Individual differences in two emotion regulation processes: implications for affect, relationships, and well-being. Journal of personality and social psychology. 2003 Aug;85(2):348-62. doi: 10.1037/0022-3514.85.2.348
18. Joormann J, Gotlib IH. Emotion regulation in depression: Relation to cognitive inhibition. Cognition and Emotion. 2010 Feb 1;24(2):281-98. doi: 10.1080/02699930903407948
19. Martin RC, Dahlen ER. Cognitive emotion regulation in the prediction of depression, anxiety, stress, and anger. Personality and individual differences. 2005 Nov 1;39(7):1249-60. doi: 10.1016/j.paid.2005.06.004
20. Mennin DS, Heimberg RG, Turk CL, Fresco DM. Preliminary evidence for an emotion dysregulation model of generalized anxiety disorder. Behaviour research and therapy. 2005 Oct 1;43(10):1281-310. doi: 10.1016/j.brat.2004.08.008
21. Hill CL, Updegraff JA. Mindfulness and its relationship to emotional regulation. Emotion. 2012 Feb;12(1):81-90. doi: 10.1037/a0026355
22. Jimenez SS, Niles BL, Park CL. A mindfulness model of affect regulation and depressive symptoms: Positive emotions, mood regulation expectancies, and self-acceptance as regulatory mechanisms. Personality and individual differences. 2010 Oct 1;49(6):645-50. doi: 10.1016/j.paid.2010.05.041
23. Coffey KA, Hartman M, Fredrickson BL. Deconstructing mindfulness and constructing mental health: Understanding mindfulness and its mechanisms of action. Mindfulness. 2010 Dec 1;1(4):235-53. doi: 10.1007/s12671-010-0033-2

24. Desrosiers A, Vine V, Klemanski DH, Nolen□Hoeksema S. Mindfulness and emotion regulation in depression and anxiety: common and distinct mechanisms of action. Depression and anxiety. 2013 Jul;30(7):654-61. doi: 10.1002/da.22124
25. Mandal SP, Arya YK, Pandey R. Mindfulness, Emotion Regulation, and Subjective Well-Being: Exploring the Link. SIS Journal of Projective Psychology & Mental Health. 2017 Jan 1;24(1): 57-63.
26. Cheung RYM, Ng MCY. Being in the moment later? Testing the inverse relation between mindfulness and procrastination. Personality and Individual Differences. 2019 Apr 15;141:123-6. doi: 10.1016/j.paid.2018.12.015
27. Hue MT, Lau NS. Promoting well-being and preventing burnout in teacher education: A pilot study of a mindfulness-based programme for pre-service teachers in Hong Kong. Teacher Development. 2015 Jul 3;19(3):381-401. doi: 10.1080/13664530.2015.1049748
28. Lau NS, Hue MT. Preliminary outcomes of a mindfulness-based programme for Hong Kong adolescents in schools: Well-being, stress and depressive symptoms. International Journal of Children's Spirituality. 2011 Nov 1;16(4):315-30. doi: 10.1080/1364436X.2011.639747
29. Wong SY, Yip BH, Mak WW, Mercer S, Cheung EY, Ling CY, Lui WW, Tang WK, Lo HH, Wu JC, Lee TM. Mindfulness-based cognitive therapy v. group psychoeducation for people with generalised anxiety disorder: randomised controlled trial. The British Journal of Psychiatry. 2016 Jul;209(1):68-75. doi: 10.1192/bjp.bp.115.166124
30. Lo HH, Ng SM, Chan CL. Evaluating compassion–mindfulness therapy for recurrent anxiety and depression: A randomized control trial. Research on Social Work Practice. 2015 Oct;25(6):715-25. doi: 10.1177/1049731514537686

31. Cheung RYM, Leung MC, Chan KK, Lam CB. Effects of mother-offspring and father-offspring dynamics on emerging adults' adjustment: The mediating role of emotion regulation. PloS one. 2019;14(2): e0212331. doi: 10.1371/journal.pone.0212331

32. Zhao Y, Zhao G. Emotion regulation and depressive symptoms: Examining the mediation effects of school connectedness in Chinese late adolescents. Journal of Adolescence. 2015 Apr 1;40:14-23. doi: 10.1016/j.adolescence.2014.12.009

33. Preacher KJ, Zyphur MJ, Zhang Z. A general multilevel SEM framework for assessing multilevel mediation. Psychological methods. 2010 Sep;15(3):209-23. doi: 10.1037/a0020141

34. Raudenbush SW, Bryk AS. Hierarchical linear models: Applications and data analysis methods (2nd ed.). Newbury Park, CA: Sage; 2002.

35. Galla BM. Within-person changes in mindfulness and self-compassion predict enhanced emotional well-being in healthy, but stressed adolescents. Journal of adolescence. 2016 Jun 1;49:204-17. doi: 10.1016/j.adolescence.2016.03.016

36. Carson JW, Carson KM, Gil KM, Baucom DH. Mindfulness-based relationship enhancement. Behavior therapy. 2004 Jun 1;35(3):471-94. doi: 10.1016/S0005-7894(04)80028-5

37. Lenger KA, Gordon CL, Nguyen SP. Intra-individual and cross-partner associations between the five facets of mindfulness and relationship satisfaction. Mindfulness. 2017 Feb 1;8(1):171-80. doi: 10.1007/s12671-016-0590-0

38. Brown KW, Ryan RM. The benefits of being present: mindfulness and its role in psychological well-being. Journal of personality and social psychology. 2003 Apr;84(4):822-48. doi: 10.1037/0022-3514.84.4.822

39. Radloff LS. A self-report depression scale for research in the general population. Applied psychol Measurements. 1977;1:385-401. doi: 10.1177/014662167700100306
40. Spitzer RL, Kroenke K, Williams JB, Löwe B. A brief measure for assessing generalized anxiety disorder: the GAD-7. Archives of internal medicine. 2006 May 22;166(10):1092-7. doi: 10.1001/archinte.166.10.1092
41. Keyes CL. Brief description of the mental health continuum short form (MHC-SF). 2009 [cited 2019 Jan 4]. Available from: http://www.sociology.emory.edu/ckeyes/
42. Muthén, L.K.., & Muthén, B.O. Mplus User's Guide (8th Edition). Los Angeles, CA: Muthén & Muthén; 1998-2017.
43. Park IJ, Wang L, Williams DR, Alegría M. Does anger regulation mediate the discrimination–mental health link among Mexican-origin adolescents? A longitudinal mediation analysis using multilevel modeling. Developmental psychology. 2017 Feb;53(2):340-52. doi: 10.1037/dev0000235
44. Singer JD, Willett JB, Willett JB. Applied longitudinal data analysis: Modeling change and event occurrence. Oxford university press; 2003 Mar 27.
45. Ginexi EM, Howe GW, Caplan RD. Depression and control beliefs in relation to reemployment: What are the directions of effect?. Journal of Occupational Health Psychology. 2000 Jul;5(3):323-36. doi: 10.1037/1076-8998.5.3.323
46. Wang LP, Maxwell SE. On disaggregating between-person and within-person effects with longitudinal data using multilevel models. Psychological methods. 2015 Mar;20(1):63. doi: 10.1037/met0000030
47. Kessler RC, McGonagle KA, Swartz M, Blazer DG, Nelson CB. Sex and depression in the National Comorbidity Survey I: Lifetime prevalence, chronicity and recurrence. Journal of

Affective Disorders. 1993; 29(2-3):85-96. doi: 10.1016/0165-0327(93)90026-G

48. McLean CP, Asnaani A, Litz BT, Hofmann SG. Gender differences in anxiety disorders: prevalence, course of illness, comorbidity and burden of illness. Journal of psychiatric research. 2011 Aug 1;45(8):1027-35. doi: 10.1016/j.jpsychires.2011.03.006
49. Piccinelli M, Wilkinson G. Gender differences in depression: Critical review. The British Journal of Psychiatry. 2000 Dec;177(6):486-92. doi: 10.1192/bjp.177.6.486
50. Baron RM, Kenny DA. The moderator–mediator variable distinction in social psychological research: Conceptual, strategic, and statistical considerations. Journal of personality and social psychology. 1986 Dec;51(6):1173-82. doi: 10.1037/0022-3514.51.6.1173
51. Hayes AF. Introduction to mediation, moderation, and conditional process analysis: A regression-based approach. New York, NY: Guilford Press; 2013.
52. Muthen BO, Satorra A. Complex sample data in structural equation modeling. Sociological methodology. 1995 Jan 1:267-316. doi: 10.2307/271070
53. Rights JD, Sterba SK. Quantifying explained variance in multilevel models: An integrative framework for defining R-squared measures. Psychological methods. 2019 Jun;24(3):309-38. doi: 10.1037/met0000184
54. Kabat-Zinn J. Wherever you go, there you are: Mindfulness meditation in everyday life. New York: Hyperion; 1994.
55. Bhambhani Y, Cabral G. Evaluating nonattachment and decentering as possible mediators of the link between mindfulness and psychological distress in a nonclinical college sample. Journal of evidence-based complementary & alternative medicine. 2016 Oct;21(4):295-305. doi: 10.1177/2156587215607109

56. Hayes AM, Feldman G. Clarifying the construct of mindfulness in the context of emotion regulation and the process of change in therapy. Clinical Psychology: science and practice. 2004 Sep;11(3):255-62. doi: 10.1093/clipsy.bph080

57. Germer C, Siegel RD, Fulton PR, editors. Mindfulness and psychotherapy. New York: Guilford Publications; 2016 Aug 3.

58. Zimmermann P, Iwanski A. Emotion regulation from early adolescence to emerging adulthood and middle adulthood: Age differences, gender differences, and emotion-specific developmental variations. International journal of behavioral development. 2014 Mar;38(2):182-94. doi: 10.1177/016502541351540

59. Pedersen D. Stress carry-over and college student health outcomes. College Student Journal. 2012 Sep 1;46(3):620-7.

60. Sai L, Luo S, Ward A, Sang B. Development of the tendency to use emotion regulation strategies and their relation to depressive symptoms in Chinese adolescents. Frontiers in psychology. 2016 Aug 22;7:1222. doi: 10.3389/fpsyg.2016.01222

61. Bonanno GA, Burton CL. Regulatory flexibility: An individual differences perspective on coping and emotion regulation. Perspectives on Psychological Science. 2013 Nov;8(6):591-612. doi: 10.1177/1745691613504116

62. DeSteno D, Gross JJ, Kubzansky L. Affective science and health: The importance of emotion and emotion regulation. Health Psychology. 2013 May;32(5):474-86. doi: 10.1037/a0030259

Figure

Click here to download Figure Figure 1 (Model Findings) 1-3-2020 (title included).tiff

Figure 1. Multilevel mediation model of mindful attention awareness, emotion regulation, and mental health outcomes. *p =/< .05, **p =/< .01, ***p =/< .001. Unstandardized parameter estimates and standard errors in parentheses are presented.

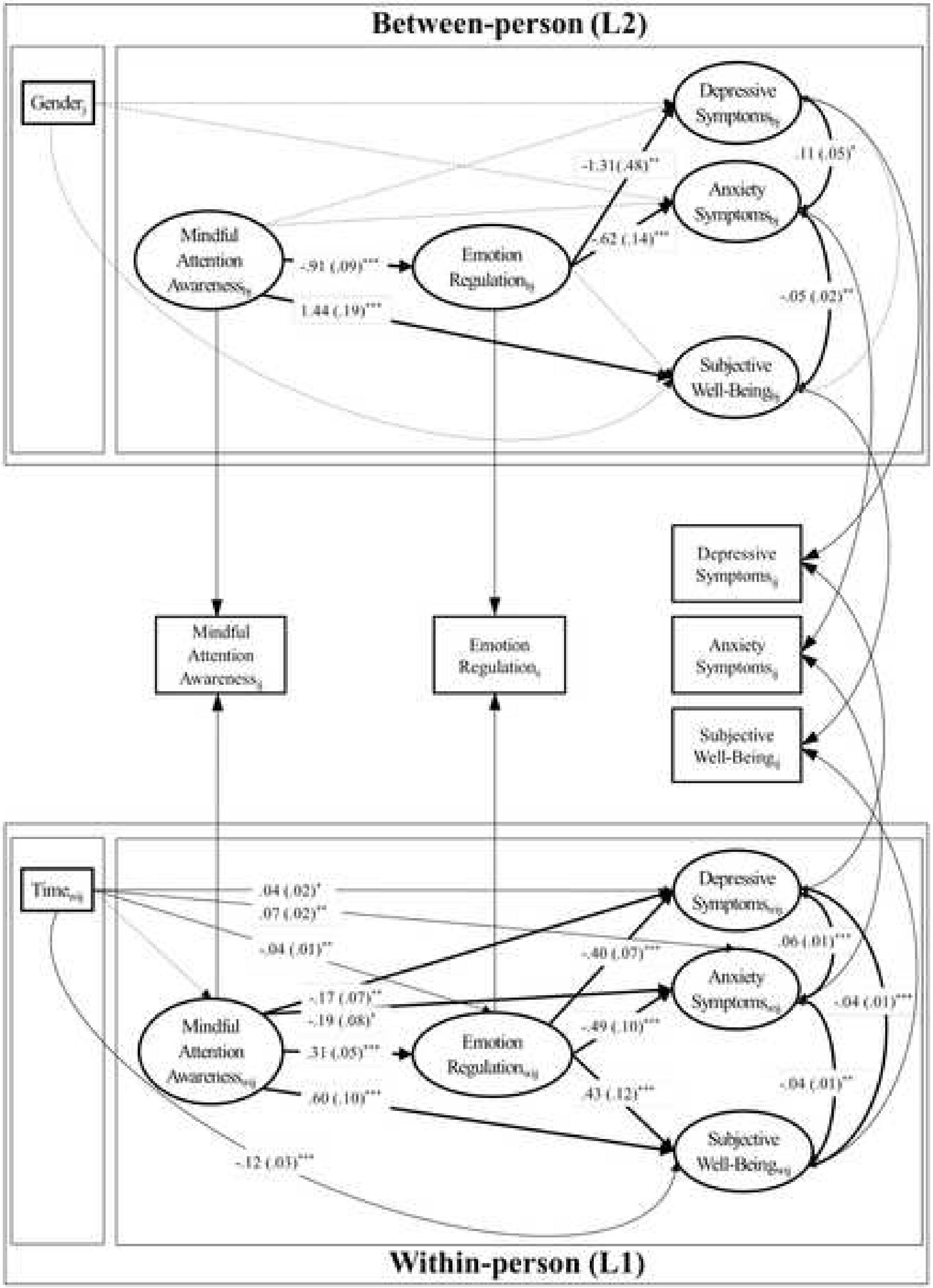

Data associated with this study

Click here to access/download
Supporting Information
data.dat
