# Supplementary material for: Dispositional mindfulness and mental health in Chinese emerging adults: A multilevel model with emotion dysregulation as a mediator
Source: PLoS One. 2020 Nov 19;15(11):e0239575. doi: 10.1371/journal.pone.0239575 (PMC7676716; doi:10.1371/journal.pone.0239575)
Supplement: Attachment — Submitted filename: PONE-D-20-06030_R1 (1).pdf [file pone.0239575.s004.pdf]

PLOS ONE

Dispositional Mindfulness and Mental Health in Chinese Emerging Adults: A Multilevel Model with Emotion Dysregulation as a Mediator

--Manuscript Draft--

**Manuscript Number:**	PONE-D-20-06030R1
**Article Type:**	Research Article
**Full Title:**	Dispositional Mindfulness and Mental Health in Chinese Emerging Adults: A Multilevel Model with Emotion Dysregulation as a Mediator
**Short Title:**	Dispositional Mindfulness and Mental Health
**Corresponding Author:**	Zijun Ke<br>Sun Yat-sen University<br>Guangzhou, Guangdong CHINA
**Keywords:**	dispositional mindfulness, emotion dysregulation, mental health, multilevel modeling, subjective well-being
**Abstract:**	Using a multilevel model, this study examined emotion dysregulation as a mediator between dispositional mindfulness and mental health among Chinese emerging adults. Participants were 191 Chinese emerging adults (female = 172) between 18 and 27 years old (M = 21.06 years, SD = 2.01 years), who completed a questionnaire that assessed their dispositional mindfulness, emotion dysregulation, and mental health outcomes for three times over 12 months, with a three-month lag between each time point. Within-person analysis revealed that emotion dysregulation mediated between dispositional mindfulness and mental health outcomes, including subjective well-being and symptoms of depression and anxiety. Time was positively associated with emotion dysregulation and negatively associated with symptoms of depression and anxiety. Between-person analysis revealed that emotion dysregulation negatively mediated between mindfulness and symptoms of depression and anxiety, but not subjective well-being. These findings call attention to emotion dysregulation as a mediator between dispositional mindfulness and psychological outcomes, particularly of symptoms of depression and anxiety. Notably, the multilevel analysis offered a more nuanced approach to distinguish between within-person vs. between-person processes. Attesting to the relations established in western societies, these findings are applicable to emerging adults in the Chinese context. Evidence was advanced to inform translational research efforts that promote dispositional mindfulness as an asset of mental health.
**Order of Authors:**	Rebecca Y. M. Cheung
	Zijun Ke
	Melody C. Y. Ng
**Response to Reviewers:**	We would like to thank the three reviewers for the thoughtful and constructive comments. Below, we outline the revisions that we have made to address these comments. The revisions have also been highlighted in yellow in the revised manuscript for your convenience.<br><br>Reviewer #1<br>General Comments<br>Comment:<br>Thank you for the interesting manuscript, studying emotion regulation as a mediator between mindfulness and mental health in the Chinese young adult population. I am happy to read that both the within- and between subject factors are taken into consideration in this study design.<br>Our response:<br>We thank Reviewer #1 for the positive comments.<br><br>Comment:<br>A general remark relates to the umbrella term ‘mental health’ used in the research question, this remains rather broad. The introduction includes a description, but

Powered by Editorial Manager® and ProduXion Manager® from Aries Systems Corporation

throughout it is not always clear how mental health is defined with respect to design and outcomes. The separate measures (depression, anxiety, and subjective wellbeing) are sometimes interchanged with the broader mental health concept. Does this then refer to a composite measure of these questionnaires?
Our response:
Thank you for the comment. Our definition is consistent with the definition of health provided by the World Health Organization [3]. Specifically, we refer mental health to better mental well-being and low levels of psychological symptoms.

Comment:
A very high percentage of the study population is female (90%), this is correctly mentioned as a limitation at the end of the manuscript, but briefly. Gender is taken as a covariate in the analyses and conclusions are drawn on these analyses in the beginning of the discussion. This seems to me a big leap and I would advise to be careful with this statement. Reflecting on this earlier in the discussion would help. Another suggestion is to change the number into percentage for easy reading.
Our response:
Thank you for the comment. We have now used percentage and number for easy reading (p. 7). In the Discussion, we have removed, "[g]ender was not related to symptoms of depression and anxiety." We have instead stated (p. 23), "gender was not related to psychological symptoms in the model. However, our small sample of men (i.e., 9.95%) precluded us from drawing meaningful conclusions about gender as a correlate of psychological distress."

Comment:
In the discussion, more attention could be payed to the implications of the findings for the wellbeing of the Chinese population. Some elements of the introduction, methods and results do not resonate in this last section.
Our response:
Thank you for the comment. We have now incorporated existing findings of Chinese samples in the Discussion to make the manuscript more coherent. For example, we have added (p. 22), "[t]he findings resonated with recent findings conducted in the Chinese context, in that emotion dysregulation mediated between dispositional mindfulness and symptoms of depression and anxiety [10]." We have also noted (p. 22), "[t]hese findings contradicted recent cross-sectional studies [29,69] showing the link between Chinese college students' emotion regulation strategies and life satisfaction (i.e., a major component of subjective well-being [70])." By situating our findings to the Chinese context, we hope our readers are better informed.

Introduction
Comment:
The introduction provides clear evidence for the link between mindfulness, emotion regulation and mental health. The importance of considering all three factors is furthermore evident. I did have some difficulties keeping track of the order in which the evidence is discussed. The three concepts are sometimes mixed within a paragraph aiming to focus on one of the concepts. A clearer distinction would aid readers in the process. The rational for examining both within- and between subject findings simultaneously (an important part of this manuscript) could use some elaboration.
Our response:
In the revised introduction, we broadly discussed the role of mindfulness in mental health (p. 3) and then moved onto emotion regulation as a predictor of mental health (p. 3). In the next section, we briefly discussed Teper et al.'s theoretical framework [13] and Garland et al.'s framework [21] concerning the mediating role of emotion regulation (p. 4), which was followed by empirical findings (p. 4-5). To situate the study in the Chinese context, we further discussed the findings in Chinese samples (p. 5), with mindfulness as a predictor of emotion regulation and mental health. To ensure that our audience can fully grasp the meanings of within- vs. between-person effects, we discussed the differences, with examples in the context of mindfulness, emotion regulation, and mental health correlates. Of note, partitioning among within- and between-person associations is important, because otherwise the results could sometimes be biased [34] or uninterpretable [35] (p. 6).
We hope that the flow has now provided a clearer picture in supporting the present study.

	Comment:<br>In the first paragraph, I would avoid using the words adjustment outcomes and change these to mental health outcomes to make the link explicit.<br>Our response:<br>We thank the reviewer for the comment. We have revised the introduction by changing “adjustment outcomes” to “mental health outcomes.”<br><br>Introduction: Section 'processes of mental health in the Chinese context'<br>Comment:<br>Section 'processes of mental health in the Chinese context': This section contains several long sentences with unclear structures.<br>Our response:<br>The long sentences have now been shortened to increase the readability (p. 5).<br><br>Comment:<br>‘similar findings were suggested’ needs to be ‘were found’.<br>Our response:<br>We have now updated the sentence on page 5.<br><br>Comment:<br>I would highlight a study into emotion regulation in this context to balance the section (e.g., take out a mindfulness example and replace it with one for emotion regulation).<br>Our response:<br>Thank you for the comment. We have now highlighted a study (p. 5) showing that changes in emotion regulation strategies were associated with changes in changes in depressive symptoms, life satisfaction, and general health over time [29].<br><br>Comment:<br>‘These findings highlight’ in present tense.<br>Our response:<br>We have revised the introduction by changing “These findings highlighted” to “These findings highlight” on page 5.<br><br>Introduction: Section 'within- vs. between-person effects'<br>Comment:<br>What about emotion regulation in the first sentence?<br>Our response:<br>Thank you for the comment. The first sentence has now been updated to, “[a] majority of research to-date focuses on between-person associations of mindfulness, emotion dysregulation, and mental health.”<br><br>Comment:<br>’Partitioning between within- and between-person’: consider using a synonym (e.g. among).<br>Our response:<br>We have changed “Partitioning between within- and between-person associations” to “Partitioning among within- and between-person associations” on page 6.<br><br>Comment:<br>A clinical interpretation of the importance to distinguish the within-person results and between-person results is lacking. Why is it relevant to separate them, apart from possible biases? What are the differences in interpretation for between-person results and within-person results? Examples are given for within-person results but these are not compared to between-person differences. How can separating both approaches help advance guidelines for future treatment options?<br>Our response:<br>Thank you for the comment. In the revised introduction, we have highlighted the statistical significance for distinguishing among the within-person and between-person findings (p. 5-6), in that “partitioning between among within- and between-person associations is important, because otherwise the results could be biased [34] or uninterpretable [35].”<br>Given that the literature has primarily shown between-person effects (p. 5-6), conclusions could only be drawn, specifically, in that when person i has a higher score in mindfulness than person j, then person i would have a higher score in emotion

regulation and mental health than would person j. In this study, we argued that it is theoretically important to also address whether increases in mindfulness in person i are linked to his or her own better emotion regulation and mental health over time.
The present findings have important implications, as addressed in the Discussion. At the within-person level (p. 21-22), practitioners could tailor their practices and interventions to the progress of each client. For example, to enhance subjective well-being and reduce psychological symptoms, practitioners can enhance within-person improvements in mindfulness and emotion regulation, as mindfulness affects mental health outcomes both directly and through adaptive emotion regulation. At the between-person level (p. 23), practitioners should be made aware of their clients' levels of mindfulness, emotion dysregulation, and mental health relative to other people. For example, returning to Figure 1, in relation to person j, person i's greater level of mindfulness was associated with a lower level of emotion dysregulation and better well-being than person j, but not directly with fewer symptoms of depression and anxiety. Understanding these associations could help practitioners gauge the between-person importance or relevance of mindfulness in various mental health outcomes."

Comment:
The transition between the two paragraphs in this section is not very clear.
Our response:
Under the section 'Within- vs. Between-Person Effects, we have now strengthened the transition by adding the sentence, "[s]everal studies to-date have demonstrated the utility of within-person analyses in mindfulness and well-being."

Introduction: 'This study'
Comment:
Is the analysis technique novel or the used approach?
Our response:
We have deleted "novel" from "This Study" given that it has been used in previous research.

Comment:
Why is this unique? Elaborate in the paragraph above.
Our response:
In the revised manuscript, we have added (p. 6-7), "[a]s stated earlier, previous research predominantly examined between-person effects among mindfulness, emotion regulation, and mental health [5-11]. Although some studies have begun to examine within-person effects [36-38], few, if any, have teased apart within-person from between-person effects."

Comment:
The information 'may be linked to covariates including time and gender' is new information. A motivation for these choices could be added to the introduction.
Our response:
Thank you for the comment. On page 5, we have strengthened our arguments concerned with time by adding, "changes in mindful awareness were associated with subsequent changes of stress response and anxiety symptoms [28]. Similarly, changes in emotion regulation strategies were associated with changes in changes in depressive symptoms, life satisfaction, and general health over time [29]. As such, timing and changes were crucial in linking mindfulness, emotion regulation, and mental health outcomes."
As for gender, previous research conducted with Chinese samples revealed that gender was associated with depression [39], anxiety [40], and subjective well-being [41]. As such, gender was included in this study as a covariate (p. 7).

Method
Comment:
Participants were at 18-27 years old: needs to be between. This information seems part of the result section (same as the retention rate).
Our response:
We have updated "[p]articipants were at 18-27 years old" to "[p]articipants were between 18 and 27 years old." Since age and retention were not the study focus or part of the hypothesis, we have only included the information under "Participants" of the Method section (p. 7-8).

Powered by Editorial Manager® and ProduXion Manager® from Aries Systems Corporation

Comment:
What were the inclusion and exclusion criteria?
Our response:
We have added the following sentence (p.8), "Inclusion criteria included college-enrolled emerging adults who were proficient in Chinese, within the age range between 18 and 29 years old [42], who agreed to participate for three time points over the course of a year."

Comment:
Which number of participants was aimed at?
Our response:
Thank you for the comment. We have conducted a post hoc power analysis based on Monte Carlo simulations [76]. Based on the estimated parameter values from the multilevel mediation analysis, we repeatedly simulated 1000 new data sets and fitted the same multilevel mediation model to these simulated data sets. By counting the proportion of significant results, we obtained the empirical post hoc power. The results showed that on average, the post hoc power for the six indirect effects considered in the study (three at the within-person level and three at the between-person level) was 80.2%, suggesting that it was adequate to aim for 191 participants.

Reference:
76. Gelman A, Hill J. Data analysis using regression and multilevel/hierarchical models. Cambridge: Cambridge University Press; 2006.

Comment:
How many emails were send out?
Our response:
Two mass emails were sent out (p. 7-8).

Comment:
The begin structure is unclear; some information from the procedure part belongs to a part about participants and vice versa.
Our response:
We have worked to ensure that the Participants and Procedures sections were independent from each other (p. 7-8). Specifically, the Participants section involves recruitment method, inclusion criteria, retention rate, and demographics. The Procedures section involves ethics approval information, informed consent details, and data collection procedures.

Comment:
What sort of informed consent was given?
Our response:
We have revised the Procedures section by stating the following (p. 8), "The study was approved by the Human Research Ethics Committee of the first author's university prior to its implementation. All procedures performed were in accordance with the ethical standards of the institutional research committee and the 1964 Helsinki declaration and its later amendments. Informed consent to participate in the present study was obtained prior to the administration of the questionnaire."

Comment:
How much time did participants investigate in the study?
Our response:
We have now included the following information (p. 8), "[e]ach packet took approximately 30 minutes to complete."

Method: Measures
Comment:
This section could benefit from a description of the reliability and validity of the used scales.
Our response:
Based on previous research, we have now included the reliability and validity of the scales used in the present study (p. 8-12).

Powered by Editorial Manager® and ProduXion Manager® from Aries Systems Corporation

Comment:
The timeframes of the mental health questionnaires differ (one assesses the past week, another the past month). Could this have influenced the findings in any way?
Our response:
We agree with Reviewer #1 that the timeframes of the mental health questionnaire differ. As a limitation, we have now stated (p.24), "the timeframes of the mental health questionnaires differ. For example, the PHQ-9 assessed depressive symptoms in the past two weeks, whereas the Mental Health Continuum Short Form assessed emotional, social, and psychological well-being in the past four weeks, whereas the MHC-SF assessed emotional, social, and psychological well-being in the past four weeks. Future research may consider standardizing the timeframes to increase precision of the variables."

Comment:
Anxiety symptoms: was used to measure anxiety, not depression.
Our response:
Thank you for the comment. We have made relevant changes in the revised manuscript (p. 11).

Method: Data analysis
Comment:
The general writing style of this section is suggestive, which results in uncleairity when reading.
Our response:
We have expanded and revised the Data Analysis section. A more affirmative writing style has been used.

Comment:
Why are there no details provided on the multilevel mediation model used here (now upon request)? Was growth curve modelling used? Some information would better fit the introduction.
Our response:
Thank you for the comment. We have now provided a statistical description of the multilevel mediation model (p. 12-15). The multilevel mediation model used in the analysis was not a growth curve model. To avoid confusion, we have removed sentences related to growth curve modeling in the revised manuscript. Two major differences between multilevel mediation model and growth curve model are as follows:
First, in our analysis, the relationship between the independent variables and the mediator (i.e., reflected by the "a" path) and the relationship between the mediator and the dependent variables (i.e., reflected by the "b" paths) were estimated simultaneously. In growth curve modeling with time-varying covariates, however, these relationships were estimated separately. The estimated "a" path and "b" paths could be correlated when data are nonnormal and when robust methods are used (i.e., when MLR is used in Mplus; see the discussion in http://www.statmodel.com/discussion/messages/11/9365.html?1396831771). In our analysis, MLR was used and the possible association between the estimated "a" path and "b" path could be captured. In contrast, the association could not be captured using growth curve modeling. This might have an impact on the inferences on the indirect effect, which was defined as the product of the "a" path and the "b" path (e.g., see [77; p. 92]; and the explanation regarding the difference between the delta method used in Mplus and Sobel test in http://www.statmodel.com/discussion/messages/11/9365.html?1396831771).
Second, we relied on the multilevel SEM approach [34] to separate the within- and between-person components of variables. In contrast, as a typical multilevel modeling, growth curve modeling relies on group centering to fulfill this objective. As discussed in [34], the multilevel approach was relatively more biased.

Reference:
77. MacKinnon DP. Introduction to statistical mediation analysis (Multivariate applications series). Taylor & Francis Group/Lawrence Erlbaum Associates; 2008.

Comment:

	Please specify the used levels.<br>Our response:<br>We have now specified the used levels in greater detail.<br><br>Comment:<br>Separate results from methods.<br>Our response:<br>Thank you for the comment. The results have been separated from methods.<br><br>Comment:<br>The section on the mediation path is difficult to follow.<br>Our response:<br>Thank you for the comment. We have revised the Data Analysis section. Now the logic of this section flows more naturally and it is easier to understand. Specifically, we first described the statistical approach to multilevel mediation used in this study (p. 12). We then explained what covariates and why they were considered in the model (p. 12). Next, we explained why some paths were fixed and some was allowed to be random across participants (p.13). We also described in detail the multilevel mediation model used in the analysis and explained the practical meanings of relevant model parameters (p. 13-15). Finally, we explained why the proportion of indirect effect was not calculated (p. 15).<br><br>Comment:<br>The last part of this section is especially unclear, does this mean that the used analysis require not much faith? Why is this not mentioned in the discussion?<br>Our response:<br>Thank you for the comment. The last sentence of the Data Analysis section (p. 15), "[u]nless the sample size was fairly large, Hayes [60, p.189] recommended not 'having much faith' in this measure" explains the reason why we did not compute the ratio of indirect effect to the total effect. It was not related to the method used in the analysis.<br><br>Results<br>Comment:<br>When reading this section I would first expect general sample information, including cores on all outcome measures. Then correlational information... Before considering correlations, I am curious about how the population scored on the measures. To aid the interpretation, it would be good to mention some reference points for ranges. For example, to interpret the 2.85 score on emotion regulation, I would like to know what this means. What score reflects serious difficulties with emotion regulation?<br>Our response:<br>Thank you for the comment. We have now briefly described the correlational findings at the beginning of the results section (p. 15).<br>As for how the current sample scored on the measures compared to other study samples, we have now included the information under the measures section (p. 8-12). Specifically, compared to other Chinese samples, participants from this study had similar mean levels of dispositional mindfulness [44], emotion dysregulation [46], and symptoms of depression and anxiety [50]. Nevertheless, they had greater mean levels of subjective well-being [56]. This information has also been included in the discussion section (p. 24).<br><br>Comment:<br>Table 1: This table shows many results at the same time. While it seems correct, it might be easier for readers to separate the correlations from the means and standard deviations of the outcomes (make two tables).<br>Our response:<br>We have separated Table 1 into two tables according to Reviewer #1's recommendation. Table 1 shows the means, standard deviations, ranges, ICCs, and design effects, whereas Table 2 shows the zero-order correlations.<br><br>Comment:<br>Gender does not make sense as M and SD.<br>Our response:<br>We have removed the M and SD of gender in Table 1.

Powered by Editorial Manager® and ProduXion Manager® from Aries Systems Corporation

Comment:
The within-person correlations are not part of the analyses section, a clear rational for these results does not follow naturally.
Our response:
Thank you for the comment. We have revised the Data Analysis section (p. 12-15). Now the subsection contains a detailed description on the multilevel mediation model. The analyses regarding the within- and between-person associations have also been described.

Results: 'Multilevel Structural Equation Modeling'
Comment:
Are the design effect results reported somewhere?
Our response:
The design effect results have now been reported in Table 1.

Comment:
Some information in this section would fit the analyses section better.
Our response:
To enhance clarity and organization, we have restructured the Data Analysis and the Results sections (p. 12-20).

Comment:
Results are better presented numerically, an interpretation of the variances explained suites the discussion (interesting information). They are part of figure 1, might be good to mention this earlier in the section.
Our response:
Thank you for the comment. As we were unable to denote the % of variance in Figure 1, we did not describe the figure early on. However, at the end of the paragraph, we have recommended the readers to refer to Figure 1 for specific path coefficients.

Results: 'Within-person indirect Effect of dispositional mindfulness (and between)'
Comment:
How do the results mentioned here relate to the heading title of indirect effects (they mention direct effects)?
Our response:
Thank you for the comment. The section refers to indirect effects.

Comment:
Table 3: Please explain the arrows and the c'w1i notations in the notes.
Our response:
Thank you for the comment. We have added an explanation in note on Table 3 to explain the notations.

Comment:
Table 4: The explanation helps. Some of these elements could be taken into the table, providing a heading for within and between analyses results. Consider keeping the format similar to the other tables (e.g., using * instead of boldfaced numbers for significance).
Our response:
We have revised Table 4 by using * instead of boldfaced numbers for significant numbers.

Discussion
Comment:
The first section clearly summarizes the results. This part could benefit from an integrated interpretation of the findings. For example, what could be the reason for the surprising finding of time?
Our response:
Thank you for the comment. At the beginning of the Discussion, we have included a potential reason for the surprising finding of time (p. 21) by stating, "potentially due to an increasing level of academic stress over the school year." To ensure that the opening paragraph is clear and succinct, we have explained the findings in greater detail in the subsequent paragraphs.

Comment:
The statements ‘findings advance the field by using a process-oriented approach’ and ‘demonstrates the utility of multilevel modeling’ could use more explanation.
Our response:
Thank you for the comment. To ensure that our discussion is clear and informative, the statement, “findings advance the field by using a process-oriented approach,” have been revised to “[t]hese findings advanced the field by establishing within- and between-person relations between dispositional mindfulness and mental health via emotion dysregulation.” (p.21)
The statement, “demonstrates the utility of multilevel modeling” have been revised to “[t]his study used multilevel modeling by partitioning between-person relations from within-person relations.”(p.21)

Comment:
The within-person and between-person definitions (when mindfulness of person i increases etc.) can be left out; this is sufficiently clear from the introduction.
Our response:
We thank Reviewer #1 for the recommendation. We have removed the within-person definition in the discussion. To concretely explain the between-person findings and their clinical significance, however, we have decided to keep the notations (e.g., person i vs person j; p. 22-23).

Comment:
The section on between-person findings with emotion regulation as an explanatory variable is confusing because of the sentence structures.
Our response:
We have now revised the section (p.22-23).

Comment:
Please elaborate on ‘stage-salient variables’.
Our response:
Thank you for the comment. Stage-salient variables refer to variables specific to a certain developmental period, i.e., in our case, emerging adulthood. In the discussion, we have added, “other stage-salient variables in emerging adulthood, such as having increasing responsibility, becoming more financial independent, and having a greater commitment in romantic relationships [73], may be more strongly associated with positive well-being.” (p.23)

Reviewer #2
Comment:
This paper investigates whether emotion regulation is a mediator between mindful awareness and depression, anxiety, and subjective wellbeing in Chinese young adults. Findings suggest that emotion regulation mediates the association between mindful awareness and wellbeing both at the within-person and the between-person level, with the exception that emotion regulation did not mediate the between-person association between mindfulness and subjective wellbeing.
This is an interesting and well-written paper. I only have a few minor comments.
Our response:
We thank Reviewer #2 for the positive comments.

Minor Comments
Comment:
I would prefer the term ‘sum score’ or ‘total score’ rather than ‘composite score’ (p. 14) to reflect the total score on a single questionnaire, given that composite score is usually reserved for a score that reflects a combination of scales.
Our response:
We have revised the method by changing “composite scores” to “mean scores.” (p.8-12)

Comment:
“The item scores were summed to form a composite score of depressive symptoms”. However, Table 1 appears to report mean scores rather than sum scores (on a scale from 0 to 3 instead of the original scale from 1 to 4?).
Our response:

Powered by Editorial Manager® and ProduXion Manager® from Aries Systems Corporation

	Thank you for the comment. We apologize for the confusion. In this study, participants rated on a scale from 0 to 3 for each item of the PHQ-9. Afterwards, the item scores were averaged to form a score. The description has now been updated in the manuscript (p. 10).<br><br>Comment:<br>For subjective wellbeing, unlike the other scales, it is not explicitly stated how the score was calculated (mean score of the 14 items?).<br>Our response:<br>We have now stated how the subjective well-being score was calculated (p. 12), "The item scores were averaged to form a mean score, with higher scores indicating better well-being."<br><br>Comment:<br>In Table 1 the mean for emotion regulation is 2.97 but in the supplementary data set it appears to be 3.97 if I am not mistaken.<br>Our response:<br>Thank you for pointing this out. We have corrected the means and standard deviations for emotion dysregulation.<br><br>Comment:<br>Please explain 'stage-salient variables' (p. 24). Do have some suggestions as to which stage-salient variables may be relevant to include in future research?<br>Our response:<br>Thank you for the comment. Stage-salient variables refer to variables specific to a certain developmental period, i.e., in our case, emerging adulthood. In the discussion, we have added (p.23), "other stage-salient variables in emerging adulthood, such as having increasing responsibility, becoming more financial independent, and having a greater commitment in romantic relationships [73], may be more strongly associated with positive well-being."<br><br>6. The manuscript contains a few typos:<br>- p. 10 stwell-being<br>- p. 14 "how often did you feel that that you had …."<br>- p. 14 "A sample items included" "<br>- p. 14 The 7-item Generalized Anxiety Disorder-7 … was used to measure depressive symptoms". Depressive symptoms should be anxiety symptoms.<br>- p. 13 "The 15-item Mindful Attention Awareness Scale …. on a 6-point scale from 1 (almost always) to 5 (almost never)". This should say 'a scale from 1 to 6'?<br>Our response:<br>Thank you for the comment. The typos have been removed in the revised manuscript.<br><br>Reviewer #3:<br>Title Page<br>Comment:<br>Title arose some expectations that were not accomplished later.<br>Our response:<br>Thank you for the comment. We have updated the title to, "Dispositional Mindfulness and Mental Health in Chinese Emerging Adults: A Multilevel Model with Emotion Dysregulation as a Mediator."<br><br>Comment:<br>DERS: Difficulties in ER Scale measures emotional dysregulation rather than emotion regulation. Dysregulation: A failure to regulate properly.<br>Our response:<br>We agree with Reviewer #3's comment. In our original manuscript, the scores of the negative items of DERS were reversed, such that the final scores reflected a greater ability in emotion regulation.<br>Based on Reviewer #3's comment, we have unreversed the scores, such that higher scores reflected greater emotion dysregulation. Where appropriate, we have now used "emotion dysregulation" in the revised manuscript.<br><br>Comment:<br>Many authors have pointed out that these mindful scales are not measuring mindful

Powered by Editorial Manager® and ProduXion Manager® from Aries Systems Corporation

activities, they assess dispositional mindful activities.
Our response:
We agree with Reviewer #3's comment and have changed our wordings to "dispositional mindfulness" in describing the variable under study.

Abstract
Comment:
Provide here M and SD for participants' age.
Our response:
We have revised the abstract by adding M and SD of the participants' age.

Comment:
A questionnaire about what?
Our response:
We have added that the questionnaire assessed their dispositional mindfulness, emotion dysregulation, and mental health outcomes.

Introduction
Comment:
Avoid using regulating emotions in the definition of emotion regulation.
Our response:
We have revised the revised the sentence to the following, "[e]motion regulation is defined as the process in modulating emotions and emotional responses [14-15]." (p.3)

Introduction: Within- vs. Between-Person Effects
Comment:
Agree but this section above is a little bit tangential to your research. You did not implement a RCT MBI.
Our response:
We apologize about the confusion and agree with Reviewer #3 that we did not implement a mindfulness-based intervention. Given that very few, if any, studies assessed within- vs. between-person effects of dispositional mindfulness on mental health, we have reviewed mindfulness-based intervention studies using a multilevel modeling approach.

Method
Comment:
Does this Committee a code?
Our response:
We have revised the Method by stating (p. 8), "The study was approved by the Human Research Ethics Committee of the first author's university institution prior to its implementation. All procedures performed were in accordance with the ethical standards of the institutional research committee and the 1964 Helsinki declaration and its later amendments."

Comment:
The sample is not enough, gender was not balanced, there was no intervention…
Our response:
We agree with Reviewer #3 that the sample size could have been larger and gender could be more balanced.
In terms of sample size, we have conducted a post hoc power analysis based on Monte Carlo simulations [76]. Based on the estimated parameter values from the multilevel mediation analysis, we repeatedly simulated 1000 new data sets and fitted the same multilevel mediation model to these simulated data sets. By counting the proportion of significant results, we obtained the empirical post hoc power. The results showed that on average, the post hoc power for the six indirect effects considered in the study (three at the within-person level and three at the between-person level) was 80.2%, suggesting that it was adequate to aim for 191 participants.
As for gender, we have stated in the Discussion (p. 23), "our small sample of men (i.e., 9.95%) precluded us from drawing meaningful conclusions about gender as a correlate of psychological distress." We have also acknowledged, (p. 24), "our participants were mainly female. Future studies with gender-balanced samples are necessary to draw meaningful conclusions for the effects of gender."
We understand that intervention studies are necessary to improve mental health.

	However, it is beyond the scope of the present study. As a future direction, we have added (p. 24), "researchers should translate the present findings and design targeted interventions to improve mental health outcomes."<br>Reference:<br>76. Gelman A, Hill J. Data analysis using regression and multilevel/hierarchical models. Cambridge: Cambridge University Press; 2006.<br><br>Comment:<br>This tool measures dispositional mindful awareness.<br>Our response:<br>We have now updated the description to the following (p.8), "[t]he 12-item Cognitive and Affective Mindfulness Scale – Revised (CAMS-R) [43] was used to assess dispositional mindfulness on a 4-point scale from 1 (rarely/ not at all) to 4 (almost always)." The measure, CAMS-R, broadly assessed dispositional mindfulness, in addition to dispositional mindful awareness.<br><br>Comment:<br>Cronbach's alpha of subscales should be also reported. You can use intercorrelations at T1, T2 and T3 as well.<br>Our response:<br>Thank you for the comment. We have now indicated the Cronbach's alpha of subscales for the Cognitive and Affective Mindfulness Scale – Revised (p. 9), the Difficulties in Emotional Regulation Scale (p. 9), and the Mental Health Continuum Short Form (p. 12). The intercorrelations between T1, T2 and T3 have been reported for all measures as well (p. 8-12).<br><br>Comment:<br>Is there a cut-off in the scoring to indicate depression?<br>Our response:<br>Thank you for the question. The cut-off scores for the Patient Health Questionnaire-9 and the Generalized Anxiety Disorder-7 measures have now been reported on page 10.<br><br>Results<br>Comment:<br>Gender: 0 male and 1 female?<br>Our response:<br>We have now denoted "0=male; 1=female" on Tables 1-3.<br><br>Comment:<br>Why are the correlations of mindfulness moderate / low at T1, T2, T3?<br>Our response:<br>The correlations of dispositional mindfulness between T1 and T3 were .53 - .58 over time, ps < .001, suggesting moderate effect sizes [62]. Even though the scale was intended to measure stable and dispositional mindfulness, our data suggest potential changes over time.<br><br>Comment:<br>Based on the correlations, gender could be a moderator.<br>Our response:<br>We agree with Reviewer #3's comment. However, our small sample of men (i.e., 9.95%) precluded us from drawing meaningful conclusions about gender as a moderator. As a limitation, we have stated (p. 24), "our participants were mainly female. Future studies with gender-balanced samples are necessary to draw meaningful conclusions for the effects of gender."<br><br>Comment:<br>M and SD seem to be averaged, this should be notified in the table and put in the first column the range of every single instrument.<br>Our response:<br>We have now stated (p. 15), "Table 1 summarizes the means, SDs, ranges, ICCs, and design effects."<br><br>Comment:

Powered by Editorial Manager® and ProduXion Manager® from Aries Systems Corporation

	ICC is more appropriate to assess the level of agreement among raters. Where is reported the ICC, Table 1 is not a ICC.<br>Our response:<br>Thank you for the comment. We have added ICCs in Table 1 for all the variables under study.<br><br>Discussion<br>Comment:<br>You did not implement an intervention... The discussion needs to follow a tentative argument… The undergraduate sample is too biased for the conclusions (should be tentative rather than so affirmative). Discussion can be more tentative than affirmative due to data did not allow those conclusions.<br>Our response:<br>Thank you for the comment. We agree with Reviewer #3 that the present study involved no experiments and interventions. Thus, in the discussion, we have avoided wordings such as “causes”, “affects”, or “influences.” Based on the present findings, tentative arguments have been made, without biases or exaggerations. As a limitation, we have added, “researchers should translate the present findings and design targeted interventions to improve mental health outcomes.”
**Additional Information:**	
**Question**	**Response**
**Financial Disclosure**<br><br>Enter a financial disclosure statement that describes the sources of funding for the work included in this submission. Review the submission guidelines for detailed requirements. View published research articles from *PLOS ONE* for specific examples.<br><br>This statement is required for submission and **will appear in the published article** if the submission is accepted. Please make sure it is accurate.	This study was funded by two Internal Grants from The Education University of Hong Kong awarded to RYMC (RG 60/2014-2015R and 04190).

**Unfunded studies**<br>Enter: *The author(s) received no specific funding for this work.*<br><br>**Funded studies**<br>Enter a statement with the following details:<br>• Initials of the authors who received each award<br>• Grant numbers awarded to each author<br>• The full name of each funder<br>• URL of each funder website<br>• Did the sponsors or funders play any role in the study design, data collection and analysis, decision to publish, or preparation of the manuscript?<br>• **NO** - Include this sentence at the end of your statement: *The funders had no role in study design, data collection and analysis, decision to publish, or preparation of the manuscript.*<br>• **YES** - Specify the role(s) played.<br><br>* typeset	
**Competing Interests**<br><br>Use the instructions below to enter a competing interest statement for this submission. On behalf of all authors, disclose any competing interests that could be perceived to bias this work—acknowledging all financial support and any other relevant financial or non-financial competing interests.<br><br>This statement **will appear in the published article** if the submission is accepted. Please make sure it is accurate. View published research articles from *PLOS ONE* for specific examples.	The authors have declared that no competing interests exist.

Powered by Editorial Manager® and ProduXion Manager® from Aries Systems Corporation

**NO authors have competing interests**<br><br>Enter: *The authors have declared that no competing interests exist.*<br><br>**Authors with competing interests**<br><br>Enter competing interest details beginning with this statement:<br><br>*I have read the journal's policy and the authors of this manuscript have the following competing interests: [insert competing interests here]*<br><br>* typeset	
**Ethics Statement**<br><br>Enter an ethics statement for this submission. This statement is required if the study involved:<br><br>• Human participants<br>• Human specimens or tissue<br>• Vertebrate animals or cephalopods<br>• Vertebrate embryos or tissues<br>• Field research<br><br>Write "N/A" if the submission does not require an ethics statement.<br><br>General guidance is provided below. Consult the submission guidelines for detailed instructions. **Make sure that all information entered here is included in the Methods section of the manuscript.**	Institutional ethics committee that approved the study: Human Research Ethics Committee, The Education University of Hong Kong (Approval #: 2015-2016-0352). A written consent had been obtained from each participant prior to the beginning of the study.

Powered by Editorial Manager® and ProduXion Manager® from Aries Systems Corporation

**Format for specific study types**<br><br>**Human Subject Research (involving human participants and/or tissue)**<br>• Give the name of the institutional review board or ethics committee that approved the study<br>• Include the approval number and/or a statement indicating approval of this research<br>• Indicate the form of consent obtained (written/oral) or the reason that consent was not obtained (e.g. the data were analyzed anonymously)<br><br>**Animal Research (involving vertebrate animals, embryos or tissues)**<br>• Provide the name of the Institutional Animal Care and Use Committee (IACUC) or other relevant ethics board that reviewed the study protocol, and indicate whether they approved this research or granted a formal waiver of ethical approval<br>• Include an approval number if one was obtained<br>• If the study involved *non-human primates*, add *additional details* about animal welfare and steps taken to ameliorate suffering<br>• If anesthesia, euthanasia, or any kind of animal sacrifice is part of the study, include briefly which substances and/or methods were applied<br><br>**Field Research**<br><br>Include the following details if this study involves the collection of plant, animal, or other materials from a natural setting:<br>• Field permit number<br>• Name of the institution or relevant body that granted permission	
**Data Availability**<br><br>Authors are required to make all data underlying the findings described fully available, without restriction, and from the time of publication. PLOS allows rare exceptions to address legal and ethical concerns. See the PLOS Data Policy and FAQ for detailed information.	Yes - all data are fully available without restriction

A Data Availability Statement describing where the data can be found is required at submission. Your answers to this question constitute the Data Availability Statement and **will be published in the article**, if accepted.<br><br>**Important:** Stating 'data available on request from the author' is not sufficient. If your data are only available upon request, select 'No' for the first question and explain your exceptional situation in the text box.<br><br>Do the authors confirm that all data underlying the findings described in their manuscript are fully available without restriction?	
**Describe where the data may be found in full sentences. If you are copying our sample text, replace any instances of XXX with the appropriate details.**<br><br>• If the data are **held or will be held in a public repository**, include URLs, accession numbers or DOIs. If this information will only be available after acceptance, indicate this by ticking the box below. For example: *All XXX files are available from the XXX database (accession number(s) XXX, XXX.)*.<br>• If the data are all contained **within the manuscript and/or Supporting Information files**, enter the following: *All relevant data are within the manuscript and its Supporting Information files.*<br>• If neither of these applies but you are able to provide **details of access elsewhere**, with or without limitations, please do so. For example:<br><br>*Data cannot be shared publicly because of [XXX]. Data are available from the XXX Institutional Data Access / Ethics Committee (contact via XXX) for researchers who meet the criteria for access to confidential data.*<br><br>*The data underlying the results presented in the study are available from (include the name of the third party*	All relevant data are within the manuscript and its Supporting Information files.

Powered by Editorial Manager® and ProduXion Manager® from Aries Systems Corporation

and contact information or URL).<br>• This text is appropriate if the data are owned by a third party and authors do not have permission to share the data.<br><br>* typeset	
Additional data availability information:	

Powered by Editorial Manager® and ProduXion Manager® from Aries Systems Corporation

Manuscript

Click here to access/download;Manuscript;Manuscript 15-7-2020 NO TRACK CHANGES.docx

Dispositional Mindfulness and Mental Health in Chinese Emerging Adults: A Multilevel Model with Emotion Dysregulation as a Mediator

Rebecca Y. M. Cheung[1], Zijun Ke[2*], Melody C. Y. Ng[3]

[1] Department of Early Childhood Education, Centre for Child and Family Studies, and Centre for Psychosocial Health, The Education University of Hong Kong, Hong Kong SAR, China

[2] Department of Psychology, Sun Yat-Sen University, Guangdong, China

[3] Department of Psychology, The Chinese University of Hong Kong, Hong Kong SAR, China

[*] Corresponding author

Email (Zijun Ke): keziyun@mail.sysu.edu.cn

Abstract

Using a multilevel model, this study examined emotion dysregulation as a mediator between dispositional mindfulness and mental health among Chinese emerging adults. Participants were 191 Chinese emerging adults (female = 172) between 18 and 27 years old (*M* = 21.06 years, *SD* = 2.01 years), who completed a questionnaire that assessed their dispositional mindfulness, emotion dysregulation, and mental health outcomes for three times over 12 months, with a three-month lag between each time point. Within-person analysis revealed that emotion dysregulation mediated between dispositional mindfulness and mental health outcomes, including subjective well-being and symptoms of depression and anxiety. Time was positively associated with emotion dysregulation and negatively associated with symptoms of depression and anxiety. Between-person analysis revealed that emotion dysregulation negatively mediated between mindfulness and symptoms of depression and anxiety, but not subjective well-being. These findings call attention to emotion dysregulation as a mediator between dispositional mindfulness and psychological outcomes, particularly of symptoms of depression and anxiety. Notably, the multilevel analysis offered a more nuanced approach to distinguish between within-person vs. between-person processes. Attesting to the relations established in western societies, these findings are applicable to emerging adults in the Chinese context. Evidence was advanced to inform translational research efforts that promote dispositional mindfulness as an asset of mental health.

Keywords: dispositional mindfulness, emotion dysregulation, mental health, multilevel modeling, subjective well-being

Introduction

Mindfulness is a mental state whereby individuals attend to their cognitive, physical, and emotional experience in the present moment nonjudgmentally [1-2]. Previous research suggested that mindfulness is associated with mental health outcomes, which encompass both a low level of psychological symptoms but also better mental well-being [3]. Notably, mindfulness is associated with outcomes including better subjective well-being, and fewer depressive and anxiety symptoms [4-6]. Moving beyond the simple association between mindfulness and mental health, recent studies have identified several mediating psychological mechanisms, including greater self-esteem [7], more positive affect, hope, and optimism [8], lower rumination [9], and better emotion regulation [10-11], thereby suggesting possible chains of processes between mindfulness and mental health. Of note, emotion regulation has received much theoretical and empirical attention as a mediating mechanism [12-13].

Emotion regulation is defined as a process in modulating emotions and emotional responses [14-15]. According to Gratz and Roemer [16], adaptive emotion regulation involves such aspects as acceptance of emotional responses, engagement in goal directed behavior, maintenance of emotional clarity and awareness, and access to adaptive emotion regulation strategies, such as cognitive reappraisal and savoring ability. On the contrary, emotion dysregulation involves difficulties in acquiring regulatory skills or a frequent use of maladaptive strategies, such as rumination, avoidance, and expressive suppression [17-18]. Previous research suggested that our capability to adaptively regulate emotions is central to subjective well-being [18]. Emotion dysregulation not only undermines well-being, but also gives rise to clinical depression, anxiety, psychological distress, and elevated depressive and anxiety symptoms [10, 17, 19-21].

Theoretical Tenets of Emotion Regulation as a Mediator between Mindfulness and Mental Health Outcomes

As a proximal correlate of mental well-being, people's capability to regulate emotions is associated with mindfulness [6, 22-23]. More specifically, Teper et al. [13] theorized that mindfulness is linked to emotion regulation through heightened metacognitive awareness, non-judgmental acceptance, and executive control, all of which are crucial to well-being. Likewise, in mindfulness-to-meaning theory, Garland et al. [12] postulated that mindfulness promotes purpose in life by virtue of greater metacognitive awareness, broadened attention to context, adaptive emotion regulation, ability to savor hedonic experience, and prosocial actions. Both theoretical frameworks suggest close connections between mindfulness, emotion regulation, and psychological well-being.

Supporting the frameworks, cross-sectional studies revealed that emotion regulation mediated between dispositional mindfulness and lower levels of depression and anxiety [11, 23-25], as well as better life satisfaction [26]. Findings based on a clinically depressed and anxious sample suggested that emotion regulation strategies, including cognitive reappraisal and rumination, mediated between dispositional mindfulness and symptoms of depression and anxiety [25]. These findings resonated with research conducted with non-clinical emerging adult samples, in that emotion regulation mediated the inverse relation between dispositional mindfulness and depressive symptoms, in that dispositional mindfulness was related to better emotion regulation and fewer depressive symptoms [23]. In a similar vein, Coffey and Hartman [11] and Coffey et al. [24] found that emerging adults' regulation of negative emotions, impulse control, and goal engagement mediated between dispositional mindfulness and well-being. Altogether, evidence to-date converged to indicate emotion regulation as a vital process between

dispositional mindfulness and well-being.

Processes of Mental Health in the Chinese Context

Among the handful of studies conducted in the Chinese context, dispositional mindfulness predicted better impulse control, better emotion regulation, less procrastination, and fewer psychological distress [10, 27]. Moreover, changes in mindful awareness were associated with subsequent changes of stress response and anxiety symptoms [28]. Similarly, changes in emotion regulation strategies were associated with changes in changes in depressive symptoms, life satisfaction, and general health over time [29]. As such, timing and changes were crucial in linking mindfulness, emotion regulation, and mental health outcomes. Beyond dispositional mindfulness, mindfulness training also increased Chinese adolescents' and adults' well-being and psychological adjustment in Hong Kong [30-31]. In a randomized controlled trial involving a Chinese sample with clinical anxiety, Wong et al. [32] revealed that participants who took an 8-week mindfulness-based cognitive therapy had an increased dispositional mindfulness and a reduced anxiety. Such an increase was found to be more significantly than did participants who received psycho-education based on cognitive behavioral therapy principles. Similar findings were reported in another randomized controlled trial involving a Chinese sample with recurrent symptoms of depression and anxiety [33]. In this study, participants who received an 8-week compassion–mindfulness therapy showed more mental health improvements than did participants from the waitlist control group. These findings highlight that the mechanisms associated with mindfulness are integral to mental health in the Chinese context.

Within- vs. Between-Person Effects

A majority of research to-date, as reviewed above, focuses on between-person associations of mindfulness, emotion regulation or dysregulation, and mental health [e.g., 5-11].

Only a handful of studies partitioned within-person from between-person processes. Between- and within-person effects are conceptually and statistically independent. Within-person effect aids the understanding of *intraindividual* processes underlying well-being. For instance, when mindfulness of person *i* increases, then his or her own mental health is expected to also increase over time. Between-person effect accounts for *interindividual* processes underlying well-being. That is, when person *i* has a higher score in mindfulness than person *j*, then person *i* is expected to have a higher score in mental health than is person *j*. Of note, partitioning among within- and between-person associations is important, because otherwise the results could sometimes be biased [34] or uninterpretable [35].

Several studies to-date have demonstrated the utility of within-person analyses in mindfulness and well-being. For example, Galla [36] indicated that a five-day mindfulness training was associated with significant within-person reductions in rumination and depressive symptoms, as well as increases in life satisfaction in healthy adolescents. In addition, many of the improvements were maintained at the three-month follow-up assessment. In another study, an 8-week mindfulness training gave rise to an inverse within-person association between mindfulness and relationship stress [37]. Apart from stress, psychological symptoms, and subjective well-being, within-person associations were also found between dispositional mindfulness and relationship satisfaction [38]. By investigating within-person effects, these studies methodologically precluded confounding between-person factors from the within-person associations between mindfulness and other psychological processes.

This Study

Aside from the studies described in the previous section, past research predominantly examined between-person effects among mindfulness, emotion regulation, and mental health [5-

11]. Although some studies have begun to examine within-person effects [36-38], few, if any, have teased apart within-person from between-person effects. Although within- and between-person findings converged to suggest the mental health benefits of mindfulness, it remains unclear as to whether its clinical significance on emotion regulation and various mental health outcomes differ.

The present study aims to investigate longitudinal within- vs between-person mediating effect between dispositional mindfulness and mental health outcomes via emotion dysregulation. Through this analytic technique, the intraindividual processes and the interindividual processes could be identified. Based on the literature [23-25], we hypothesized that greater dispositional mindfulness would be related to lower emotion dysregulation. Lower emotion dysregulation would, then, be related to fewer symptoms of depression and anxiety, as well as greater subjective well-being. We further hypothesized that the relation between dispositional mindfulness and mental health outcomes would be mediated by emotion dysregulation at both within- and between-person levels. The strength of associations may vary as a function of the level. As stated earlier, time was an important measure in assessing changes within and between variables [28-29]. As such, time was included as a within-person covariate to control for dispositional mindfulness, emotion dysregulation, and mental health outcomes. Based on previous research showing the links between gender and depressive symptoms [39], anxiety symptoms [40], and subjective well-being [41], gender was included as a between-person covariate for mental health outcomes.

Method

Participants

Participants were 191 Chinese emerging adults (female = 172; 90.05%) recruited online

through two mass emails at a major public university in Hong Kong. Inclusion criteria included college-enrolled emerging adults who were proficient in Chinese, within the age range between 18 and 29 years old [42], who agreed to participate for three time points over the course of a year. Participants were between 18 and 27 years old, with a mean age of 21.06 years at Time 1 (*SD* = 2.01 years). As for the retention rate, 93.72% (*n* = 179) of the participants from Time 1 (T1) were retained at Time 2 (T2); 94.41% (*n* = 169) of the participants from T2 were retained at Time 3 (T3).

Procedures

The study was approved by the Human Research Ethics Committee, The Education University of Hong Kong (Approval #: 2015-2016-0352), prior to its implementation. All procedures performed were in accordance with the ethical standards of the institutional research committee and the 1964 Helsinki declaration and its later amendments. A written consent had been obtained from each participant prior to the beginning of the study. At the first time point, participants completed baseline measures. They were then invited to complete follow-up questionnaires twice, with a three-month lag between time points, over the course of a year. Each packet took approximately 30 minutes to complete. Upon completion, participants received a supermarket coupon at each time point (totaling HK$200, i.e., ~US$25.71).

Measures

Dispositional Mindfulness. The 12-item Cognitive and Affective Mindfulness Scale – Revised (CAMS-R) [43] was used to assess dispositional mindfulness on a 4-point scale from 1 (*rarely/ not at all*) to 4 (*almost always*). The CAMS-R assessed three dimensions of mindfulness including awareness, acceptance, and attention. A sample item included, “I can usually describe how I feel at the moment in considerable detail.” The item scores were

averaged, such that higher scores indicated a greater level of mindfulness. The measure was validated previously in a Chinese college sample with adequate convergent validity, reliability, and factor structure [44]. Specifically, participants from this study had a similar mean level of dispositional mindfulness (see Table 1). In this study, the test-retest correlations of the measure between T1, T2, and T3 were moderate at .53 - .58, *p*s < .001. Cronbach's alpha of the measure = .91 - .94 between T1 and T3. More specifically, between T1 and T3, Cronbach's alpha of the awareness subscale = .63 - .80, the acceptance subscale = .62 - .79, and the attention subscale = .77 - .81.

Emotion Dysregulation. The 36-item Difficulties in Emotional Regulation Scale (DERS) [16] was used to assess emotional regulation on a 5-point scale from 1 (*almost never*) to 5 (*almost always*). The measure included 6 subscales including non-acceptance of emotions, difficulties in engaging in goal-directed behavior, impulse control difficulties, lack of emotional awareness, limited access to emotion regulation strategies, and lack of emotional clarity. Sample items included, "I am clear about my feelings" and "When I'm upset, I believe that my feelings are valid and important." The final scores were then averaged, such that higher scores indicated a greater emotion dysregulation. The measure had been validated in a Chinese sample [45]. Compared to another Chinese adult sample [46], participants from this study had a similar mean level of emotion dysregulation (see Table 1). In this study, the test-retest correlations of the measure between T1, T2, and T3 were .70 - .76, *p*s < .001. Cronbach's alpha of the measure = .83 - .87 between T1 and T3. More specifically, between T1 and T3, Cronbach's alpha of the non-acceptance of emotions subscale = .83 - .87, the difficulties in engaging in goal-directed behavior subscale = .56 - .75, the impulse control difficulties subscale = .77 - .85, the lack of emotional awareness subscale = .72 - .75, the limited access to emotion regulation strategies

subscales = .83 - .87, and the lack of emotional clarity subscale = .74 - .77.

Table 1. Means and standard deviations of the study variables (*N* = 191)

Descriptive measures of the study, not just M and SD

Variable	*M*	*SD*	Range	ICC	Design Effect
(1) Gender (0=male; 1=female)	-	-			
**Time 1**					
(2) Dispositional mindfulness	2.85	.43	2.20		
(3) Emotion dysregulation	2.03	.49	2.50		
(4) Depressive symptoms	.61	.50	2.67		
(5) Anxiety symptoms	.61	.58	3.00		
(6) Subjective well-being	4.49	.86	4.21		
**Time 2**					
(7) Dispositional mindfulness	2.80	.48	2.40		
(8) Emotion dysregulation	2.12	.51	2.30		
(9) Depressive symptoms	.68	.56	3.00		
(10) Anxiety symptoms	.65	.64	3.00		
(11) Subjective well-being	4.28	.86	4.50		
**Time 3**					
(12) Dispositional mindfulness	2.78	.52	2.80		
(13) Emotion dysregulation	2.12	.54	2.77		
(14) Depressive symptoms	.74	.61	2.89		
(15) Anxiety symptoms	.81	.68	3.00		
(16) Subjective well-being	4.15	.92	4.86		
**Across Time Points**					
Dispositional mindfulness	2.81	.48		.56	2.01
Emotion dysregulation	2.09	.51		.73	2.31
Depressive symptoms	.68	.56		.63	2.13
Anxiety symptoms	.68	.63		.52	1.93
Subjective well-being	4.32	.89		.64	2.14

* p< 0.05
** p <0.01
Add "*" to describe ICC significance.
Include notes in the bottom of the table to explain what mean ICC and "*".
Range: I assumed that it was the difference between lower and higher score in every single subscale. Add info in the notes.
Add info regarding how you calculated Design effect

Depressive Symptoms. The 9-item Patient Health Questionnaire-9 (PHQ-9) [47] was used to measure depressive symptoms in the past two weeks on a 4-point scale from 0 (*not at all*) to 3 (*nearly every day*). A sample item included, “Feeling bad about yourself - or that you are a failure or have let yourself or your family down?” The item scores were averaged to form a score of depressive symptoms, with higher scores indicating more symptoms. The measure has previously been validated in a Chinese community sample and demonstrated an adequate factor structure, construct validity, internal consistency, and test-retest reliability [48]. Based on a cutoff score of 11 for the detection of depressive disorders [49], upon rescaling, our participants’

summed scores of 5.44 - 6.67 between T1 and T3 were below the clinical cutoff. Compared to another Chinese college sample [50] participants from this study had a comparable level of depressive symptoms (see Table 1). However, a total of 20, 21, and 28 participants at T1, T2, and T3, respectively, reported a summed score of above 11. In this study, the test-retest correlations of the measure between T1, T2, and T3 were .62 - .67, *p*s < .001. Cronbach's alpha of the measure = .87 - .91 between T1 and T3.

Anxiety Symptoms. The 7-item Generalized Anxiety Disorder-7 measure (GAD-7) [51] was used to measure anxiety symptoms in the past two weeks on a 4-point scale from 0 (*not at all*) to 3 (*nearly every day*). A sample item included, "Feeling nervous, anxious or on edge." The item scores were averaged to form a score of anxiety symptoms, with higher scores indicating more symptoms. The measure has been validated in a Chinese sample of hospital outpatients [52] and a sample of Chinese individuals with epilepsy [53]. Based on a cutoff score of 10 for the detection of generalized anxiety disorder [51], upon rescaling our participants' summed scores between 4.22 and 5.67 between T1 and T3 were below the clinical cutoff. Compared to another Chinese college sample [50] participants from this study reported a similar level of anxiety symptoms (see Table 1). However, a total of 14, 20, and 26 participants at T1, T2, and T3, respectively, reported a total score of above 10. In this study, the test-retest correlations of the measure between T1, T2, and T3 were .52 - .56, *p*s < .001. Cronbach's alpha of the measure = 93 - .95 between T1 and T3.

Subjective Well-being. The 14-item Mental Health Continuum Short Form (MHC-SF) [54] was used as a measure for well-being over the past four weeks. A 6-point scale ranging from 0 (*never*) to 5 (*every day*) was used, with sample items including, "how often did you feel satisfied with life" (*emotional well-being*), "how often did you feel that that you had something

important to contribute to society (*social well-being*) and "how often did you feel that your life has a sense of direction or meaning to it" (*psychological well-being*). The item scores were averaged to form a mean score, with higher scores indicating better well-being. MHC-SF had been previously validated in a Chinese adolescent sample, and yielded good validity and reliability [55]. Compared to another Chinese college sample [56], participants from this study had a greater mean level of subjective well-being (see Table 1). In this study, the test-retest correlations of the measure between T1, T2, and T3 were .59 - .69, *p*s < .001. Cronbach's alpha of the measure = .79 - .94 between T1 and T3. More specifically, between T1 and T3, Cronbach's alpha of the emotional well-being subscale = .90 - .94, the social well-being subscale = .79 - .86, and the psychological well-being subscale = .92 - .93.

Data Analysis

Multilevel mediation analyses were conducted using Mplus 8.0 [57] to study the mediating effect of emotion dysregulation on the relationship between ~~mindfulness~~ dispositional mindfulness and mental health outcomes, including depressive symptoms, anxiety symptoms, and subjective well-being, at both the within- and between-person levels. Multilevel modeling has been used in previous research for panel data (e.g., [58]), with a minimum of three waves of observations to partition between within- and between-person associations, which, as introduced previously, have distinctive practical implications. Compared to the traditional multilevel modeling approach to multilevel mediation, the multilevel structural equation modeling (MSEM) approach has the advantage of having a smaller bias.

In this study, we relied on the MSEM approach. Covariates at both levels were considered. At the within-person level, a time variable was included to control for the potential change over time [59]. At the between-person level, gender was included to control for possible

gender differences in symptoms of anxiety and depression.

Regarding whether random slopes should be considered, with only three measurement occasions at level 1, the model allowing all mediation paths (i.e., “a” path from mindfulness to emotion dysregulation, “b” paths from mindful awareness to mental health, and “c” paths from emotion dysregulation to mental health) and the slope of time to be random could not be identified. Therefore, we tested possible random mediation paths sequentially. Results showed that none of the “a”, “b”, and “c′” paths showed substantial individual differences, except for the “c′” path for depression. Additionally, we tested possible random slopes of time (change between two consecutive measurement occasions) and found no significant individual differences. Consequently, all mediation paths and slopes of time were fixed to be invariant across participants, except for the “c′” path for depression in the subsequent analyses.

In sum, the multilevel mediation model under consideration was as follows. The independent variable, the mediator, and the three dependent variables were partitioned into two latent parts:

$$\text{DispositionalMindfulness}_{\text{it}} = \eta_{Mindfulness,it} + \eta_{Mindfulness,i}$$

$$\text{EmotionDysregulation}_{\text{it}} = \eta_{EmotionDysregulation,it} + \eta_{EmotionDysregulation,i}$$

$$\text{Depression}_{\text{it}} = \eta_{Depression,it} + \eta_{Depression,i}$$

$$\text{Anxiety}_{\text{it}} = \eta_{Anxiety,it} + \eta_{Anxity,i}$$

$$\text{Wellbeing}_{\text{it}} = \eta_{Wellbeing,it} + \eta_{Wellbeing,i}$$

More specifically, the level-1 model defined the within-person mediation model. Between the independent variable and the mediator (the fixed “a” path), the equation was:

$$\eta_{EmotionDysregulation,it} = a_w \eta_{Mindfulness,it} + g_M time_{it} + e_{Mit}.$$

From the mediator to the dependent variables (the fixed “b” paths), the equations were:

$$\eta_{Depression,it} = b_{w1}\eta_{EmotionDysregulation,it} + c'_{w1i}\eta_{Mindfulness,it} + g_{DV1}time_{it} + e_{DV1it}$$

$$\eta_{Anxiety,it} = b_{w2}\eta_{EmotionDysregulation,it} + c'_{w2}\eta_{Mindfulness,it} + g_{DV2}time_{it} + e_{DV2it}$$

$$\eta_{Wellbeing,it} = b_{w3}\eta_{EmotionDysregulation,it} + c'_{w3}\eta_{Mindfulness,it} + g_{DV3}time_{it} + e_{DV3it}$$

Here, $\eta_{Variable,it}$ was the latent within-person component of variable for person *i* at time t. The coefficient a_w was the within-person association between the independent variable and the mediator. The coefficients b_{w1}, b_{w2}, and b_{w3} quantified the within-person relationships between the mediator and the three mental health outcome variables, i.e., depression symptoms, anxiety symptoms, and subjective well-being, respectively. The coefficients c'_{w1i}, c'_{w2}, and c'_{w3} represented the relationships between the independent variable and the three dependent variables, after controlling for the effect of the mediator. The random slope c'_{w1i} was assumed to follow a normal distribution with a mean of c'_{w1} and a variance of $\sigma^2_{w,c}$.

The level-2 model was the between-person mediation model. Specifically, from the independent variable to the mediator (the "a" path at the between-person level), the equation was:

$$\eta_{EmotionDysregulation,i} = \mu_M + a_b\eta_{Mindfulness,i} + e_{Mi}.$$

From the mediator to the dependent variables (the "b" paths at the between-person level), the equations were:

$$\eta_{Depression,i} = \mu_{DV1} + b_{b1}\eta_{EmotionDysregulation,i} + c'_{b1}\eta_{Mindfulness,i} + \gamma_{w,1}Gender_{it} + e_{DV1i}$$

$$\eta_{Anxiety,i} = \mu_{DV2} + b_{b2}\eta_{EmotionDysregulation,i} + c'_{b2}\eta_{Mindfulness,i} + \gamma_{w,2}Gender_{it} + e_{DV2i}$$

$$\eta_{Wellbeing,i} = \mu_{DV3} + b_{b3}\eta_{EmotionDysregulation,i} + c'_{b3}\eta_{Mindfulness,i} + e_{DV3i}.$$

Similar to the level-1 model, $\eta_{Variable,i}$ was the latent between-person component of a variable for person i. The coefficient a_b was the between-person association between the independent variable and the mediator. The coefficients b_{b1}, b_{b2}, and b_{b3} quantified the between-

person relationships between the mediator and the three mental health outcome variables, respectively. The coefficients c'_{b1}, c'_{b2}, and c'_{b3} represent the between-person relationships between the independent variable and the three dependent variables, after controlling for the effect of the mediator. The random slope c'_{w1i} was assumed to follow a normal distribution with a mean of c'_{w1} and a variance of $\sigma^2_{w,c}$. Following the traditional steps of mediation analysis [60], we fitted a multilevel mediation model without the mediator to study the overall effect of mindfulness on the outcomes. The model was estimated under missing data theory using all available data [57, ch.9]. Given the limitations listed by Hayes [61], the ratio of the indirect effect to the total effect was not calculated in determining the strength of the indirect effect. That is, this ratio could be out of the expected range between 0 and 1, and the estimate of the ratio might be highly unstable from sample to sample. Unless the sample size was fairly large, Hayes [60, p.189] recommended not "having much faith" in this measure.

Results

Table 1 summarizes the variable means, *SD*s, ranges, intraclass correlations (ICCs), and design effects. Table 2 summarizes the between-person correlations among the study variables. Specifically, dispositional mindfulness, emotion dysregulation, and mental health outcomes were correlated with each other at ps < .05 over time, ranging widely from small to large effect sizes [62]. Gender was associated with T2 and T3 dispositional mindfulness, as well as T3 depressive symptoms, anxiety symptoms, and subjective well-being, ps < .05. The variables showed substantial within-person correlations, which were captured by the random intercepts in the multilevel model. Changes in means over time were observed, suggesting the necessity of including time as a covariate.

Table 2. Zero-Order correlations of the study variables (N = 191)

Variable	(1)	(2)	(3)	(4)	(5)	(6)	(7)	(8)	(9)	(10)	(11)	(12)	(13)	(14)	(15)	(16)
(1) Gender (0=male; 1=female)	-															
**Time 1**																
(2) Dispositional mindfulness	.07	-														
(3) Emotion dysregulation	-.09	-.55***	-													
(4) Depressive symptoms	-.02	-.48***	.69***	-												
(5) Anxiety symptoms	-.11	-.42***	.65***	.77***	-											
(6) Subjective well-being	.11	.64***	-.58***	-.65***	-.56***	-										
**Time 2**																
(7) Dispositional mindfulness	.19*	.58***	-.54***	-.41***	-.37***	.57***	-									
(8) Emotion dysregulation	-.03	-.42***	.76***	.63***	.59***	-.49***	-.62***	-								
(9) Depressive symptoms	-.05	-.23**	.49***	.67***	.57***	-.41***	-.47***	.63***	-							
(10) Anxiety symptoms	-.09	-.21**	.39***	.51***	.52***	-.43***	-.47***	.52***	.73***	-						
(11) Subjective well-being	.13	.46***	-.54***	-.51***	-.40***	.68***	.70***	-.62***	-.52***	-.52***	-					
**Time 3**																
(12) Dispositional mindfulness	.17*	.53***	-.47***	-.42***	-.38***	.47***	.58***	-.54***	-.44***	-.38***	.51***	-				
(13) Emotion dysregulation	-.12	-.42***	.70***	.55***	.48***	-.44***	-.49***	.75***	.52**	.38**	-.53***	-.65***	-			
(14) Depressive symptoms	-.16*	-.45***	.53***	.62***	.57***	-.44***	-.44***	.56***	.67***	.54***	-.47***	-.61**	.68***	-		
(15) Anxiety symptoms	-.16*	-.39***	.44***	.53***	.54***	-.42***	-.36***	.48***	.56***	.56**	-.44***	-.49***	.59**	.80**	-	
(16) Subjective well-being	.20*	.50***	-.43***	-.45***	-.38***	.59***	.55***	-.47***	-.46***	-.48***	.69***	.71***	-.56***	-.64***	-.60***	-

$^{*}p < .05$, $^{**}p < .01$, $^{***}p < .001$.

Multilevel structural equation modeling

The ICCs of the studied variables, which quantified the proportion of variance in the variables attributable to individual differences, ranged from .52 (anxiety) to .73 (emotion dysregulation). This indicated substantial between-person variances, which should be considered using multilevel modeling. The design effect, defined as 1+ (average cluster size-1)×ICC, was larger than two for any of the studied variables, except for anxiety symptoms, suggesting that the hierarchical structure of the data should be considered [63]. Based on the R-squared measures for multilevel models proposed by Rights and Sterba [64], we computed the proportion of within-cluster outcome variance explained by level-1 predictors via fixed slopes and random slope variation (i.e., $R_w^{2(f_1v)}$ in the article) and the proportion of between-cluster outcome variance explained by level-2 predictors via fixed slopes (i.e., $R_b^{2(f_2)}$ in the article) for each of the three outcome variables. Specifically, mindfulness, emotion dysregulation, and time explained 23% of the within-person variances via fixed slopes and random slope variation for depression, and 18% and 30% of the within-person variances via fixed slopes for anxiety and well-being, respectively. At the between-person level, mindfulness, emotion dysregulation and gender explained 31% and 42% of the between-person variances for the first two outcome variables, respectively. Mindfulness and emotion dysregulation explained 67% of the between-person variances for well-being (see Figure 1 for details on path coefficients).

Figure 1. Multilevel mediation model of dispositional mindfulness, emotion dysregulation, and mental health outcomes. $^{*}p$ =/< .05, $^{**}p$ =/< .01, $^{***}p$ =/< .001. Unstandardized parameter estimates and standard errors in parentheses are presented.

Within-person indirect effect of dispositional mindfulness. The effect of dispositional mindfulness on depressive symptoms was negatively mediated by emotion regulation, $\widehat{ab} = -.12, p < .001$, 95% CI = [-.18, -.06], with a significant mean direct

effect, $\hat{c}_0' = -.17, p < .01$, 95% CI = [-.30, -.05]. Specifically, mindfulness predicted lower emotion dysregulation, $\hat{a} = -.31, p < .001$, 95% CI = [-.41, -.21]. Emotion dysregulation further predicted greater depressive symptoms, $\hat{b} = .40, p < .001$, 95% CI = [.26, .54]. The results regarding anxiety symptoms showed similar patterns (see Table 3). Specifically, emotion dysregulation inversely predicted by greater mindfulness was associated with greater anxiety symptoms, $\widehat{ab} = -.15, p < .001$, 95% CI = [-.23, -.07], suggesting a partial mediation effect, $\hat{c}' = -.19, p = .01$, 95% CI = [-.34, -.04].

Regarding the indirect effect of mindfulness on subjective well-being through emotion dysregulation, we found a partial positive mediation effect, $\widehat{ab} = .13, p = .002$, 95% CI = [.05, .22]; $\hat{c}' = .60, p < .001$, 95% CI = [.41, .79]. Specifically, mindfulness was negatively associated with emotion dysregulation, which in turn predicted worse subjective well-being, $\hat{b} = -.43, p < .001$, 95% CI = [-0.20, -.66] (see Table 4).

Between-person indirect effect of dispositional mindfulness. The effects of dispositional mindfulness on symptoms of depression and anxiety were found to be negatively mediated by emotion dysregulation (see Table 3). Notably, full mediation effects were found at the between-person level, as indicated by the nonsignificant direct effects of mindfulness on symptoms of depression, $\hat{c}' = .46, p = .41$, 95% CI = [-.63, 1.54] and on anxiety, $\hat{c}' = -.23$, $p = .15$, 95% CI = [-.54,.08]. Greater mindfulness was associated with lower emotion dysregulation. Emotion dysregulation, in turn, was associated with more symptoms of depression and anxiety.

The between-person indirect effect of dispositional mindfulness on subjective well-being was not significant, $\widehat{ab} = .22, p = .07$, 95% CI = [-.02, .45]. Although the “a” path between mindfulness and emotion dysregulation was significant, the “b” path between emotion

dysregulation and well-being was not, $\hat{b} = -.24, p = .08$, 95% CI = [-.50, .03] (see Table 4).

Table 3. Unstandardized parameter estimates and standard errors of the multilevel model

Parameter	*Unstandardized B (SE)*
Within-Person Effect	
Time	
→ Dispositional mindfulness	-.03 (.02)
→ Emotion dysregulation	.04 (.01)**
→ Depressive symptoms	.04 (.02)*
→ Anxiety symptoms	.07 (.02)**
→ Subjective Well-being	-.12 (.03)***
Dispositional mindfulness	
→ Emotion dysregulation	-.31 (.05)***
→ Depressive symptoms (mean, c'_{w10})	-.17 (.06)**
→ Anxiety symptoms	-.19 (.08)*
→ Subjective Well-being	.60 (.10)***
Emotion dysregulation	
→ Depressive symptoms	.40 (.07)***
→ Anxiety symptoms	.49 (.10)***
→ Subjective well-being	-.43 (.12)***
Depressive symptoms ↔ Anxiety symptoms	.06 (.01)***
Depressive symptoms ↔ Subjective Well-being	-.04 (.01)***
Anxiety symptoms ↔ Subjective Well-being	-.04 (.01)**
Between-Person Effect	
Gender (0=male; 1=female)	
→ Depressive symptoms	.03 (.09)
→ Anxiety symptoms	-.07 (.11)
Dispositional mindfulness	
→ Emotion dysregulation	-.91 (.09)***
→ Depressive symptoms	.46 (.55)
→ Anxiety symptoms	-.23 (.16)
→ Subjective Well-being	1.44 (.19)***
Emotion dysregulation	
→ Depressive symptoms	1.31 (.48)**
→ Anxiety symptoms	.62 (.14)***
→ Subjective Well-being	-.24 (.14)
Depressive symptoms ↔ Anxiety symptoms	.11 (.05)*
Depressive symptoms ↔ Subjective well-being	.01 (.05)
Anxiety symptoms ↔ Subjective well-being	-.05 (.02)**
Depressive symptoms ↔ c'_{w1i}	-.14 (.06)*
Anxiety symptoms ↔ c'_{w1i}	-.02 (.02)
Subjective well-being ↔ c'_{w1i}	-.01 (.02)
Dispositional mindfulness ↔ c'_{w1i}	.01 (.01)
Emotion dysregulation ↔ c'_{w1i}	-.02 (.02)
c'_{w1i} ↔ c'_{w1i}	.05 (.02)*

*$p < .05$, **$p < .01$, ***$p < .001$. The random coefficient c'_{w1i} represents the within-person relationship between dispositional mindfulness and depressive symptoms, after controlling for the effect of emotion dysregulation. c'_{w10} is the mean of c'_{w1i} across participants.

Table 4. Results of multilevel mediation analyses on the mediating effect of emotion dysregulation between dispositional mindfulness and mental health outcomes

Outcome	a	b	c'	ab	c
			Within-Person		
Depression		.40[.26,.54] *	-.17[-.30,-.05] *	-.12[-.18,-.06] *	-.29[-.43,-.16] *
Anxiety	-.31[-.41,-.21]*	.49[.29,.69] *	-.19[-.34,-.04] *	-.15[-.23,-.07] *	-.34[-.49,-.19] *
Subjective well-being		-.43[-.66,-.20] *	.60[.41,.79] *	.13[.05,.22] *	.73[.52,.94] *
			Between-Person		
Depression		1.31[.36,2.26] *	.46[-.63,1.54]	-1.19[-2.01,-.38] *	-.74[-1.35,-.13] *
Anxiety	-.91[-1.09,.74] *	.62[.35,.89] *	-.23[-.54,.08]	-.57[-.85,-.29] *	-.80[-1.01,-.59] *
Subjective well-being		-.24[-.50,.03]	1.44[1.07,1.82] *	.22[-.02,.45]	1.66[1.40,1.93] *

The numbers with asterisk (*) indicate significant results. Numbers within brackets are the lower and upper limits of confidence intervals. The coefficients a, b, c, and c' represent the paths from dispositional mindfulness to emotion dysregulation, from emotion dysregulation to mental health outcomes, and from dispositional mindfulness to mental health outcomes before and after controlling for the effect of emotion dysregulation. The coefficient ab represents the indirect effect of emotion dysregulation. Note that the within-person c' and c paths for depression was the average of c' and c paths across participants. The upper panel reports the results regarding the within-person level whereas the lower displays those for the between-person level.

Discussion

Building on existing theories on mindfulness, emotion regulation, and well-being [12-13], this study supported emotion dysregulation as a mediator between dispositional mindfulness and mental health outcomes in college-enrolled Chinese emerging adults. Findings based on multi-level modeling further suggested within- and between-person nuances for the associations (see Figure 1). At the within-person level, emotion dysregulation mediated between mindfulness and all mental health outcomes. Surprisingly, time was associated with worse emotion dysregulation and well-being, suggesting that psychological functioning worsened as a function of time, potentially due to an increasing level of academic stress over the school year. At the between-person level, dispositional mindfulness negatively predicted emotion dysregulation and positively predicted subjective well-being, whereas emotion dysregulation positively predicted symptoms of depression and anxiety, but not subjective well-being. These findings advanced the field by establishing within- and between-person relations between dispositional mindfulness and mental health via emotion dysregulation.

This study used multilevel modeling by partitioning between-person relations from within-person relations. Importantly, the variables were related within-person in the hypothesized directions, suggesting emotion dysregulation as a central intraindividual process linking dispositional mindfulness and mental health outcomes. By orienting to the present moment and paying attention on purpose and non-judgmentally [65], individuals became more mindful and were more capable of decentering themselves from subjective emotional experience [66], less preoccupied with invalidating, avoiding, or rejecting emotional experiences [67], more likely to disengage themselves from autopilot [68], and more likely to engage in adaptive emotion regulation [12-13]. Based on these findings, practitioners could tailor their practices and

interventions to the progress of each client. For example, to enhance subjective well-being and reduce psychological symptoms, practitioners can enhance within-person improvements in mindfulness and emotion regulation, as mindfulness affects mental health outcomes both directly and through adaptive emotion regulation.

Turning to the between-person findings, dispositional mindfulness was directly associated with emotion dysregulation and subjective well-being, but not with symptoms of depression and anxiety. These findings signified that when person *i* had a higher score in dispositional mindfulness than person *j*, then person *i* was also more likely to have a lower score in emotion dysregulation and a higher score in subjective well-being than person *j*. Despite a lack of a direct relation between dispositional mindfulness and psychological symptoms, as an explanatory variable emotion dysregulation accounted for the between-person relation. The findings resonated with recent findings conducted in the Chinese context, in that emotion dysregulation mediated between dispositional mindfulness and symptoms of depression and anxiety [10]. Surprisingly, emotion dysregulation did not hold up as a between-person mediator between dispositional mindfulness and subjective well-being. In fact, emotion dysregulation was not related to subjective well-being at all, after controlling for the effect of dispositional mindfulness. These findings contradicted recent cross-sectional studies [29,69] showing the link between Chinese college students' emotion regulation strategies and life satisfaction (i.e., a major component of subjective well-being [70]). Also, instead of general dysregulation of emotions, perhaps nuances such as regulation strategies (e.g., cognitive reappraisal) [29], regulatory flexibility [71], and affective states, frequency, and intensity [72] are more proximal factors of subjective well-being. Alternatively, perhaps over time, emotion dysregulation is more strongly associated with negative outcomes (e.g., psychological symptoms) than with positive outcomes

(e.g., positive well-being and satisfaction with life), after controlling for the effect of mindfulness. Furthermore, perhaps other stage-salient variables in emerging adulthood, such as becoming more financial independent, having greater responsibility, and having greater commitment in romantic relationships [73], are more strongly associated with positive well-being. Consequently, future studies should replicate the present findings and investigate other longitudinal predictors of well-being in the Chinese context. In terms of clinical implications, practitioners should be made aware of their clients' levels of mindfulness, emotion dysregulation, and mental health relative to other people. For example, returning to Figure 1, in relation to person *j*, person *i*'s greater level of dispositional mindfulness was associated with a lower level of emotion dysregulation and better well-being than person *j*, but not directly with fewer symptoms of depression and anxiety. Understanding these associations could help practitioners gauge the between-person importance or relevance of mindfulness in various mental health outcomes. Finally, gender was not related to psychological symptoms in the model. However, our small sample of men (i.e., 9.95%) precluded us from drawing meaningful conclusions about gender as a correlate of psychological distress.

Time was related to greater emotion dysregulation, greater symptoms of depression and anxiety, and worse subjective well-being. Contrary to previous research suggesting that individuals were more capable of emotion regulation as they mature [74], our findings suggested the opposite. It might be that our short-term longitudinal data were insufficient to capture a positive development in emotion regulation. Hence, a long-term longitudinal approach spanning over several years is necessary to verify whether this is the case. Alternatively, given the participants were emerging adults enrolled in a university, they may be facing increasing stress as they approached graduation. Future work is needed to rule out confounding variables, such as

academic stress and sample characteristics, associated with time or mental health [75].

Moving onto the mean levels, upon standardization, the current college participants reported a similar average of dispositional mindfulness, emotion dysregulation, and symptoms of depression and anxiety compared to others Chinese samples [44,46,50]. Paradoxically, our participants also reported a greater level of emotional, psychological, and social well-being than did another college sample [56]. Again, future studies should replicate the mean levels as well as the strengths of associations in other Chinese samples.

Limitations and Future Directions

The study has several limitations. First, we utilized self-report measures. Future researchers may adopt a multi-method approach by incorporating physiological and neural measures. Next, given this sample comprised mainly of emerging adults at a university, it is uncertain whether the findings can be generalized to other contexts. Future studies should examine the relation in a more representative sample from the community. In addition, the timeframes of the mental health questionnaires differ. For example, the PHQ-9 assessed depressive symptoms in the past two weeks, whereas the Mental Health Continuum Short Form assessed emotional, social, and psychological well-being in the past four weeks. Future research may consider standardizing the timeframes to increase precision of the variables. Furthermore, our participants were mainly female. Future studies with gender-balanced samples are necessary to draw meaningful conclusions for the effects of gender. Finally, researchers should translate the present findings and design targeted interventions to improve mental health outcomes.

Conclusion

This study calls attention to the relation between dispositional mindfulness and mental health through emotion dysregulation. Taking into account the effect of time, findings based on

multilevel modeling demonstrated differential effects between the within- and the between-person levels, thereby suggesting a need to partition these levels in future research. The general findings converged to underscore the association between dispositional mindfulness and different aspects of mental health in emerging adulthood, including symptoms of depression and anxiety, as well as emotional, social, and psychological well-being. Psychological intervention programs and public health campaigns geared toward enhancing mindfulness and emotion regulation merit future research investigations.

Acknowledgements

We would like to thank the reviewers for the helpful comments. We would also like to thank Ming Chen, Wing Yee Cheng, and Man Chong Leung for their assistance and contributions.

References

1. Farb NA, Anderson AK, Segal ZV. The mindful brain and emotion regulation in mood disorders. Can J Psychiatry. 2012;57(2):70-7. doi: 10.1177/070674371205700203.
2. Kabat‐Zinn J. Mindfulness‐based interventions in context: past, present, and future. Clin Psychol (New York). 2003;10(2):144-56. doi: 10.1093/clipsy.bpg016.
3. World Health Organization. Constitution 1948. In: Basic Documents. 45th ed Supplement. Geneva: WHO; 2006.
4. Baer RA, Lykins EL, Peters JR. Mindfulness and self-compassion as predictors of psychological wellbeing in long-term meditators and matched nonmeditators. J Posit Psychol. 2012;7(3):230-8. doi: 10.1080/17439760.2012.674548.
5. Bowlin SL, Baer RA. Relationships between mindfulness, self-control, and psychological functioning. Pers Individ Dif. 2012;52(3):411-5. doi: 10.1016/j.paid.2011.10.050.

6. Lyvers M, Makin C, Toms E, Thorberg FA, Samios C. Trait mindfulness in relation to emotional self-regulation and executive function. Mindfulness. 2014;5(6):619-25. doi: 10.1007/s12671-013-0213-y.
7. Rasmussen MK, Pidgeon AM. The direct and indirect benefits of dispositional mindfulness on self-esteem and social anxiety. Anxiety Stress Coping. 2011;24(2):227-33. doi: 10.1080/10615806.2010.515681.
8. Malinowski P, Lim HJ. Mindfulness at work: Positive affect, hope, and optimism mediate the relationship between dispositional mindfulness, work engagement, and well-being. Mindfulness. 2015;6(6):1250-62. doi: 10.1007/s12671-015-0388-5.
9. Petrocchi N, Ottaviani C. Mindfulness facets distinctively predict depressive symptoms after two years: The mediating role of rumination. Pers Individ Dif. 2016;93:92-6. doi: 10.1016/j.paid.2015.08.017.
10. Cheung RYM, Ng MCY. Mindfulness and symptoms of depression and anxiety: The underlying roles of awareness, acceptance, impulse control, and emotion regulation. Mindfulness. 2019;10(6):1124-35. doi: 10.1007/s12671-018-1069-y.
11. Coffey KA, Hartman M. Mechanisms of action in the inverse relationship between mindfulness and psychological distress. Complement Health Pract Rev. 2008;13(2):79-91. doi: 10.1177/1533210108316307.
12. Garland EL, Farb NA, R. Goldin P, Fredrickson BL. Mindfulness broadens awareness and builds eudaimonic meaning: A process model of mindful positive emotion regulation. Psychol Inq. 2015;26(4):293-314. doi: 10.1080/1047840X.2015.1064294.
13. Teper R, Segal ZV, Inzlicht M. Inside the mindful mind: How mindfulness enhances emotion regulation through improvements in executive control. Curr Dir Psychol Sci.

2013;22(6):449-54. doi: 10.1177/0963721413495869.

14. Chiesa A, Serretti A, Jakobsen JC. Mindfulness: Top–down or bottom–up emotion regulation strategy?. Clin Psychol Rev. 2013;33(1):82-96. doi: 10.1016/j.cpr.2012.10.006.
15. Gross JJ. The emerging field of emotion regulation: An integrative review. Rev Gen Psychol. 1998;2(3):271-99. doi: 10.1037/1089-2680.2.3.271.
16. Gratz KL, Roemer L. Multidimensional assessment of emotion regulation and dysregulation: Development, factor structure, and initial validation of the difficulties in emotion regulation scale. J Psychopathol Behav Assess. 2004;26(1):41-54. doi: 10.1023/B:JOBA.0000007455.08539.94.
17. Aldao A, Nolen-Hoeksema S, Schweizer S. Emotion-regulation strategies across psychopathology: A meta-analytic review. Clin Psychol Rev. 2010;30(2):217-37. doi: 10.1016/j.cpr.2009.11.004.
18. Gross JJ, John OP. Individual differences in two emotion regulation processes: Implications for affect, relationships, and well-being. J Pers Soc Psychol. 2003;85(2):348-62. doi: 10.1037/0022-3514.85.2.348.
19. Joormann J, Gotlib IH. Emotion regulation in depression: Relation to cognitive inhibition. Cogn Emot. 2010;24(2):281-98. doi: 10.1080/02699930903407948.
20. Martin RC, Dahlen ER. Cognitive emotion regulation in the prediction of depression, anxiety, stress, and anger. Pers Individ Differ. 2005;39(7):1249-60. doi: 10.1016/j.paid.2005.06.004.
21. Mennin DS, Heimberg RG, Turk CL, Fresco DM. Preliminary evidence for an emotion dysregulation model of generalized anxiety disorder. Behav Res Ther. 2005;43(10):1281-310. doi: 10.1016/j.brat.2004.08.008.

22. Hill CL, Updegraff JA. Mindfulness and its relationship to emotional regulation. Emotion. 2012;12(1):81-90. doi: 10.1037/a0026355.

23. Jimenez SS, Niles BL, Park CL. A mindfulness model of affect regulation and depressive symptoms: Positive emotions, mood regulation expectancies, and self-acceptance as regulatory mechanisms. Pers Individ Differ. 2010;49(6):645-50. doi: 10.1016/j.paid.2010.05.041.

24. Coffey KA, Hartman M, Fredrickson BL. Deconstructing mindfulness and constructing mental health: Understanding mindfulness and its mechanisms of action. Mindfulness. 2010;1(4):235-53. doi: 10.1007/s12671-010-0033-2.

25. Desrosiers A, Vine V, Klemanski DH, Nolen□Hoeksema S. Mindfulness and emotion regulation in depression and anxiety: Common and distinct mechanisms of action. Depress Anxiety. 2013;30(7):654-61. doi: 10.1002/da.22124.

26. Mandal SP, Arya YK, Pandey R. Mindfulness, emotion regulation, and subjective well-being: Exploring the link. SIS Journal of Projective Psychology and Mental Health. 2017;24(1):57-63.

27. Cheung RYM, Ng MCY. Being in the moment later? Testing the inverse relation between mindfulness and procrastination. Pers Individ Dif. 2019;141:123-6. doi: 10.1016/j.paid.2018.12.015.

28. Hou WK, Ng SM, Wan JHY. Changes in positive affect and mindfulness predict changes in cortisol response and psychiatric symptoms: A latent change score modelling approach. Psychol Health. 2015;30(5):551-67. doi: 10.1080/08870446.2014.990389.

29. Cheung RYM, Chung HS, Ng MC. Expressive writing and well-being in Chinese emerging adults: Is emotion regulation an underlying mechanism? Emerg Adulthood. doi:

10.1177/2167696818824719.

30. Hue MT, Lau NS. Promoting well-being and preventing burnout in teacher education: A pilot study of a mindfulness-based programme for pre-service teachers in Hong Kong. Teacher Development. 2015;19(3):381-401. doi: 10.1080/13664530.2015.1049748.
31. Lau NS, Hue MT. Preliminary outcomes of a mindfulness-based programme for Hong Kong adolescents in schools: Well-being, stress and depressive symptoms. International Journal of Children's Spirituality. 2011;16(4):315-30. doi: 10.1080/1364436X.2011.639747.
32. Wong SY, Yip BH, Mak WW, Mercer S, Cheung EY, Ling CY, Lui WW, Tang WK, Lo HH, Wu JC, Lee TM. Mindfulness-based cognitive therapy v. group psychoeducation for people with generalised anxiety disorder: Randomised controlled trial. Br J Psychiatry. 2016;209(1):68-75. doi: 10.1192/bjp.bp.115.166124.
33. Lo HH, Ng SM, Chan CL. Evaluating compassion–mindfulness therapy for recurrent anxiety and depression: A randomized control trial. Res Soc Work Pract. 2015;25(6):715-25. doi: 10.1177/1049731514537686.
34. Preacher KJ, Zyphur MJ, Zhang Z. A general multilevel SEM framework for assessing multilevel mediation. Psychol Methods. 2010;15(3):209-23. doi: 10.1037/a0020141.
35. Raudenbush SW, Bryk AS. Hierarchical linear models: Applications and data analysis methods. 2nd ed. Newbury Park: Sage; 2002.
36. Galla BM. Within-person changes in mindfulness and self-compassion predict enhanced emotional well-being in healthy, but stressed adolescents. J Adolesc. 2016;49:204-17. doi: 10.1016/j.adolescence.2016.03.016.
37. Carson JW, Carson KM, Gil KM, Baucom DH. Mindfulness-based relationship

enhancement. Behav Ther. 2004;35(3):471-94. doi: 10.1016/S0005-7894(04)80028-5.

38. Lenger KA, Gordon CL, Nguyen SP. Intra-individual and cross-partner associations between the five facets of mindfulness and relationship satisfaction. Mindfulness. 2017;8(1):171-80. doi: 10.1007/s12671-016-0590-0.
39. Tang W, Dai Q. (2018). Depressive symptoms among first-year Chinese undergraduates: The roles of socio-demographics, coping style, and social support. Psychiatry Res. 2018;270:89-96. doi: 10.1016/j.psychres.2018.09.027.
40. Shi M, Liu L, Wang ZY, Wang L. The mediating role of resilience in the relationship between big five personality and anxiety among Chinese medical students: a cross-sectional study. PLoS One. 2015;10(3):e0119916. doi: 10.1371/journal.pone.0119916.
41. Wang P, VanderWeele TJ. Empirical research on factors related to the subjective well-being of Chinese urban residents. Soc Indic Res. 2011;101(3), 447-59.
42. Arnett JJ, Žukauskienė R, Sugimura K. The new life stage of emerging adulthood at ages 18–29 years: Implications for mental health. Lancet Psychiatry. 2014;1(7):569-76. doi: 10.1016/s2215-0366(14)00080-7.
43. Feldman G, Hayes A, Kumar S, Greeson J, Laurenceau JP. Mindfulness and emotion regulation: The development and initial validation of the Cognitive and Affective Mindfulness Scale-Revised (CAMS-R). J Psychopathol Behav Assess. 2007;29(3):177-90. doi: 10.1007/s10862-006-9035-8.
44. Chan HL, Lo LY, Lin M, Thompson N. Revalidation of the cognitive and affective mindfulness scale—Revised (CAMS-R) with its newly developed Chinese version (Ch-CAMS-R). J Pac Rim Psychol. 2016;10:1-10. doi: 10.1017/prp.2015.4.
45. Wang L, Liu H, Du W, Li Z. Test of difficulties in emotion regulation scale in Chinese

people. China J Health Psychol. 2007;15:336-40.

46. Li J, Han ZR, Gao MM, Sun X, Ahemaitijiang N. Psychometric properties of the Chinese version of the Difficulties in Emotion Regulation Scale (DERS): Factor structure, reliability, and validity. Psychol Assess, 2018;30(5):e1-9. doi: 10.1037/pas0000582.
47. Kroenke K, Spitzer RL, Williams JB. The PHQ□9: Validity of a brief depression severity measure. J Gen Intern Med. 2001;16(9):606-13. doi: 10.1046/j.1525-1497.2001.016009606.x.
48. Yu X, Tam WW, Wong PT, Lam TH, Stewart SM. The Patient Health Questionnaire-9 for measuring depressive symptoms among the general population in Hong Kong. Compr Psychiatry. 2012;53(1):95-102. doi: 10.1016/j.comppsych.2010.11.002.
49. Zhang YL, Liang W, Chen ZM, Zhang HM, Zhang JH, Weng XQ, Yang SC, Zhang L, Shen LS, Zhang YL. Validity and reliability of Patient Health Questionnaire□9 and Patient Health Questionnaire□2 to screen for depression among college students in China. Asia Pac Psychiatry. 2013;5(4):268-75. doi: 10.1111/appy.12103.
50. Zhang RT, Wang Y, Yang ZY, Li Y, Wang YM, Cheung EFC, Sham DHK, Yang T, Barkus, EJ, Chan RCK. Network structure of anticipatory pleasure and risk features: Evidence from a large college sample. PsyCh J. 2020;9(2):223-33. doi: 10.1002/pchj.331.
51. Spitzer RL, Kroenke K, Williams JB, Löwe B. A brief measure for assessing generalized anxiety disorder: The GAD-7. Arch Intern Med. 2006;166(10):1092-7. doi: 10.1001/archinte.166.10.1092.
52. He XY, Li C, Qian J, Cui H, Wu W. Reliability and validity of a generalized anxiety disorder scale in general hospital outpatients. Shanghai Arch Psychiatry. 2010;22(4):200-3.
53. Tong X, An D, McGonigal A, Park SP, Zhou D. Validation of the Generalized Anxiety

Disorder-7 (GAD-7) among Chinese people with epilepsy. Epilepsy Res. 2016;120:31-6. doi:10.1016/j.eplepsyres.2015.11.019.

54. Keyes CL. Brief description of the mental health continuum short form (MHC-SF). 2009. Available from: http://www.sociology.emory.edu/ckeyes/

Including the retrieving day. Sometimes, the link is no longer available

55. Guo C, Tomson G, Guo J, Li X, Keller C, Söderqvist F. Psychometric evaluation of the Mental Health Continuum-Short Form (MHC-SF) in Chinese adolescents–a methodological study. Health Qual Life Outcomes. 2015;13(1):198. doi: 10.1186/s12955-015-0394-2.

56. Qian K, Yin K, Zhang L. (2015). Predictive effects of learning burnout on positive and negative mental health in college students. Chin Ment Health J. 2015;29(3):236-40.

57. Muthén LK, Muthén, BO. Mplus User's Guide. 8th ed. Los Angeles: Muthén & Muthén; 1998-2017.

58. Park IJ, Wang L, Williams DR, Alegría M. Does anger regulation mediate the discrimination–mental health link among Mexican-origin adolescents? A longitudinal mediation analysis using multilevel modeling. Dev Psychol. 2017;53(2):340-52. doi: 10.1037/dev0000235.

59. Wang LP, Maxwell SE. On disaggregating between-person and within-person effects with longitudinal data using multilevel models. Psychol Methods. 2015;20(1):63-83. doi: 10.1037/met0000030.

60. Baron RM, Kenny DA. The moderator–mediator variable distinction in social psychological research: Conceptual, strategic, and statistical considerations. J Pers Soc Psychol. 1986;51(6):1173-82. doi: 10.1037/0022-3514.51.6.1173.

61. Hayes AF. Introduction to mediation, moderation, and conditional process analysis: A

regression-based approach. New York: Guilford Press; 2013.

62. Cohen J. Statistical power analysis for the behavioral sciences. 2nd ed. Erlbaum; 1988.
63. Muthén BO, Satorra A. Complex sample data in structural equation modeling. Sociol Methodol. 1995:267-316. doi: 10.2307/271070.
64. Rights JD, Sterba SK. Quantifying explained variance in multilevel models: An integrative framework for defining R-squared measures. Psychol Methods. 2019;24(3):309-38. doi: 10.1037/met0000184.
65. Kabat-Zinn J. Wherever you go, there you are: Mindfulness meditation in everyday life. New York: Hyperion; 1994.
66. Bhambhani Y, Cabral G. Evaluating nonattachment and decentering as possible mediators of the link between mindfulness and psychological distress in a nonclinical college sample. J Evid Based Complement Alternat Med. 2016;21(4):295-305. doi: 10.1177/2156587215607109.
67. Hayes AM, Feldman G. Clarifying the construct of mindfulness in the context of emotion regulation and the process of change in therapy. Clin Psychol (New York). 2004;11(3):255-62. doi: 10.1093/clipsy.bph080.
68. Germer C, Siegel RD, Fulton PR, editors. Mindfulness and psychotherapy. New York: Guilford Publications; 2016.
69. Cheung RYM, Leung MC, Chiu HT, Kwan JL, Yee LT, Hou WK. Family functioning and psychological outcomes in emerging adulthood: Savoring positive experiences as a mediating mechanism. J Soc Pers Relatsh. 2019;36(9):2693-713. doi: 10.1177/0265407518798499.
70. Diener ED, Emmons RA, Larsen RJ, Griffin S. The satisfaction with life scale. J Pers

Assess. 1985;49(1):71-5. doi: 10.1207/s15327752jpa4901_13.

71. Bonanno GA, Burton CL. Regulatory flexibility: An individual differences perspective on coping and emotion regulation. Perspect Psychol Sci. 2013;8(6):591-612. doi: 10.1177/1745691613504116.

72. DeSteno D, Gross JJ, Kubzansky L. Affective science and health: The importance of emotion and emotion regulation. Health Psychol. 2013;32(5):474-86. doi: 10.1037/a0030259.

73. Arnett JJ. Emerging adulthood: A theory of development from the late teens through the twenties. Am. Psychol. 2000;55(5):469-80. doi: 10.1037/0003-066x.55.5.469.

74. Zimmermann P, Iwanski A. Emotion regulation from early adolescence to emerging adulthood and middle adulthood: Age differences, gender differences, and emotion-specific developmental variations. Int J Behav Dev. 2014;38(2):182-94. doi: 10.1177/016502541351540.

75. Pedersen D. Stress carry-over and college student health outcomes. Coll Stud J. 2012;46(3):620-7.

Figure

Click here to access/download;Figure;Figure 1 (Model Findings) 11-7-2020.docx

Figure 1. Multilevel mediation model of dispositional mindfulness, emotion dysregulation, and mental health outcomes. *p =/< .05, **p =/< .01, ***p =/< .001. Unstandardized parameter estimates and standard errors in parentheses are presented. For simplicity, the random slope of depressive symptoms regressed on dispositional mindfulness is not depicted.

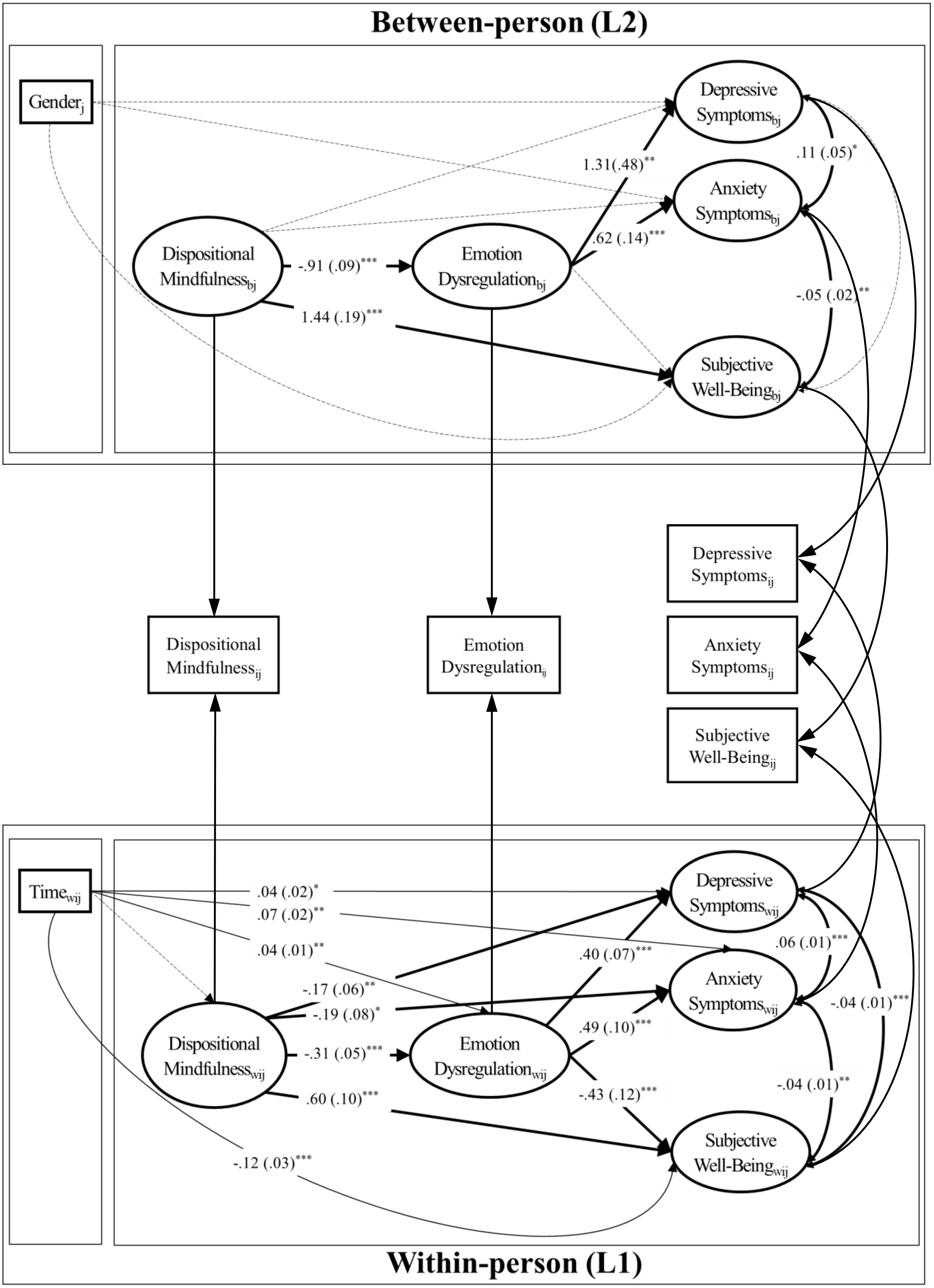

Data associated with this study

Click here to access/download
Supporting Information
data.dat

Dispositional Mindfulness and Mental Health in Chinese Emerging Adults: A Multilevel Model with Emotion Dysregulation as a Mediator

Rebecca Y. M. Cheung[1], Zijun Ke[2*], Melody C. Y. Ng[3]

[1] Department of Early Childhood Education, Centre for Child and Family Studies, and Centre for Psychosocial Health, The Education University of Hong Kong, Hong Kong SAR, China

[2] Department of Psychology, Sun Yat-Sen University, Guangdong, China

[3] Department of Psychology, The Chinese University of Hong Kong, Hong Kong SAR, China

[*] Corresponding author

Email (Zijun Ke): keziyun@mail.sysu.edu.cn

Abstract

Using a multilevel model, this study examined emotion dysregulation as a mediator between dispositional mindfulness and mental health among Chinese emerging adults. Participants were 191 Chinese emerging adults (female = 172) between 18 and 27 years old (M = 21.06 years, SD = 2.01 years), who completed a questionnaire that assessed their dispositional mindfulness, emotion dysregulation, and mental health outcomes for three times over 12 months, with a three-month lag between each time point. Within-person analysis revealed that emotion dysregulation mediated between dispositional mindfulness and mental health outcomes, including subjective well-being and symptoms of depression and anxiety. Time was positively associated with emotion dysregulation and negatively associated with symptoms of depression and anxiety. Between-person analysis revealed that emotion dysregulation negatively mediated between mindfulness and symptoms of depression and anxiety, but not subjective well-being. These findings call attention to emotion dysregulation as a mediator between dispositional mindfulness and psychological outcomes, particularly of symptoms of depression and anxiety. Notably, the multilevel analysis offered a more nuanced approach to distinguish between within-person vs. between-person processes. Attesting to the relations established in western societies, these findings are applicable to emerging adults in the Chinese context. Evidence was advanced to inform translational research efforts that promote dispositional mindfulness as an asset of mental health.

Keywords: dispositional mindfulness, emotion dysregulation, mental health, multilevel modeling, subjective well-being

Introduction

Mindfulness is a mental state whereby individuals attend to their cognitive, physical, and emotional experience in the present moment nonjudgmentally [1-2]. Previous research suggested that mindfulness is associated with mental health outcomes, which encompass both a low level of psychological symptoms but also better mental well-being [3]. Notably, mindfulness is associated with outcomes including better subjective well-being, and fewer depressive and anxiety symptoms [4-6]. Moving beyond the simple association between mindfulness and mental health, recent studies have identified several mediating psychological mechanisms, including greater self-esteem [7], more positive affect, hope, and optimism [8], lower rumination [9], and better emotion regulation [10-11], thereby suggesting possible chains of processes between mindfulness and mental health. Of note, emotion regulation has received much theoretical and empirical attention as a mediating mechanism [12-13].

Emotion regulation is defined as a process in modulating emotions and emotional responses [14-15]. According to Gratz and Roemer [16], adaptive emotion regulation involves such aspects as acceptance of emotional responses, engagement in goal directed behavior, maintenance of emotional clarity and awareness, and access to adaptive emotion regulation strategies, such as cognitive reappraisal and savoring ability. On the contrary, emotion dysregulation involves difficulties in acquiring regulatory skills or a frequent use of maladaptive strategies, such as rumination, avoidance, and expressive suppression [17-18]. Previous research suggested that our capability to adaptively regulate emotions is central to subjective well-being [18]. Emotion dysregulation not only undermines well-being, but also gives rise to clinical depression, anxiety, psychological distress, and elevated depressive and anxiety symptoms [10, 17, 19-21].

Theoretical Tenets of Emotion Regulation as a Mediator between Mindfulness and Mental Health Outcomes

As a proximal correlate of mental well-being, people's capability to regulate emotions is associated with mindfulness [6, 22-23]. More specifically, Teper et al. [13] theorized that mindfulness is linked to emotion regulation through heightened metacognitive awareness, non-judgmental acceptance, and executive control, all of which are crucial to well-being. Likewise, in mindfulness-to-meaning theory, Garland et al. [12] postulated that mindfulness promotes purpose in life by virtue of greater metacognitive awareness, broadened attention to context, adaptive emotion regulation, ability to savor hedonic experience, and prosocial actions. Both theoretical frameworks suggest close connections between mindfulness, emotion regulation, and psychological well-being.

Supporting the frameworks, cross-sectional studies revealed that emotion regulation mediated between dispositional mindfulness and lower levels of depression and anxiety [11, 23-25], as well as better life satisfaction [26]. Findings based on a clinically depressed and anxious sample suggested that emotion regulation strategies, including cognitive reappraisal and rumination, mediated between dispositional mindfulness and symptoms of depression and anxiety [25]. These findings resonated with research conducted with non-clinical emerging adult samples, in that emotion regulation mediated the inverse relation between dispositional mindfulness and depressive symptoms, in that dispositional mindfulness was related to better emotion regulation and fewer depressive symptoms [23]. In a similar vein, Coffey and Hartman [11] and Coffey et al. [24] found that emerging adults' regulation of negative emotions, impulse control, and goal engagement mediated between dispositional mindfulness and well-being. Altogether, evidence to-date converged to indicate emotion regulation as a vital process between

dispositional mindfulness and well-being.

Processes of Mental Health in the Chinese Context

Among the handful of studies conducted in the Chinese context, dispositional mindfulness predicted better impulse control, better emotion regulation, less procrastination, and fewer psychological distress [10, 27]. Moreover, changes in mindful awareness were associated with subsequent changes of stress response and anxiety symptoms [28]. Similarly, changes in emotion regulation strategies were associated with changes in changes in depressive symptoms, life satisfaction, and general health over time [29]. As such, timing and changes were crucial in linking mindfulness, emotion regulation, and mental health outcomes. Beyond dispositional mindfulness, mindfulness training also increased Chinese adolescents' and adults' well-being and psychological adjustment in Hong Kong [30-31]. In a randomized controlled trial involving a Chinese sample with clinical anxiety, Wong et al. [32] revealed that participants who took an 8-week mindfulness-based cognitive therapy had an increased dispositional mindfulness and a reduced anxiety. Such an increase was found to be more significantly than did participants who received psycho-education based on cognitive behavioral therapy principles. Similar findings were reported in another randomized controlled trial involving a Chinese sample with recurrent symptoms of depression and anxiety [33]. In this study, participants who received an 8-week compassion–mindfulness therapy showed more mental health improvements than did participants from the waitlist control group. These findings highlight that the mechanisms associated with mindfulness are integral to mental health in the Chinese context.

Within- vs. Between-Person Effects

A majority of research to-date, as reviewed above, focuses on between-person associations of mindfulness, emotion regulation or dysregulation, and mental health [e.g., 5-11].

Only a handful of studies partitioned within-person from between-person processes. Between- and within-person effects are conceptually and statistically independent. Within-person effect aids the understanding of *intraindividual* processes underlying well-being. For instance, when mindfulness of person *i* increases, then his or her own mental health is expected to also increase over time. Between-person effect accounts for *interindividual* processes underlying well-being. That is, when person *i* has a higher score in mindfulness than person *j*, then person *i* is expected to have a higher score in mental health than is person *j*. Of note, partitioning among within- and between-person associations is important, because otherwise the results could sometimes be biased [34] or uninterpretable [35].

Several studies to-date have demonstrated the utility of within-person analyses in mindfulness and well-being. For example, Galla [36] indicated that a five-day mindfulness training was associated with significant within-person reductions in rumination and depressive symptoms, as well as increases in life satisfaction in healthy adolescents. In addition, many of the improvements were maintained at the three-month follow-up assessment. In another study, an 8-week mindfulness training gave rise to an inverse within-person association between mindfulness and relationship stress [37]. Apart from stress, psychological symptoms, and subjective well-being, within-person associations were also found between dispositional mindfulness and relationship satisfaction [38]. By investigating within-person effects, these studies methodologically precluded confounding between-person factors from the within-person associations between mindfulness and other psychological processes.

This Study

Aside from the studies described in the previous section, past research predominantly examined between-person effects among mindfulness, emotion regulation, and mental health [5-

11]. Although some studies have begun to examine within-person effects [36-38], few, if any, have teased apart within-person from between-person effects. Although within- and between-person findings converged to suggest the mental health benefits of mindfulness, it remains unclear as to whether its clinical significance on emotion regulation and various mental health outcomes differ.

The present study aims to investigate longitudinal within- vs between-person mediating effect between dispositional mindfulness and mental health outcomes via emotion dysregulation. Through this analytic technique, the intraindividual processes and the interindividual processes could be identified. Based on the literature [23-25], we hypothesized that greater dispositional mindfulness would be related to lower emotion dysregulation. Lower emotion dysregulation would, then, be related to fewer symptoms of depression and anxiety, as well as greater subjective well-being. We further hypothesized that the relation between dispositional mindfulness and mental health outcomes would be mediated by emotion dysregulation at both within- and between-person levels. The strength of associations may vary as a function of the level. As stated earlier, time was an important measure in assessing changes within and between variables [28-29]. As such, time was included as a within-person covariate to control for dispositional mindfulness, emotion dysregulation, and mental health outcomes. Based on previous research showing the links between gender and depressive symptoms [39], anxiety symptoms [40], and subjective well-being [41], gender was included as a between-person covariate for mental health outcomes.

Method

Participants

Participants were 191 Chinese emerging adults (female = 172; 90.05%) recruited online

through two mass emails at a major public university in Hong Kong. Inclusion criteria included college-enrolled emerging adults who were proficient in Chinese, within the age range between 18 and 29 years old [42], who agreed to participate for three time points over the course of a year. Participants were between 18 and 27 years old, with a mean age of 21.06 years at Time 1 (*SD* = 2.01 years). As for the retention rate, 93.72% (*n* = 179) of the participants from Time 1 (T1) were retained at Time 2 (T2); 94.41% (*n* = 169) of the participants from T2 were retained at Time 3 (T3).

Procedures

The study was approved by the Human Research Ethics Committee, The Education University of Hong Kong (Approval #: 2015-2016-0352), prior to its implementation. All procedures performed were in accordance with the ethical standards of the institutional research committee and the 1964 Helsinki declaration and its later amendments. A written consent had been obtained from each participant prior to the beginning of the study. At the first time point, participants completed baseline measures. They were then invited to complete follow-up questionnaires twice, with a three-month lag between time points, over the course of a year. Each packet took approximately 30 minutes to complete. Upon completion, participants received a supermarket coupon at each time point (totaling HK$200, i.e., ~US$25.71).

Measures

Dispositional Mindfulness. The 12-item Cognitive and Affective Mindfulness Scale – Revised (CAMS-R) [43] was used to assess dispositional mindfulness on a 4-point scale from 1 (*rarely/ not at all*) to 4 (*almost always*). The CAMS-R assessed three dimensions of mindfulness including awareness, acceptance, and attention. A sample item included, "I can usually describe how I feel at the moment in considerable detail." The item scores were

averaged, such that higher scores indicated a greater level of mindfulness. The measure was validated previously in a Chinese college sample with adequate convergent validity, reliability, and factor structure [44]. Specifically, participants from this study had a similar mean level of dispositional mindfulness (see Table 1). In this study, the test-retest correlations of the measure between T1, T2, and T3 were moderate at .53 - .58, *ps* < .001. Cronbach's alpha of the measure = .91 - .94 between T1 and T3. More specifically, between T1 and T3, Cronbach's alpha of the awareness subscale = .63 - .80, the acceptance subscale = .62 - .79, and the attention subscale = .77 - .81.

Emotion Dysregulation. The 36-item Difficulties in Emotional Regulation Scale (DERS) [16] was used to assess emotional regulation on a 5-point scale from 1 (*almost never*) to 5 (*almost always*). The measure included 6 subscales including non-acceptance of emotions, difficulties in engaging in goal-directed behavior, impulse control difficulties, lack of emotional awareness, limited access to emotion regulation strategies, and lack of emotional clarity. Sample items included, "I am clear about my feelings" and "When I'm upset, I believe that my feelings are valid and important." The final scores were then averaged, such that higher scores indicated a greater emotion dysregulation. The measure had been validated in a Chinese sample [45]. Compared to another Chinese adult sample [46], participants from this study had a similar mean level of emotion dysregulation (see Table 1). In this study, the test-retest correlations of the measure between T1, T2, and T3 were .70 - .76, *ps* < .001. Cronbach's alpha of the measure = .83 - .87 between T1 and T3. More specifically, between T1 and T3, Cronbach's alpha of the non-acceptance of emotions subscale = .83 - .87, the difficulties in engaging in goal-directed behavior subscale = .56 - .75, the impulse control difficulties subscale = .77 - .85, the lack of emotional awareness subscale = .72 - .75, the limited access to emotion regulation strategies

subscales = .83 - .87, and the lack of emotional clarity subscale = .74 - .77.

Table 1. Means and standard deviations of the study variables (N = 191)

Variable	*M*	*SD*	Range	ICC	Design Effect
(1) Gender (0=male; 1=female)	-	-			
**Time 1**					
(2) Dispositional mindfulness	2.85	.43	2.20		
(3) Emotion dysregulation	2.03	.49	2.50		
(4) Depressive symptoms	.61	.50	2.67		
(5) Anxiety symptoms	.61	.58	3.00		
(6) Subjective well-being	4.49	.86	4.21		
**Time 2**					
(7) Dispositional mindfulness	2.80	.48	2.40		
(8) Emotion dysregulation	2.12	.51	2.30		
(9) Depressive symptoms	.68	.56	3.00		
(10) Anxiety symptoms	.65	.64	3.00		
(11) Subjective well-being	4.28	.86	4.50		
**Time 3**					
(12) Dispositional mindfulness	2.78	.52	2.80		
(13) Emotion dysregulation	2.12	.54	2.77		
(14) Depressive symptoms	.74	.61	2.89		
(15) Anxiety symptoms	.81	.68	3.00		
(16) Subjective well-being	4.15	.92	4.86		
**Across Time Points**					
Dispositional mindfulness	2.81	.48		.56	2.01
Emotion dysregulation	2.09	.51		.73	2.31
Depressive symptoms	.68	.56		.63	2.13
Anxiety symptoms	.68	.63		.52	1.93
Subjective well-being	4.32	.89		.64	2.14

Depressive Symptoms. The 9-item Patient Health Questionnaire-9 (PHQ-9) [47] was used to measure depressive symptoms in the past two weeks on a 4-point scale from 0 (*not at all*) to 3 (*nearly every day*). A sample item included, “Feeling bad about yourself - or that you are a failure or have let yourself or your family down?” The item scores were averaged to form a score of depressive symptoms, with higher scores indicating more symptoms. The measure has previously been validated in a Chinese community sample and demonstrated an adequate factor structure, construct validity, internal consistency, and test-retest reliability [48]. Based on a cutoff score of 11 for the detection of depressive disorders [49], upon rescaling, our participants’

summed scores of 5.44 - 6.67 between T1 and T3 were below the clinical cutoff. Compared to another Chinese college sample [50] participants from this study had a comparable level of depressive symptoms (see Table 1). However, a total of 20, 21, and 28 participants at T1, T2, and T3, respectively, reported a summed score of above 11. In this study, the test-retest correlations of the measure between T1, T2, and T3 were .62 - .67, *ps* < .001. Cronbach's alpha of the measure = .87 - .91 between T1 and T3.

Anxiety Symptoms. The 7-item Generalized Anxiety Disorder-7 measure (GAD-7) [51] was used to measure anxiety symptoms in the past two weeks on a 4-point scale from 0 (*not at all*) to 3 (*nearly every day*). A sample item included, "Feeling nervous, anxious or on edge." The item scores were averaged to form a score of anxiety symptoms, with higher scores indicating more symptoms. The measure has been validated in a Chinese sample of hospital outpatients [52] and a sample of Chinese individuals with epilepsy [53]. Based on a cutoff score of 10 for the detection of generalized anxiety disorder [51], upon rescaling our participants' summed scores between 4.22 and 5.67 between T1 and T3 were below the clinical cutoff. Compared to another Chinese college sample [50] participants from this study reported a similar level of anxiety symptoms (see Table 1). However, a total of 14, 20, and 26 participants at T1, T2, and T3, respectively, reported a total score of above 10. In this study, the test-retest correlations of the measure between T1, T2, and T3 were .52 - .56, *ps* < .001. Cronbach's alpha of the measure = 93 - .95 between T1 and T3.

Subjective Well-being. The 14-item Mental Health Continuum Short Form (MHC-SF) [54] was used as a measure for well-being over the past four weeks. A 6-point scale ranging from 0 (*never*) to 5 (*every day*) was used, with sample items including, "how often did you feel satisfied with life" (*emotional well-being*), "how often did you feel that that you had something

important to contribute to society (*social well-being*) and “how often did you feel that your life has a sense of direction or meaning to it” (*psychological well-being*). The item scores were averaged to form a mean score, with higher scores indicating better well-being. MHC-SF had been previously validated in a Chinese adolescent sample, and yielded good validity and reliability [55]. Compared to another Chinese college sample [56], participants from this study had a greater mean level of subjective well-being (see Table 1). In this study, the test-retest correlations of the measure between T1, T2, and T3 were .59 - .69, $ps < .001$. Cronbach’s alpha of the measure = .79 - .94 between T1 and T3. More specifically, between T1 and T3, Cronbach’s alpha of the emotional well-being subscale = .90 - .94, the social well-being subscale = .79 - .86, and the psychological well-being subscale = .92 - .93.

Data Analysis

Multilevel mediation analyses were conducted using Mplus 8.0 [57] to study the mediating effect of emotion dysregulation on the relationship between mindfulness and mental health outcomes, including depressive symptoms, anxiety symptoms, and subjective well-being, at both the within- and between-person levels. Multilevel modeling has been used in previous research for panel data (e.g., [58]), with a minimum of three waves of observations to partition between within- and between-person associations, which, as introduced previously, have distinctive practical implications. Compared to the traditional multilevel modeling approach to multilevel mediation, the multilevel structural equation modeling (MSEM) approach has the advantage of having a smaller bias.

In this study, we relied on the MSEM approach. Covariates at both levels were considered. At the within-person level, a time variable was included to control for the potential change over time [59]. At the between-person level, gender was included to control for possible

gender differences in symptoms of anxiety and depression.

Regarding whether random slopes should be considered, with only three measurement occasions at level 1, the model allowing all mediation paths (i.e., "a" path from mindfulness to emotion dysregulation, "b" paths from mindful awareness to mental health, and "c" paths from emotion dysregulation to mental health) and the slope of time to be random could not be identified. Therefore, we tested possible random mediation paths sequentially. Results showed that none of the "a", "b", and "c′" paths showed substantial individual differences, except for the "c′" path for depression. Additionally, we tested possible random slopes of time (change between two consecutive measurement occasions) and found no significant individual differences. Consequently, all mediation paths and slopes of time were fixed to be invariant across participants, except for the "c′" path for depression in the subsequent analyses.

In sum, the multilevel mediation model under consideration was as follows. The independent variable, the mediator, and the three dependent variables were partitioned into two latent parts:

$$\text{DispositionalMindfulness}_{\text{it}} = \eta_{Mindfulness,it} + \eta_{Mindfulness,i}$$

$$\text{EmotionDysregulation}_{\text{it}} = \eta_{EmotionDysregulation,it} + \eta_{EmotionDysregulation,i}$$

$$\text{Depression}_{\text{it}} = \eta_{Depression,it} + \eta_{Depression,i}$$

$$\text{Anxiety}_{\text{it}} = \eta_{Anxiety,it} + \eta_{Anxity,i}$$

$$\text{Wellbeing}_{\text{it}} = \eta_{Wellbeing,it} + \eta_{Wellbeing,i}$$

More specifically, the level-1 model defined the within-person mediation model. Between the independent variable and the mediator (the fixed "a" path), the equation was:

$$\eta_{EmotionDysregulation,it} = a_w \eta_{Mindfulness,it} + g_M time_{it} + e_{Mit}.$$

From the mediator to the dependent variables (the fixed "b" paths), the equations were:

$$\eta_{Depression,it} = b_{w1}\eta_{EmotionDysregulation,it} + c'_{w1i}\eta_{Mindfulness,it} + g_{DV1}time_{it} + e_{DV1it}$$

$$\eta_{Anxiety,it} = b_{w2}\eta_{EmotionDysregulation,it} + c'_{w2}\eta_{Mindfulness,it} + g_{DV2}time_{it} + e_{DV2it}$$

$$\eta_{Wellbeing,it} = b_{w3}\eta_{EmotionDysregulation,it} + c'_{w3}\eta_{Mindfulness,it} + g_{DV3}time_{it} + e_{DV3it}$$

Here, $\eta_{Variable,it}$ was the latent within-person component of variable for person i at time t. The coefficient a_w was the within-person association between the independent variable and the mediator. The coefficients b_{w1}, b_{w2}, and b_{w3} quantified the within-person relationships between the mediator and the three mental health outcome variables, i.e., depression symptoms, anxiety symptoms, and subjective well-being, respectively. The coefficients c'_{w1i}, c'_{w2}, and c'_{w3} represented the relationships between the independent variable and the three dependent variables, after controlling for the effect of the mediator. The random slope c'_{w1i} was assumed to follow a normal distribution with a mean of c'_{w1} and a variance of $\sigma^2_{w,c}$.

The level-2 model was the between-person mediation model. Specifically, from the independent variable to the mediator (the “a” path at the between-person level), the equation was:

$$\eta_{EmotionDysregulation,i} = \mu_M + a_b\eta_{Mindfulness,i} + e_{Mi}.$$

From the mediator to the dependent variables (the “b” paths at the between-person level), the equations were:

$$\eta_{Depression,i} = \mu_{DV1} + b_{b1}\eta_{EmotionDysregulation,i} + c'_{b1}\eta_{Mindfulness,i} + \gamma_{w,1}Gender_{it} + e_{DV1i}$$

$$\eta_{Anxiety,i} = \mu_{DV2} + b_{b2}\eta_{EmotionDysregulation,i} + c'_{b2}\eta_{Mindfulness,i} + \gamma_{w,2}Gender_{it} + e_{DV2i}$$

$$\eta_{Wellbeing,i} = \mu_{DV3} + b_{b3}\eta_{EmotionDysregulation,i} + c'_{b3}\eta_{Mindfulness,i} + e_{DV3i}.$$

Similar to the level-1 model, $\eta_{Variable,i}$ was the latent between-person component of a variable for person i. The coefficient a_b was the between-person association between the independent variable and the mediator. The coefficients b_{b1}, b_{b2}, and b_{b3} quantified the between-

person relationships between the mediator and the three mental health outcome variables, respectively. The coefficients c'_{b1}, c'_{b2}, and c'_{b3} represent the between-person relationships between the independent variable and the three dependent variables, after controlling for the effect of the mediator. The random slope c'_{w1i} was assumed to follow a normal distribution with a mean of c'_{w1} and a variance of $\sigma^2_{w,c}$. Following the traditional steps of mediation analysis [60], we fitted a multilevel mediation model without the mediator to study the overall effect of mindfulness on the outcomes. The model was estimated under missing data theory using all available data [57, ch.9]. Given the limitations listed by Hayes [61], the ratio of the indirect effect to the total effect was not calculated in determining the strength of the indirect effect. That is, this ratio could be out of the expected range between 0 and 1, and the estimate of the ratio might be highly unstable from sample to sample. Unless the sample size was fairly large, Hayes [60, p.189] recommended not "having much faith" in this measure.

Results

Table 1 summarizes the variable means, *SD*s, ranges, intraclass correlations (ICCs), and design effects. Table 2 summarizes the between-person correlations among the study variables. Specifically, dispositional mindfulness, emotion dysregulation, and mental health outcomes were correlated with each other at $ps < .05$ over time, ranging widely from small to large effect sizes [62]. Gender was associated with T2 and T3 dispositional mindfulness, as well as T3 depressive symptoms, anxiety symptoms, and subjective well-being, $ps < .05$. The variables showed substantial within-person correlations, which were captured by the random intercepts in the multilevel model. Changes in means over time were observed, suggesting the necessity of including time as a covariate.

Table 2. Zero-Order correlations of the study variables (N = 191)

Variable	(1)	(2)	(3)	(4)	(5)	(6)	(7)	(8)	(9)	(10)	(11)	(12)	(13)	(14)	(15)	(16)
(1) Gender (0=male; 1=female)	-															
**Time 1**																
(2) Dispositional mindfulness	.07	-														
(3) Emotion dysregulation	-.09	-.55***	-													
(4) Depressive symptoms	-.02	-.48***	.69***	-												
(5) Anxiety symptoms	-.11	-.42***	.65***	.77***	-											
(6) Subjective well-being	.11	.64***	-.58***	-.65***	-.56***	-										
**Time 2**																
(7) Dispositional mindfulness	.19*	.58***	-.54***	-.41***	-.37***	.57***	-									
(8) Emotion dysregulation	-.03	-.42***	.76***	.63***	.59***	-.49***	-.62***	-								
(9) Depressive symptoms	-.05	-.23**	.49***	.67***	.57***	-.41***	-.47***	.63***	-							
(10) Anxiety symptoms	-.09	-.21**	.39***	.51***	.52***	-.43***	-.47***	.52***	.73***	-						
(11) Subjective well-being	.13	.46***	-.54***	-.51***	-.40***	.68***	.70***	-.62***	-.52***	-.52***	-					
**Time 3**																
(12) Dispositional mindfulness	.17*	.53***	-.47***	-.42***	-.38***	.47***	.58***	-.54***	-.44***	-.38***	.51***	-				
(13) Emotion dysregulation	-.12	-.42***	.70***	.55***	.48***	-.44***	-.49***	.75***	.52**	.38**	-.53***	-.65***	-			
(14) Depressive symptoms	-.16*	-.45***	.53***	.62***	.57***	-.44***	-.44***	.56***	.67***	.54***	-.47***	-.61**	.68***	-		
(15) Anxiety symptoms	-.16*	-.39***	.44***	.53***	.54***	-.42***	-.36***	.48***	.56***	.56**	-.44***	-.49***	.59**	.80**	-	
(16) Subjective well-being	.20*	.50***	-.43***	-.45***	-.38***	.59***	.55***	-.47***	-.46***	-.48***	.69***	.71***	-.56***	-.64***	-.60***	-

$^{*}p < .05$, $^{**}p < .01$, $^{***}p < .001$.

Multilevel structural equation modeling

The ICCs of the studied variables, which quantified the proportion of variance in the variables attributable to individual differences, ranged from .52 (anxiety) to .73 (emotion dysregulation). This indicated substantial between-person variances, which should be considered using multilevel modeling. The design effect, defined as 1+ (average cluster size-1)×ICC, was larger than two for any of the studied variables, except for anxiety symptoms, suggesting that the hierarchical structure of the data should be considered [63]. Based on the R-squared measures for multilevel models proposed by Rights and Sterba [64], we computed the proportion of within-cluster outcome variance explained by level-1 predictors via fixed slopes and random slope variation (i.e., $R_w^{2(f_1v)}$ in the article) and the proportion of between-cluster outcome variance explained by level-2 predictors via fixed slopes (i.e., $R_b^{2(f_2)}$ in the article) for each of the three outcome variables. Specifically, mindfulness, emotion dysregulation, and time explained 23% of the within-person variances via fixed slopes and random slope variation for depression, and 18% and 30% of the within-person variances via fixed slopes for anxiety and well-being, respectively. At the between-person level, mindfulness, emotion dysregulation and gender explained 31% and 42% of the between-person variances for the first two outcome variables, respectively. Mindfulness and emotion dysregulation explained 67% of the between-person variances for well-being (see Figure 1 for details on path coefficients).

Figure 1. Multilevel mediation model of dispositional mindfulness, emotion dysregulation, and mental health outcomes. $^*p$ =/< .05, $^{**}p$ =/< .01, $^{***}p$ =/< .001. Unstandardized parameter estimates and standard errors in parentheses are presented.

Within-person indirect effect of dispositional mindfulness. The effect of dispositional mindfulness on depressive symptoms was negatively mediated by emotion regulation, $\widehat{ab} = -.12, p < .001$, 95% CI = [-.18, -.06], with a significant mean direct

effect, $\hat{c}_0' = -.17, p < .01$, 95% CI = [-.30, -.05]. Specifically, mindfulness predicted lower emotion dysregulation, $\hat{a} = -.31, p < .001$, 95% CI = [-.41, -.21]. Emotion dysregulation further predicted greater depressive symptoms, $\hat{b} = .40, p < .001$, 95% CI = [.26, .54]. The results regarding anxiety symptoms showed similar patterns (see Table 3). Specifically, emotion dysregulation inversely predicted by greater mindfulness was associated with greater anxiety symptoms, $\widehat{ab} = -.15, p < .001$, 95% CI = [-.23, -.07], suggesting a partial mediation effect, $\hat{c}' = -.19, p = .01$, 95% CI = [-.34, -.04].

Regarding the indirect effect of mindfulness on subjective well-being through emotion dysregulation, we found a partial positive mediation effect, $\widehat{ab} = .13, p = .002$, 95% CI = [.05, .22]; $\hat{c}' = .60, p < .001$, 95% CI = [.41, .79]. Specifically, mindfulness was negatively associated with emotion dysregulation, which in turn predicted worse subjective well-being, $\hat{b} = -.43, p < .001$, 95% CI = [-0.20, -.66] (see Table 4).

Between-person indirect effect of dispositional mindfulness. The effects of dispositional mindfulness on symptoms of depression and anxiety were found to be negatively mediated by emotion dysregulation (see Table 3). Notably, full mediation effects were found at the between-person level, as indicated by the nonsignificant direct effects of mindfulness on symptoms of depression, $\hat{c}' = .46, p = .41$, 95% CI = [-.63, 1.54] and on anxiety, $\hat{c}' = -.23$, $p = .15$, 95% CI = [-.54,.08]. Greater mindfulness was associated with lower emotion dysregulation. Emotion dysregulation, in turn, was associated with more symptoms of depression and anxiety.

The between-person indirect effect of dispositional mindfulness on subjective well-being was not significant, $\widehat{ab} = .22, p = .07$, 95% CI = [-.02, .45]. Although the "a" path between mindfulness and emotion dysregulation was significant, the "b" path between emotion

dysregulation and well-being was not, $\hat{b} = -.24$, $p = .08$, 95% CI = [-.50, .03] (see Table 4).

Table 3. Unstandardized parameter estimates and standard errors of the multilevel model

Parameter	*Unstandardized B (SE)*
Within-Person Effect	
Time	
→ Dispositional mindfulness	-.03 (.02)
→ Emotion dysregulation	.04 (.01)**
→ Depressive symptoms	.04 (.02)*
→ Anxiety symptoms	.07 (.02)**
→ Subjective Well-being	-.12 (.03)***
Dispositional mindfulness	
→ Emotion dysregulation	-.31 (.05)***
→ Depressive symptoms (mean, c'_{w10})	-.17 (.06)**
→ Anxiety symptoms	-.19 (.08)*
→ Subjective Well-being	.60 (.10)***
Emotion dysregulation	
→ Depressive symptoms	.40 (.07)***
→ Anxiety symptoms	.49 (.10)***
→ Subjective well-being	-.43 (.12)***
Depressive symptoms ←→ Anxiety symptoms	.06 (.01)***
Depressive symptoms ←→ Subjective Well-being	-.04 (.01)***
Anxiety symptoms ←→ Subjective Well-being	-.04 (.01)**
Between-Person Effect	
Gender (0=male; 1=female)	
→ Depressive symptoms	.03 (.09)
→ Anxiety symptoms	-.07 (.11)
Dispositional mindfulness	
→ Emotion dysregulation	-.91 (.09)***
→ Depressive symptoms	.46 (.55)
→ Anxiety symptoms	-.23 (.16)
→ Subjective Well-being	1.44 (.19)***
Emotion dysregulation	
→ Depressive symptoms	1.31 (.48)**
→ Anxiety symptoms	.62 (.14)***
→ Subjective Well-being	-.24 (.14)
Depressive symptoms ←→ Anxiety symptoms	.11 (.05)*
Depressive symptoms ←→ Subjective well-being	.01 (.05)
Anxiety symptoms ←→ Subjective well-being	-.05 (.02)**
Depressive symptoms ←→ c'_{w1i}	-.14 (.06)*
Anxiety symptoms ←→ c'_{w1i}	-.02 (.02)
Subjective well-being ←→ c'_{w1i}	-.01 (.02)
Dispositional mindfulness ←→ c'_{w1i}	.01 (.01)
Emotion dysregulation ←→ c'_{w1i}	-.02 (.02)
c'_{w1i} ←→ c'_{w1i}	.05 (.02)*

**p* < .05, ***p* < .01, ****p* < .001. The random coefficient c'_{w1i} represents the within-person relationship between dispositional mindfulness and depressive symptoms, after controlling for the effect of emotion dysregulation. c'_{w10} is the mean of c'_{w1i} across participants.

Table 4. Results of multilevel mediation analyses on the mediating effect of emotion dysregulation between dispositional mindfulness and mental health outcomes

Outcome	a	b	c'	ab	c
			Within-Person		
Depression		.40[.26,.54]*	-.17[-.30,-.05]*	-.12[-.18,-.06]*	-.29[-.43,-.16]*
Anxiety	-.31[-.41,-.21]*	.49[.29,.69]*	-.19[-.34,-.04]*	-.15[-.23,-.07]*	-.34[-.49,-.19]*
Subjective well-being		-.43[-.66,-.20]*	.60[.41,.79]*	.13[.05,.22]*	.73[.52,.94]*
			Between-Person		
Depression		1.31[.36,2.26]*	.46[-.63,1.54]	-1.19[-2.01,-.38]*	-.74[-1.35,-.13]*
Anxiety	-.91[-1.09,.74]*	.62[.35,.89]*	-.23[-.54,.08]	-.57[-.85,-.29]*	-.80[-1.01,-.59]*
Subjective well-being		-.24[-.50,.03]	1.44[1.07,1.82]*	.22[-.02,.45]	1.66[1.40,1.93]*

The numbers with asterisk (*) indicate significant results. Numbers within brackets are the lower and upper limits of confidence intervals. The coefficients a, b, c, and c' represent the paths from dispositional mindfulness to emotion dysregulation, from emotion dysregulation to mental health outcomes, and from dispositional mindfulness to mental health outcomes before and after controlling for the effect of emotion dysregulation. The coefficient ab represents the indirect effect of emotion dysregulation. Note that the within-person c' and c paths for depression was the average of c' and c paths across participants. The upper panel reports the results regarding the within-person level whereas the lower displays those for the between-person level.

Discussion

Building on existing theories on mindfulness, emotion regulation, and well-being [12-13], this study supported emotion dysregulation as a mediator between dispositional mindfulness and mental health outcomes in college-enrolled Chinese emerging adults. Findings based on multi-level modeling further suggested within- and between-person nuances for the associations (see Figure 1). At the within-person level, emotion dysregulation mediated between mindfulness and all mental health outcomes. Surprisingly, time was associated with worse emotion dysregulation and well-being, suggesting that psychological functioning worsened as a function of time, potentially due to an increasing level of academic stress over the school year. At the between-person level, dispositional mindfulness negatively predicted emotion dysregulation and positively predicted subjective well-being, whereas emotion dysregulation positively predicted symptoms of depression and anxiety, but not subjective well-being. These findings advanced the field by establishing within- and between-person relations between dispositional mindfulness and mental health via emotion dysregulation.

This study used multilevel modeling by partitioning between-person relations from within-person relations. Importantly, the variables were related within-person in the hypothesized directions, suggesting emotion dysregulation as a central intraindividual process linking dispositional mindfulness and mental health outcomes. By orienting to the present moment and paying attention on purpose and non-judgmentally [65], individuals became more mindful and were more capable of decentering themselves from subjective emotional experience [66], less preoccupied with invalidating, avoiding, or rejecting emotional experiences [67], more likely to disengage themselves from autopilot [68], and more likely to engage in adaptive emotion regulation [12-13]. Based on these findings, practitioners could tailor their practices and

interventions to the progress of each client. For example, to enhance subjective well-being and reduce psychological symptoms, practitioners can enhance within-person improvements in mindfulness and emotion regulation, as mindfulness affects mental health outcomes both directly and through adaptive emotion regulation.

Turning to the between-person findings, dispositional mindfulness was directly associated with emotion dysregulation and subjective well-being, but not with symptoms of depression and anxiety. These findings signified that when person *i* had a higher score in dispositional mindfulness than person *j*, then person *i* was also more likely to have a lower score in emotion dysregulation and a higher score in subjective well-being than person *j*. Despite a lack of a direct relation between dispositional mindfulness and psychological symptoms, as an explanatory variable emotion dysregulation accounted for the between-person relation. The findings resonated with recent findings conducted in the Chinese context, in that emotion dysregulation mediated between dispositional mindfulness and symptoms of depression and anxiety [10]. Surprisingly, emotion dysregulation did not hold up as a between-person mediator between dispositional mindfulness and subjective well-being. In fact, emotion dysregulation was not related to subjective well-being at all, after controlling for the effect of dispositional mindfulness. These findings contradicted recent cross-sectional studies [29,69] showing the link between Chinese college students' emotion regulation strategies and life satisfaction (i.e., a major component of subjective well-being [70]). Also, instead of general dysregulation of emotions, perhaps nuances such as regulation strategies (e.g., cognitive reappraisal) [29], regulatory flexibility [71], and affective states, frequency, and intensity [72] are more proximal factors of subjective well-being. Alternatively, perhaps over time, emotion dysregulation is more strongly associated with negative outcomes (e.g., psychological symptoms) than with positive outcomes

(e.g., positive well-being and satisfaction with life), after controlling for the effect of mindfulness. Furthermore, perhaps other stage-salient variables in emerging adulthood, such as becoming more financial independent, having greater responsibility, and having greater commitment in romantic relationships [73], are more strongly associated with positive well-being. Consequently, future studies should replicate the present findings and investigate other longitudinal predictors of well-being in the Chinese context. In terms of clinical implications, practitioners should be made aware of their clients' levels of mindfulness, emotion dysregulation, and mental health relative to other people. For example, returning to Figure 1, in relation to person *j*, person *i*'s greater level of dispositional mindfulness was associated with a lower level of emotion dysregulation and better well-being than person *j*, but not directly with fewer symptoms of depression and anxiety. Understanding these associations could help practitioners gauge the between-person importance or relevance of mindfulness in various mental health outcomes. Finally, gender was not related to psychological symptoms in the model. However, our small sample of men (i.e., 9.95%) precluded us from drawing meaningful conclusions about gender as a correlate of psychological distress.

Time was related to greater emotion dysregulation, greater symptoms of depression and anxiety, and worse subjective well-being. Contrary to previous research suggesting that individuals were more capable of emotion regulation as they mature [74], our findings suggested the opposite. It might be that our short-term longitudinal data were insufficient to capture a positive development in emotion regulation. Hence, a long-term longitudinal approach spanning over several years is necessary to verify whether this is the case. Alternatively, given the participants were emerging adults enrolled in a university, they may be facing increasing stress as they approached graduation. Future work is needed to rule out confounding variables, such as

academic stress and sample characteristics, associated with time or mental health [75].

Moving onto the mean levels, upon standardization, the current college participants reported a similar average of dispositional mindfulness, emotion dysregulation, and symptoms of depression and anxiety compared to others Chinese samples [44,46,50]. Paradoxically, our participants also reported a greater level of emotional, psychological, and social well-being than did another college sample [56]. Again, future studies should replicate the mean levels as well as the strengths of associations in other Chinese samples.

Limitations and Future Directions

The study has several limitations. First, we utilized self-report measures. Future researchers may adopt a multi-method approach by incorporating physiological and neural measures. Next, given this sample comprised mainly of emerging adults at a university, it is uncertain whether the findings can be generalized to other contexts. Future studies should examine the relation in a more representative sample from the community. In addition, the timeframes of the mental health questionnaires differ. For example, the PHQ-9 assessed depressive symptoms in the past two weeks, whereas the Mental Health Continuum Short Form assessed emotional, social, and psychological well-being in the past four weeks. Future research may consider standardizing the timeframes to increase precision of the variables. Furthermore, our participants were mainly female. Future studies with gender-balanced samples are necessary to draw meaningful conclusions for the effects of gender. Finally, researchers should translate the present findings and design targeted interventions to improve mental health outcomes.

Conclusion

This study calls attention to the relation between dispositional mindfulness and mental health through emotion dysregulation. Taking into account the effect of time, findings based on

multilevel modeling demonstrated differential effects between the within- and the between-person levels, thereby suggesting a need to partition these levels in future research. The general findings converged to underscore the association between dispositional mindfulness and different aspects of mental health in emerging adulthood, including symptoms of depression and anxiety, as well as emotional, social, and psychological well-being. Psychological intervention programs and public health campaigns geared toward enhancing mindfulness and emotion regulation merit future research investigations.

Acknowledgements

We would like to thank the reviewers for the helpful comments. We would also like to thank Ming Chen, Wing Yee Cheng, and Man Chong Leung for their assistance and contributions.

References

1. Farb NA, Anderson AK, Segal ZV. The mindful brain and emotion regulation in mood disorders. Can J Psychiatry. 2012;57(2):70-7. doi: 10.1177/070674371205700203.
2. Kabat□Zinn J. Mindfulness□based interventions in context: past, present, and future. Clin Psychol (New York). 2003;10(2):144-56. doi: 10.1093/clipsy.bpg016.
3. World Health Organization. Constitution 1948. In: Basic Documents. 45th ed Supplement. Geneva: WHO; 2006.
4. Baer RA, Lykins EL, Peters JR. Mindfulness and self-compassion as predictors of psychological wellbeing in long-term meditators and matched nonmeditators. J Posit Psychol. 2012;7(3):230-8. doi: 10.1080/17439760.2012.674548.
5. Bowlin SL, Baer RA. Relationships between mindfulness, self-control, and psychological functioning. Pers Individ Dif. 2012;52(3):411-5. doi: 10.1016/j.paid.2011.10.050.

6. Lyvers M, Makin C, Toms E, Thorberg FA, Samios C. Trait mindfulness in relation to emotional self-regulation and executive function. Mindfulness. 2014;5(6):619-25. doi: 10.1007/s12671-013-0213-y.
7. Rasmussen MK, Pidgeon AM. The direct and indirect benefits of dispositional mindfulness on self-esteem and social anxiety. Anxiety Stress Coping. 2011;24(2):227-33. doi: 10.1080/10615806.2010.515681.
8. Malinowski P, Lim HJ. Mindfulness at work: Positive affect, hope, and optimism mediate the relationship between dispositional mindfulness, work engagement, and well-being. Mindfulness. 2015;6(6):1250-62. doi: 10.1007/s12671-015-0388-5.
9. Petrocchi N, Ottaviani C. Mindfulness facets distinctively predict depressive symptoms after two years: The mediating role of rumination. Pers Individ Dif. 2016;93:92-6. doi: 10.1016/j.paid.2015.08.017.
10. Cheung RYM, Ng MCY. Mindfulness and symptoms of depression and anxiety: The underlying roles of awareness, acceptance, impulse control, and emotion regulation. Mindfulness. 2019;10(6):1124-35. doi: 10.1007/s12671-018-1069-y.
11. Coffey KA, Hartman M. Mechanisms of action in the inverse relationship between mindfulness and psychological distress. Complement Health Pract Rev. 2008;13(2):79-91. doi: 10.1177/1533210108316307.
12. Garland EL, Farb NA, R. Goldin P, Fredrickson BL. Mindfulness broadens awareness and builds eudaimonic meaning: A process model of mindful positive emotion regulation. Psychol Inq. 2015;26(4):293-314. doi: 10.1080/1047840X.2015.1064294.
13. Teper R, Segal ZV, Inzlicht M. Inside the mindful mind: How mindfulness enhances emotion regulation through improvements in executive control. Curr Dir Psychol Sci.

2013;22(6):449-54. doi: 10.1177/0963721413495869.

14. Chiesa A, Serretti A, Jakobsen JC. Mindfulness: Top–down or bottom–up emotion regulation strategy?. Clin Psychol Rev. 2013;33(1):82-96. doi: 10.1016/j.cpr.2012.10.006.
15. Gross JJ. The emerging field of emotion regulation: An integrative review. Rev Gen Psychol. 1998;2(3):271-99. doi: 10.1037/1089-2680.2.3.271.
16. Gratz KL, Roemer L. Multidimensional assessment of emotion regulation and dysregulation: Development, factor structure, and initial validation of the difficulties in emotion regulation scale. J Psychopathol Behav Assess. 2004;26(1):41-54. doi: 10.1023/B:JOBA.0000007455.08539.94.
17. Aldao A, Nolen-Hoeksema S, Schweizer S. Emotion-regulation strategies across psychopathology: A meta-analytic review. Clin Psychol Rev. 2010;30(2):217-37. doi: 10.1016/j.cpr.2009.11.004.
18. Gross JJ, John OP. Individual differences in two emotion regulation processes: Implications for affect, relationships, and well-being. J Pers Soc Psychol. 2003;85(2):348-62. doi: 10.1037/0022-3514.85.2.348.
19. Joormann J, Gotlib IH. Emotion regulation in depression: Relation to cognitive inhibition. Cogn Emot. 2010;24(2):281-98. doi: 10.1080/02699930903407948.
20. Martin RC, Dahlen ER. Cognitive emotion regulation in the prediction of depression, anxiety, stress, and anger. Pers Individ Differ. 2005;39(7):1249-60. doi: 10.1016/j.paid.2005.06.004.
21. Mennin DS, Heimberg RG, Turk CL, Fresco DM. Preliminary evidence for an emotion dysregulation model of generalized anxiety disorder. Behav Res Ther. 2005;43(10):1281-310. doi: 10.1016/j.brat.2004.08.008.

22. Hill CL, Updegraff JA. Mindfulness and its relationship to emotional regulation. Emotion. 2012;12(1):81-90. doi: 10.1037/a0026355.

23. Jimenez SS, Niles BL, Park CL. A mindfulness model of affect regulation and depressive symptoms: Positive emotions, mood regulation expectancies, and self-acceptance as regulatory mechanisms. Pers Individ Differ. 2010;49(6):645-50. doi: 10.1016/j.paid.2010.05.041.

24. Coffey KA, Hartman M, Fredrickson BL. Deconstructing mindfulness and constructing mental health: Understanding mindfulness and its mechanisms of action. Mindfulness. 2010;1(4):235-53. doi: 10.1007/s12671-010-0033-2.

25. Desrosiers A, Vine V, Klemanski DH, Nolen□Hoeksema S. Mindfulness and emotion regulation in depression and anxiety: Common and distinct mechanisms of action. Depress Anxiety. 2013;30(7):654-61. doi: 10.1002/da.22124.

26. Mandal SP, Arya YK, Pandey R. Mindfulness, emotion regulation, and subjective well-being: Exploring the link. SIS Journal of Projective Psychology and Mental Health. 2017;24(1):57-63.

27. Cheung RYM, Ng MCY. Being in the moment later? Testing the inverse relation between mindfulness and procrastination. Pers Individ Dif. 2019;141:123-6. doi: 10.1016/j.paid.2018.12.015.

28. Hou WK, Ng SM, Wan JHY. Changes in positive affect and mindfulness predict changes in cortisol response and psychiatric symptoms: A latent change score modelling approach. Psychol Health. 2015;30(5):551-67. doi: 10.1080/08870446.2014.990389.

29. Cheung RYM, Chung HS, Ng MC. Expressive writing and well-being in Chinese emerging adults: Is emotion regulation an underlying mechanism? Emerg Adulthood. doi:

10.1177/2167696818824719.

30. Hue MT, Lau NS. Promoting well-being and preventing burnout in teacher education: A pilot study of a mindfulness-based programme for pre-service teachers in Hong Kong. Teacher Development. 2015;19(3):381-401. doi: 10.1080/13664530.2015.1049748.
31. Lau NS, Hue MT. Preliminary outcomes of a mindfulness-based programme for Hong Kong adolescents in schools: Well-being, stress and depressive symptoms. International Journal of Children's Spirituality. 2011;16(4):315-30. doi: 10.1080/1364436X.2011.639747.
32. Wong SY, Yip BH, Mak WW, Mercer S, Cheung EY, Ling CY, Lui WW, Tang WK, Lo HH, Wu JC, Lee TM. Mindfulness-based cognitive therapy v. group psychoeducation for people with generalised anxiety disorder: Randomised controlled trial. Br J Psychiatry. 2016;209(1):68-75. doi: 10.1192/bjp.bp.115.166124.
33. Lo HH, Ng SM, Chan CL. Evaluating compassion–mindfulness therapy for recurrent anxiety and depression: A randomized control trial. Res Soc Work Pract. 2015;25(6):715-25. doi: 10.1177/1049731514537686.
34. Preacher KJ, Zyphur MJ, Zhang Z. A general multilevel SEM framework for assessing multilevel mediation. Psychol Methods. 2010;15(3):209-23. doi: 10.1037/a0020141.
35. Raudenbush SW, Bryk AS. Hierarchical linear models: Applications and data analysis methods. 2nd ed. Newbury Park: Sage; 2002.
36. Galla BM. Within-person changes in mindfulness and self-compassion predict enhanced emotional well-being in healthy, but stressed adolescents. J Adolesc. 2016;49:204-17. doi: 10.1016/j.adolescence.2016.03.016.
37. Carson JW, Carson KM, Gil KM, Baucom DH. Mindfulness-based relationship

enhancement. Behav Ther. 2004;35(3):471-94. doi: 10.1016/S0005-7894(04)80028-5.

38. Lenger KA, Gordon CL, Nguyen SP. Intra-individual and cross-partner associations between the five facets of mindfulness and relationship satisfaction. Mindfulness. 2017;8(1):171-80. doi: 10.1007/s12671-016-0590-0.

39. Tang W, Dai Q. (2018). Depressive symptoms among first-year Chinese undergraduates: The roles of socio-demographics, coping style, and social support. Psychiatry Res. 2018;270:89-96. doi: 10.1016/j.psychres.2018.09.027.

40. Shi M, Liu L, Wang ZY, Wang L. The mediating role of resilience in the relationship between big five personality and anxiety among Chinese medical students: a cross-sectional study. PLoS One. 2015;10(3):e0119916. doi: 10.1371/journal.pone.0119916.

41. Wang P, VanderWeele TJ. Empirical research on factors related to the subjective well-being of Chinese urban residents. Soc Indic Res. 2011;101(3), 447-59.

42. Arnett JJ, Žukauskienė R, Sugimura K. The new life stage of emerging adulthood at ages 18–29 years: Implications for mental health. Lancet Psychiatry. 2014;1(7):569-76. doi: 10.1016/s2215-0366(14)00080-7.

43. Feldman G, Hayes A, Kumar S, Greeson J, Laurenceau JP. Mindfulness and emotion regulation: The development and initial validation of the Cognitive and Affective Mindfulness Scale-Revised (CAMS-R). J Psychopathol Behav Assess. 2007;29(3):177-90. doi: 10.1007/s10862-006-9035-8.

44. Chan HL, Lo LY, Lin M, Thompson N. Revalidation of the cognitive and affective mindfulness scale—Revised (CAMS-R) with its newly developed Chinese version (Ch-CAMS-R). J Pac Rim Psychol. 2016;10:1-10. doi: 10.1017/prp.2015.4.

45. Wang L, Liu H, Du W, Li Z. Test of difficulties in emotion regulation scale in Chinese

people. China J Health Psychol. 2007;15:336-40.

46. Li J, Han ZR, Gao MM, Sun X, Ahemaitijiang N. Psychometric properties of the Chinese version of the Difficulties in Emotion Regulation Scale (DERS): Factor structure, reliability, and validity. Psychol Assess, 2018;30(5):e1-9. doi: 10.1037/pas0000582.

47. Kroenke K, Spitzer RL, Williams JB. The PHQ□9: Validity of a brief depression severity measure. J Gen Intern Med. 2001;16(9):606-13. doi: 10.1046/j.1525-1497.2001.016009606.x.

48. Yu X, Tam WW, Wong PT, Lam TH, Stewart SM. The Patient Health Questionnaire-9 for measuring depressive symptoms among the general population in Hong Kong. Compr Psychiatry. 2012;53(1):95-102. doi: 10.1016/j.comppsych.2010.11.002.

49. Zhang YL, Liang W, Chen ZM, Zhang HM, Zhang JH, Weng XQ, Yang SC, Zhang L, Shen LS, Zhang YL. Validity and reliability of Patient Health Questionnaire□9 and Patient Health Questionnaire□2 to screen for depression among college students in China. Asia Pac Psychiatry. 2013;5(4):268-75. doi: 10.1111/appy.12103.

50. Zhang RT, Wang Y, Yang ZY, Li Y, Wang YM, Cheung EFC, Sham DHK, Yang T, Barkus, EJ, Chan RCK. Network structure of anticipatory pleasure and risk features: Evidence from a large college sample. PsyCh J. 2020;9(2):223-33. doi: 10.1002/pchj.331.

51. Spitzer RL, Kroenke K, Williams JB, Löwe B. A brief measure for assessing generalized anxiety disorder: The GAD-7. Arch Intern Med. 2006;166(10):1092-7. doi: 10.1001/archinte.166.10.1092.

52. He XY, Li C, Qian J, Cui H, Wu W. Reliability and validity of a generalized anxiety disorder scale in general hospital outpatients. Shanghai Arch Psychiatry. 2010;22(4):200-3.

53. Tong X, An D, McGonigal A, Park SP, Zhou D. Validation of the Generalized Anxiety

Disorder-7 (GAD-7) among Chinese people with epilepsy. Epilepsy Res. 2016;120:31-6. doi:10.1016/j.eplepsyres.2015.11.019.

54. Keyes CL. Brief description of the mental health continuum short form (MHC-SF). 2009. Available from: http://www.sociology.emory.edu/ckeyes/

55. Guo C, Tomson G, Guo J, Li X, Keller C, Söderqvist F. Psychometric evaluation of the Mental Health Continuum-Short Form (MHC-SF) in Chinese adolescents–a methodological study. Health Qual Life Outcomes. 2015;13(1):198. doi: 10.1186/s12955-015-0394-2.

56. Qian K, Yin K, Zhang L. (2015). Predictive effects of learning burnout on positive and negative mental health in college students. Chin Ment Health J. 2015;29(3):236-40.

57. Muthén LK, Muthén, BO. Mplus User's Guide. 8th ed. Los Angeles: Muthén & Muthén; 1998-2017.

58. Park IJ, Wang L, Williams DR, Alegría M. Does anger regulation mediate the discrimination–mental health link among Mexican-origin adolescents? A longitudinal mediation analysis using multilevel modeling. Dev Psychol. 2017;53(2):340-52. doi: 10.1037/dev0000235.

59. Wang LP, Maxwell SE. On disaggregating between-person and within-person effects with longitudinal data using multilevel models. Psychol Methods. 2015;20(1):63-83. doi: 10.1037/met0000030.

60. Baron RM, Kenny DA. The moderator–mediator variable distinction in social psychological research: Conceptual, strategic, and statistical considerations. J Pers Soc Psychol. 1986;51(6):1173-82. doi: 10.1037/0022-3514.51.6.1173.

61. Hayes AF. Introduction to mediation, moderation, and conditional process analysis: A

regression-based approach. New York: Guilford Press; 2013.

62. Cohen J. Statistical power analysis for the behavioral sciences. 2nd ed. Erlbaum; 1988.
63. Muthén BO, Satorra A. Complex sample data in structural equation modeling. Sociol Methodol. 1995:267-316. doi: 10.2307/271070.
64. Rights JD, Sterba SK. Quantifying explained variance in multilevel models: An integrative framework for defining R-squared measures. Psychol Methods. 2019;24(3):309-38. doi: 10.1037/met0000184.
65. Kabat-Zinn J. Wherever you go, there you are: Mindfulness meditation in everyday life. New York: Hyperion; 1994.
66. Bhambhani Y, Cabral G. Evaluating nonattachment and decentering as possible mediators of the link between mindfulness and psychological distress in a nonclinical college sample. J Evid Based Complement Alternat Med. 2016;21(4):295-305. doi: 10.1177/2156587215607109.
67. Hayes AM, Feldman G. Clarifying the construct of mindfulness in the context of emotion regulation and the process of change in therapy. Clin Psychol (New York). 2004;11(3):255-62. doi: 10.1093/clipsy.bph080.
68. Germer C, Siegel RD, Fulton PR, editors. Mindfulness and psychotherapy. New York: Guilford Publications; 2016.
69. Cheung RYM, Leung MC, Chiu HT, Kwan JL, Yee LT, Hou WK. Family functioning and psychological outcomes in emerging adulthood: Savoring positive experiences as a mediating mechanism. J Soc Pers Relatsh. 2019;36(9):2693-713. doi: 10.1177/0265407518798499.
70. Diener ED, Emmons RA, Larsen RJ, Griffin S. The satisfaction with life scale. J Pers

Assess. 1985;49(1):71-5. doi: 10.1207/s15327752jpa4901_13.

71. Bonanno GA, Burton CL. Regulatory flexibility: An individual differences perspective on coping and emotion regulation. Perspect Psychol Sci. 2013;8(6):591-612. doi: 10.1177/1745691613504116.

72. DeSteno D, Gross JJ, Kubzansky L. Affective science and health: The importance of emotion and emotion regulation. Health Psychol. 2013;32(5):474-86. doi: 10.1037/a0030259.

73. Arnett JJ. Emerging adulthood: A theory of development from the late teens through the twenties. Am. Psychol. 2000;55(5):469-80. doi: 10.1037/0003-066x.55.5.469.

74. Zimmermann P, Iwanski A. Emotion regulation from early adolescence to emerging adulthood and middle adulthood: Age differences, gender differences, and emotion-specific developmental variations. Int J Behav Dev. 2014;38(2):182-94. doi: 10.1177/016502541351540.

75. Pedersen D. Stress carry-over and college student health outcomes. Coll Stud J. 2012;46(3):620-7.

We would like to thank the three reviewers for the thoughtful and constructive comments. Below, we outline the revisions that we have made to address these comments. The revisions have also been highlighted in yellow in the revised manuscript for your convenience.

Reviewer #1

General Comments

Comment:

Thank you for the interesting manuscript, studying emotion regulation as a mediator between mindfulness and mental health in the Chinese young adult population. I am happy to read that both the within- and between subject factors are taken into consideration in this study design.

Our response:

We thank Reviewer #1 for the positive comments.

Comment:

A general remark relates to the umbrella term 'mental health' used in the research question, this remains rather broad. The introduction includes a description, but throughout it is not always clear how mental health is defined with respect to design and outcomes. The separate measures (depression, anxiety, and subjective wellbeing) are sometimes interchanged with the broader mental health concept. Does this then refer to a composite measure of these questionnaires?

Our response:

Thank you for the comment. Our definition is consistent with the definition of health provided by the World Health Organization [3]. Specifically, we refer mental health to better mental well-being and low levels of psychological symptoms.

Comment:

A very high percentage of the study population is female (90%), this is correctly mentioned as a limitation at the end of the manuscript, but briefly. Gender is taken as a covariate in the analyses and conclusions are drawn on these analyses in the beginning of the discussion. This seems to me a big leap and I would advise to be careful with this statement. Reflecting on this earlier in the discussion would help. Another suggestion is to change the number into percentage for easy reading.

Our response:

Thank you for the comment. We have now used percentage and number for easy reading (p. 7). In the Discussion, we have removed, "[g]ender was not related to symptoms of depression and anxiety." We have instead stated (p. 23), "gender was not

related to psychological symptoms in the model. However, our small sample of men (i.e., 9.95%) precluded us from drawing meaningful conclusions about gender as a correlate of psychological distress."

Comment:

In the discussion, more attention could be payed to the implications of the findings for the wellbeing of the Chinese population. Some elements of the introduction, methods and results do not resonate in this last section.

Our response:

Thank you for the comment. We have now incorporated existing findings of Chinese samples in the Discussion to make the manuscript more coherent. For example, we have added (p. 22), "[t]he findings resonated with recent findings conducted in the Chinese context, in that emotion dysregulation mediated between dispositional mindfulness and symptoms of depression and anxiety [10]." We have also noted (p. 22), "[t]hese findings contradicted recent cross-sectional studies [29,69] showing the link between Chinese college students' emotion regulation strategies and life satisfaction (i.e., a major component of subjective well-being [70])." By situating our findings to the Chinese context, we hope our readers are better informed.

Introduction

Comment:

The introduction provides clear evidence for the link between mindfulness, emotion regulation and mental health. The importance of considering all three factors is furthermore evident. I did have some difficulties keeping track of the order in which the evidence is discussed. The three concepts are sometimes mixed within a paragraph aiming to focus on one of the concepts. A clearer distinction would aid readers in the process. The rational for examining both within- and between subject findings simultaneously (an important part of this manuscript) could use some elaboration.

Our response:

In the revised introduction, we broadly discussed the role of mindfulness in mental health (p. 3) and then moved onto emotion regulation as a predictor of mental health (p. 3). In the next section, we briefly discussed Teper et al.'s theoretical framework [13] and Garland et al.'s framework [21] concerning the mediating role of emotion regulation (p. 4), which was followed by empirical findings (p. 4-5). To situate the study in the Chinese context, we further discussed the findings in Chinese samples (p. 5), with mindfulness as a predictor of emotion regulation and mental health. To ensure that our audience can fully grasp the meanings of within- vs.

between-person effects, we discussed the differences, with examples in the context of mindfulness, emotion regulation, and mental health correlates. Of note, partitioning among within- and between-person associations is important, because otherwise the results could sometimes be biased [34] or uninterpretable [35] (p. 6).

We hope that the flow has now provided a clearer picture in supporting the present study.

Comment:

In the first paragraph, I would avoid using the words adjustment outcomes and change these to mental health outcomes to make the link explicit.

Our response:

We thank the reviewer for the comment. We have revised the introduction by changing “adjustment outcomes” to “mental health outcomes.”

Introduction: Section 'processes of mental health in the Chinese context'

Comment:

Section 'processes of mental health in the Chinese context': This section contains several long sentences with unclear structures.

Our response:

The long sentences have now been shortened to increase the readability (p. 5).

Comment:

‘similar findings were suggested’ needs to be ‘were found’.

Our response:

We have now updated the sentence on page 5.

Comment:

I would highlight a study into emotion regulation in this context to balance the section (e.g., take out a mindfulness example and replace it with one for emotion regulation).

Our response:

Thank you for the comment. We have now highlighted a study (p. 5) showing that changes in emotion regulation strategies were associated with changes in changes in depressive symptoms, life satisfaction, and general health over time [29].

Comment:

‘These findings highlight’ in present tense.

Our response:

We have revised the introduction by changing “These findings highlighted” to “These findings highlight” on page 5.

Introduction: Section 'within- vs. between-person effects'

Comment:

What about emotion regulation in the first sentence?

Our response:

Thank you for the comment. The first sentence has now been updated to, “[a] majority of research to-date focuses on between-person associations of mindfulness, emotion dysregulation, and mental health.”

Comment:

’Partitioning between within- and between-person’: consider using a synonym (e.g. among).

Our response:

We have changed “Partitioning between within- and between-person associations” to “Partitioning among within- and between-person associations” on page 6.

Comment:

A clinical interpretation of the importance to distinguish the within-person results and between-person results is lacking. Why is it relevant to separate them, apart from possible biases? What are the differences in interpretation for between-person results and within-person results? Examples are given for within-person results but these are not compared to between-person differences. How can separating both approaches help advance guidelines for future treatment options?

Our response:

Thank you for the comment. In the revised introduction, we have highlighted the statistical significance for distinguishing among the within-person and between-person findings (p. 5-6), in that “partitioning between among within- and between-person associations is important, because otherwise the results could be biased [34] or uninterpretable [35].”

Given that the literature has primarily shown between-person effects (p. 5-6), conclusions could only be drawn, specifically, in that when person *i* has a higher score in mindfulness than person *j*, then person *i* would have a higher score in emotion regulation and mental health than would person *j*. In this study, we argued that it is theoretically important to also address whether increases in mindfulness in person *i* are linked to his or her own better emotion regulation and mental health over time.

The present findings have important implications, as addressed in the Discussion.

At the within-person level (p. 21-22), practitioners could tailor their practices and interventions to the progress of each client. For example, to enhance subjective well-being and reduce psychological symptoms, practitioners can enhance within-person improvements in mindfulness and emotion regulation, as mindfulness affects mental health outcomes both directly and through adaptive emotion regulation. At the between-person level (p. 23), practitioners should be made aware of their clients' levels of mindfulness, emotion dysregulation, and mental health relative to other people. For example, returning to Figure 1, in relation to person *j*, person *i*'s greater level of mindfulness was associated with a lower level of emotion dysregulation and better well-being than person *j*, but not directly with fewer symptoms of depression and anxiety. Understanding these associations could help practitioners gauge the between-person importance or relevance of mindfulness in various mental health outcomes."

Comment:

The transition between the two paragraphs in this section is not very clear.

Our response:

Under the section 'Within- vs. Between-Person Effects, we have now strengthened the transition by adding the sentence, "[s]everal studies to-date have demonstrated the utility of within-person analyses in mindfulness and well-being."

Introduction: 'This study'

Comment:

Is the analysis technique novel or the used approach?

Our response:

We have deleted "novel" from "This Study" given that it has been used in previous research.

Comment:

Why is this unique? Elaborate in the paragraph above.

Our response:

In the revised manuscript, we have added (p. 6-7), "[a]s stated earlier, previous research predominantly examined between-person effects among mindfulness, emotion regulation, and mental health [5-11]. Although some studies have begun to examine within-person effects [36-38], few, if any, have teased apart within-person from between-person effects."

Comment:

The information ‘may be linked to covariates including time and gender’ is new information. A motivation for these choices could be added to the introduction.

Our response:

Thank you for the comment. On page 5, we have strengthened our arguments concerned with time by adding, “changes in mindful awareness were associated with subsequent changes of stress response and anxiety symptoms [28]. Similarly, changes in emotion regulation strategies were associated with changes in changes in depressive symptoms, life satisfaction, and general health over time [29]. As such, timing and changes were crucial in linking mindfulness, emotion regulation, and mental health outcomes.”

As for gender, previous research conducted with Chinese samples revealed that gender was associated with depression [39], anxiety [40], and subjective well-being [41]. As such, gender was included in this study as a covariate (p. 7).

Method

Comment:

Participants were at 18-27 years old: needs to be between. This information seems part of the result section (same as the retention rate).

Our response:

We have updated “[p]articipants were at 18-27 years old” to “[p]articipants were between 18 and 27 years old.” Since age and retention were not the study focus or part of the hypothesis, we have only included the information under “Participants” of the Method section (p. 7-8).

Comment:

What were the inclusion and exclusion criteria?

Our response:

We have added the following sentence (p.8), “Inclusion criteria included college-enrolled emerging adults who were proficient in Chinese, within the age range between 18 and 29 years old [42], who agreed to participate for three time points over the course of a year.”

Comment:

Which number of participants was aimed at?

Our response:

Thank you for the comment. We have conducted a post hoc power analysis based on Monte Carlo simulations [76]. Based on the estimated parameter values from the multilevel mediation analysis, we repeatedly simulated 1000 new data sets and fitted

the same multilevel mediation model to these simulated data sets. By counting the proportion of significant results, we obtained the empirical post hoc power. The results showed that on average, the post hoc power for the six indirect effects considered in the study (three at the within-person level and three at the between-person level) was 80.2%, suggesting that it was adequate to aim for 191 participants.

Reference:

76. Gelman A, Hill J. Data analysis using regression and multilevel/hierarchical models. Cambridge: Cambridge University Press; 2006.

Comment:

How many emails were send out?

Our response:

Two mass emails were sent out (p. 7-8).

Comment:

The begin structure is unclear; some information from the procedure part belongs to a part about participants and vice versa.

Our response:

We have worked to ensure that the Participants and Procedures sections were independent from each other (p. 7-8). Specifically, the Participants section involves recruitment method, inclusion criteria, retention rate, and demographics. The Procedures section involves ethics approval information, informed consent details, and data collection procedures.

Comment:

What sort of informed consent was given?

Our response:

We have revised the Procedures section by stating the following (p. 8), “The study was approved by the Human Research Ethics Committee of the first author’s university prior to its implementation. All procedures performed were in accordance with the ethical standards of the institutional research committee and the 1964 Helsinki declaration and its later amendments. Informed consent to participate in the present study was obtained prior to the administration of the questionnaire.”

Comment:

How much time did participants investigate in the study?

Our response:

We have now included the following information (p. 8), "[e]ach packet took approximately 30 minutes to complete."

Method: Measures

Comment:

This section could benefit from a description of the reliability and validity of the used scales.

Our response:

Based on previous research, we have now included the reliability and validity of the scales used in the present study (p. 8-12).

Comment:

The timeframes of the mental health questionnaires differ (one assesses the past week, another the past month). Could this have influenced the findings in any way?

Our response:

We agree with Reviewer #1 that the timeframes of the mental health questionnaire differ. As a limitation, we have now stated (p.24), "the timeframes of the mental health questionnaires differ. For example, the PHQ-9 assessed depressive symptoms in the past two weeks, whereas the Mental Health Continuum Short Form assessed emotional, social, and psychological well-being in the past four weeks, whereas the MHC-SF assessed emotional, social, and psychological well-being in the past four weeks. Future research may consider standardizing the timeframes to increase precision of the variables."

Comment:

Anxiety symptoms: was used to measure anxiety, not depression.

Our response:

Thank you for the comment. We have made relevant changes in the revised manuscript (p. 11).

Method: Data analysis

Comment:

The general writing style of this section is suggestive, which results in unclearity when reading.

Our response:

We have expanded and revised the Data Analysis section. A more affirmative writing style has been used.

Comment:

Why are there no details provided on the multilevel mediation model used here (now upon request)? Was growth curve modelling used? Some information would better fit the introduction.

Our response:

Thank you for the comment. We have now provided a statistical description of the multilevel mediation model (p. 12-15). The multilevel mediation model used in the analysis was not a growth curve model. To avoid confusion, we have removed sentences related to growth curve modeling in the revised manuscript. Two major differences between multilevel mediation model and growth curve model are as follows:

First, in our analysis, the relationship between the independent variables and the mediator (i.e., reflected by the “a” path) and the relationship between the mediator and the dependent variables (i.e., reflected by the “b” paths) were estimated simultaneously. In growth curve modeling with time-varying covariates, however, these relationships were estimated separately. The estimated “a” path and “b” paths could be correlated when data are nonnormal and when robust methods are used (i.e., when MLR is used in Mplus; see the discussion in http://www.statmodel.com/discussion/messages/11/9365.html?1396831771). In our analysis, MLR was used and the possible association between the estimated “a” path and “b” path could be captured. In contrast, the association could not be captured using growth curve modeling. This might have an impact on the inferences on the indirect effect, which was defined as the product of the “a” path and the “b” path (e.g., see [77; p. 92]; and the explanation regarding the difference between the delta method used in Mplus and Sobel test in http://www.statmodel.com/discussion/messages/11/9365.html?1396831771).

Second, we relied on the multilevel SEM approach [34] to separate the within- and between-person components of variables. In contrast, as a typical multilevel modeling, growth curve modeling relies on group centering to fulfill this objective. As discussed in [34], the multilevel approach was relatively more biased.

Reference:

77. MacKinnon DP. Introduction to statistical mediation analysis (Multivariate applications series). Taylor & Francis Group/Lawrence Erlbaum Associates; 2008.

Comment:

Please specify the used levels.

Our response:

We have now specified the used levels in greater detail.

Comment:

Separate results from methods.

Our response:

Thank you for the comment. The results have been separated from methods.

Comment:

The section on the mediation path is difficult to follow.

Our response:

Thank you for the comment. We have revised the Data Analysis section. Now the logic of this section flows more naturally and it is easier to understand. Specifically, we first described the statistical approach to multilevel mediation used in this study (p. 12). We then explained what covariates and why they were considered in the model (p. 12). Next, we explained why some paths were fixed and some was allowed to be random across participants (p.13). We also described in detail the multilevel mediation model used in the analysis and explained the practical meanings of relevant model parameters (p. 13-15). Finally, we explained why the proportion of indirect effect was not calculated (p. 15).

Comment:

The last part of this section is especially unclear, does this mean that the used analysis require not much faith? Why is this not mentioned in the discussion?

Our response:

Thank you for the comment. The last sentence of the Data Analysis section (p. 15), "[u]nless the sample size was fairly large, Hayes [60, p.189] recommended not 'having much faith' in this measure" explains the reason why we did not compute the ratio of indirect effect to the total effect. It was not related to the method used in the analysis.

Results

Comment:

When reading this section I would first expect general sample information, including cores on all outcome measures. Then correlational information... Before considering correlations, I am curious about how the population scored on the

measures. To aid the interpretation, it would be good to mention some reference points for ranges. For example, to interpret the 2.85 score on emotion regulation, I would like to know what this means. What score reflects serious difficulties with emotion regulation?

Our response:

Thank you for the comment. We have now briefly described the correlational findings at the beginning of the results section (p. 15).

As for how the current sample scored on the measures compared to other study samples, we have now included the information under the measures section (p. 8-12). Specifically, compared to other Chinese samples, participants from this study had similar mean levels of dispositional mindfulness [44], emotion dysregulation [46], and symptoms of depression and anxiety [50]. Nevertheless, they had greater mean levels of subjective well-being [56]. This information has also been included in the discussion section (p. 24).

Comment:

Table 1: This table shows many results at the same time. While it seems correct, it might be easier for readers to separate the correlations from the means and standard deviations of the outcomes (make two tables).

Our response:

We have separated Table 1 into two tables according to Reviewer #1's recommendation. Table 1 shows the means, standard deviations, ranges, ICCs, and design effects, whereas Table 2 shows the zero-order correlations.

Comment:

Gender does not make sense as M and SD.

Our response:

We have removed the *M* and *SD* of gender in Table 1.

Comment:

The within-person correlations are not part of the analyses section, a clear rational for these results does not follow naturally.

Our response:

Thank you for the comment. We have revised the Data Analysis section (p. 12-15). Now the subsection contains a detailed description on the multilevel mediation model. The analyses regarding the within- and between-person associations have also been described.

Results: 'Multilevel Structural Equation Modeling'

Comment:

Are the design effect results reported somewhere?

Our response:

The design effect results have now been reported in Table 1.

Comment:

Some information in this section would fit the analyses section better.

Our response:

To enhance clarity and organization, we have restructured the Data Analysis and the Results sections (p. 12-20).

Comment:

Results are better presented numerically, an interpretation of the variances explained suites the discussion (interesting information). They are part of figure 1, might be good to mention this earlier in the section.

Our response:

Thank you for the comment. As we were unable to denote the % of variance in Figure 1, we did not describe the figure early on. However, at the end of the paragraph, we have recommended the readers to refer to Figure 1 for specific path coefficients.

Results: 'Within-person indirect Effect of dispositional mindfulness (and between)'

Comment:

How do the results mentioned here relate to the heading title of indirect effects (they mention direct effects)?

Our response:

Thank you for the comment. The section refers to indirect effects.

Comment:

Table 3: Please explain the arrows and the c'w1i notations in the notes.

Our response:

Thank you for the comment. We have added an explanation in note on Table 3 to explain the notations.

Comment:

*Table 4: The explanation helps. Some of these elements could be taken into the table, providing a heading for within and between analyses results. Consider keeping the format similar to the other tables (e.g., using * instead of boldfaced numbers for*

significance).

Our response:

We have revised Table 4 by using * instead of boldfaced numbers for significant numbers.

Discussion

Comment:

The first section clearly summarizes the results. This part could benefit from an integrated interpretation of the findings. For example, what could be the reason for the surprising finding of time?

Our response:

Thank you for the comment. At the beginning of the Discussion, we have included a potential reason for the surprising finding of time (p. 21) by stating, "potentially due to an increasing level of academic stress over the school year." To ensure that the opening paragraph is clear and succinct, we have explained the findings in greater detail in the subsequent paragraphs.

Comment:

The statements 'findings advance the field by using a process-oriented approach' and 'demonstrates the utility of multilevel modeling' could use more explanation.

Our response:

Thank you for the comment. To ensure that our discussion is clear and informative, the statement, "findings advance the field by using a process-oriented approach," have been revised to "[t]hese findings advanced the field by establishing within- and between-person relations between dispositional mindfulness and mental health via emotion dysregulation." (p.21)

The statement, "demonstrates the utility of multilevel modeling" have been revised to "[t]his study used multilevel modeling by partitioning between-person relations from within-person relations."(p.21)

Comment:

The within-person and between-person definitions (when mindfulness of person i increases etc.) can be left out; this is sufficiently clear from the introduction.

Our response:

We thank Reviewer #1 for the recommendation. We have removed the within-person definition in the discussion. To concretely explain the between-person findings and their clinical significance, however, we have decided to keep the notations (e.g., person *i* vs person *j*; p. 22-23).

Comment:

The section on between-person findings with emotion regulation as an explanatory variable is confusing because of the sentence structures.

Our response:

We have now revised the section (p.22-23).

Comment:

Please elaborate on 'stage-salient variables'.

Our response:

Thank you for the comment. Stage-salient variables refer to variables specific to a certain developmental period, i.e., in our case, emerging adulthood. In the discussion, we have added, "other stage-salient variables in emerging adulthood, such as having increasing responsibility, becoming more financial independent, and having a greater commitment in romantic relationships [73], may be more strongly associated with positive well-being." (p.23)

<u>Reviewer #2</u>

Comment:

This paper investigates whether emotion regulation is a mediator between mindful awareness and depression, anxiety, and subjective wellbeing in Chinese young adults. Findings suggest that emotion regulation mediates the association between mindful awareness and wellbeing both at the within-person and the between-person level, with the exception that emotion regulation did not mediate the between-person association between mindfulness and subjective wellbeing.

This is an interesting and well-written paper. I only have a few minor comments.

Our response:

We thank Reviewer #2 for the positive comments.

Minor Comments

Comment:

I would prefer the term 'sum score' or 'total score' rather than 'composite score' (p. 14) to reflect the total score on a single questionnaire, given that composite score is usually reserved for a score that reflects a combination of scales.

Our response:

We have revised the method by changing "composite scores" to "mean scores." (p.8-12)

Comment:

“The item scores were summed to form a composite score of depressive symptoms”. However, Table 1 appears to report mean scores rather than sum scores (on a scale from 0 to 3 instead of the original scale from 1 to 4?).

Our response:

Thank you for the comment. We apologize for the confusion. In this study, participants rated on a scale from 0 to 3 for each item of the PHQ-9. Afterwards, the item scores were averaged to form a score. The description has now been updated in the manuscript (p. 10).

Comment:

For subjective wellbeing, unlike the other scales, it is not explicitly stated how the score was calculated (mean score of the 14 items?).

Our response:

We have now stated how the subjective well-being score was calculated (p. 12), “The item scores were averaged to form a mean score, with higher scores indicating better well-being.”

Comment:

In Table 1 the mean for emotion regulation is 2.97 but in the supplementary data set it appears to be 3.97 if I am not mistaken.

Our response:

Thank you for pointing this out. We have corrected the means and standard deviations for emotion dysregulation.

Comment:

Please explain ‘stage-salient variables’ (p. 24). Do have some suggestions as to which stage-salient variables may be relevant to include in future research?

Our response:

Thank you for the comment. Stage-salient variables refer to variables specific to a certain developmental period, i.e., in our case, emerging adulthood. In the discussion, we have added (p.23), “other stage-salient variables in emerging adulthood, such as having increasing responsibility, becoming more financial independent, and having a greater commitment in romantic relationships [73], may be more strongly associated with positive well-being.”

6. The manuscript contains a few typos:

- p. 10 stwell-being

- p. 14 "how often did you feel that that you had …."
- p. 14 "A sample items included" "
- p. 14 The 7-item Generalized Anxiety Disorder - 7 ... was used to measure depressive symptoms". Depressive symptoms should be anxiety symptoms.
- p. 13 "The 15-item Mindful Attention Awareness Scale …. on a 6-point scale from 1 (almost always) to 5 (almost never)". This should say 'a scale from 1 to 6'?

Our response:

Thank you for the comment. The typos have been removed in the revised manuscript.

Reviewer #3:

Title Page

Comment:

Title arose some expectations that were not accomplished later.

Our response:

Thank you for the comment. We have updated the title to, "Dispositional Mindfulness and Mental Health in Chinese Emerging Adults: A Multilevel Model with Emotion Dysregulation as a Mediator."

Comment:

DERS: Difficulties in ER Scale measures emotional dysregulation rather than emotion regulation. Dysregulation: A failure to regulate properly.

Our response:

We agree with Reviewer #3's comment. In our original manuscript, the scores of the negative items of DERS were reversed, such that the final scores reflected a greater ability in emotion regulation.

Based on Reviewer #3's comment, we have unreversed the scores, such that higher scores reflected greater emotion dysregulation. Where appropriate, we have now used "emotion dysregulation" in the revised manuscript.

Comment:

Many authors have pointed out that these mindful scales are not measuring mindful activities, they assess dispositional mindful activities.

Our response:

We agree with Reviewer #3's comment and have changed our wordings to "dispositional mindfulness" in describing the variable under study.

Abstract

Comment:

Provide here M and SD for participants' age.

Our response:

We have revised the abstract by adding *M* and *SD* of the participants' age.

Comment:

A questionnaire about what?

Our response:

We have added that the questionnaire assessed their dispositional mindfulness, emotion dysregulation, and mental health outcomes.

Introduction

Comment:

Avoid using regulating emotions in the definition of emotion regulation.

Our response:

We have revised the revised the sentence to the following, "[e]motion regulation is defined as the process in modulating emotions and emotional responses [14-15]." (p.3)

Introduction: Within- vs. Between-Person Effects

Comment:

Agree but this section above is a little bit tangential to your research. You did not implement a RCT MBI.

Our response:

We apologize about the confusion and agree with Reviewer #3 that we did not implement a mindfulness-based intervention. Given that very few, if any, studies assessed within- vs. between-person effects of dispositional mindfulness on mental health, we have reviewed mindfulness-based intervention studies using a multilevel modeling approach.

Method

Comment:

Does this Committee a code?

Our response:

We have revised the Method by stating (p. 8), "The study was approved by the Human Research Ethics Committee of the first author's university institution prior to its implementation. All procedures performed were in accordance with the ethical standards of the institutional research committee and the 1964 Helsinki declaration

and its later amendments.”

Comment:

The sample is not enough, gender was not balanced, there was no intervention…

Our response:

We agree with Reviewer #3 that the sample size could have been larger and gender could be more balanced.

In terms of sample size, we have conducted a post hoc power analysis based on Monte Carlo simulations [76]. Based on the estimated parameter values from the multilevel mediation analysis, we repeatedly simulated 1000 new data sets and fitted the same multilevel mediation model to these simulated data sets. By counting the proportion of significant results, we obtained the empirical post hoc power. The results showed that on average, the post hoc power for the six indirect effects considered in the study (three at the within-person level and three at the between-person level) was 80.2%, suggesting that it was adequate to aim for 191 participants.

As for gender, we have stated in the Discussion (p. 23), “our small sample of men (i.e., 9.95%) precluded us from drawing meaningful conclusions about gender as a correlate of psychological distress.” We have also acknowledged, (p. 24), “our participants were mainly female. Future studies with gender-balanced samples are necessary to draw meaningful conclusions for the effects of gender.”

We understand that intervention studies are necessary to improve mental health. However, it is beyond the scope of the present study. As a future direction, we have added (p. 24), “researchers should translate the present findings and design targeted interventions to improve mental health outcomes.”

Reference:

76. Gelman A, Hill J. Data analysis using regression and multilevel/hierarchical models. Cambridge: Cambridge University Press; 2006.

Comment:

This tool measures dispositional mindful awareness.

Our response:

We have now updated the description to the following (p.8), “[t]he 12-item Cognitive and Affective Mindfulness Scale – Revised (CAMS-R) [43] was used to assess dispositional mindfulness on a 4-point scale from 1 (*rarely/ not at all*) to 4 (*almost always*).” The measure, CAMS-R, broadly assessed dispositional mindfulness, in addition to dispositional mindful awareness.

Comment:

Cronbach's alpha of subscales should be also reported. You can use intercorrelations at T1, T2 and T3 as well.

Our response:

Thank you for the comment. We have now indicated the Cronbach's alpha of subscales for the Cognitive and Affective Mindfulness Scale – Revised (p. 9), the Difficulties in Emotional Regulation Scale (p. 9), and the Mental Health Continuum Short Form (p. 12). The intercorrelations between T1, T2 and T3 have been reported for all measures as well (p. 8-12).

Comment:

Is there a cut-off in the scoring to indicate depression?

Our response:

Thank you for the question. The cut-off scores for the Patient Health Questionnaire-9 and the Generalized Anxiety Disorder-7 measures have now been reported on page 10.

Results

Comment:

Gender: 0 male and 1 female?

Our response:

We have now denoted “0=male; 1=female” on Tables 1-3.

Comment:

Why are the correlations of mindfulness moderate / low at T1, T2, T3?

Our response:

The correlations of dispositional mindfulness between T1 and T3 were .53 - .58 over time, *p*s < .001, suggesting moderate effect sizes [62]. Even though the scale was intended to measure stable and dispositional mindfulness, our data suggest potential changes over time.

Comment:

Based on the correlations, gender could be a moderator.

Our response:

We agree with Reviewer #3’s comment. However, our small sample of men (i.e., 9.95%) precluded us from drawing meaningful conclusions about gender as a moderator. As a limitation, we have stated (p. 24), “our participants were mainly female. Future studies with gender-balanced samples are necessary to draw

meaningful conclusions for the effects of gender."

Comment:

M and SD seem to be averaged, this should be notified in the table and put in the first column the range of every single instrument.

Our response:

We have now stated (p. 15), "Table 1 summarizes the means, *SD*s, ranges, ICCs, and design effects."

Comment:

ICC is more appropriate to assess the level of agreement among raters. Where is reported the ICC, Table 1 is not a ICC.

Our response:

Thank you for the comment. We have added ICCs in Table 1 for all the variables under study.

Discussion

Comment:

You did not implement an intervention... The discussion needs to follow a tentative argument… The undergraduate sample is too biased for the conclusions (should be tentative rather than so affirmative). Discussion can be more tentative than affirmative due to data did not allow those conclusions.

Our response:

Thank you for the comment. We agree with Reviewer #3 that the present study involved no experiments and interventions. Thus, in the discussion, we have avoided wordings such as "causes", "affects", or "influences." Based on the present findings, tentative arguments have been made, without biases or exaggerations. As a limitation, we have added, "researchers should translate the present findings and design targeted interventions to improve mental health outcomes."
